# Sex differences in treatment of familial hypercholesterolaemia: a meta-analysis

**DOI:** 10.1093/eurheartj/ehae417

**Published:** 2024-07-08

**Authors:** Iulia Iatan, Leo E Akioyamen, Isabelle Ruel, Amanda Guerin, Lindsay Hales, Thais Coutinho, Liam R Brunham, Jacques Genest

**Affiliations:** Department of Medicine, Centre for Heart Lung Innovation, Providence Health Care, University of British Columbia, Vancouver, British Columbia, Canada; Department of Medicine, University of Toronto, Toronto, Ontario, Canada; Department of Medicine, Research Institute of the McGill University Health Centre, 1001, Decarie blvd. Office EM1.2212, Montreal, Quebec H4A 3J1, Canada; Department of Medicine, Research Institute of the McGill University Health Centre, 1001, Decarie blvd. Office EM1.2212, Montreal, Quebec H4A 3J1, Canada; McGill University Health Center Libraries, Montreal, Quebec, Canada; Department of Cardiovascular Medicine, Mayo Clinic, Rochester, MN, USA; Department of Medicine, Centre for Heart Lung Innovation, Providence Health Care, University of British Columbia, Vancouver, British Columbia, Canada; Department of Medicine, Research Institute of the McGill University Health Centre, 1001, Decarie blvd. Office EM1.2212, Montreal, Quebec H4A 3J1, Canada

**Keywords:** Familial hypercholesterolaemia, Atherosclerotic cardiovascular disease, Lipid-lowering treatment, Sex differences, Females, Systematic review

## Abstract

**Background and Aims:**

Familial hypercholesterolaemia (FH) is a highly prevalent monogenic disorder characterized by elevated LDL cholesterol (LDL-C) levels and premature atherosclerotic cardiovascular disease. Sex disparities in diagnosis, lipid-lowering therapy, and achieved lipid levels have emerged worldwide, resulting in barriers to care in FH. A systematic review was performed to investigate sex-related disparities in treatment, response, and lipid target achievement in FH (PROSPERO, CRD42022353297).

**Methods:**

MEDLINE, Embase, The Cochrane library, PubMed, Scopus, PsycInfo, and grey literature databases were searched from inception to 26 April 2023. Records were eligible if they described sex differences in the treatment of adults with FH.

**Results:**

Of 4432 publications reviewed, 133 met our eligibility criteria. In 16 interventional clinical trials (eight randomized and eight non-randomized; 1840 participants, 49.4% females), there were no differences between males and females in response to fixed doses of lipid-lowering therapy, suggesting that sex was not a determinant of response. Meta-analysis of 25 real-world observational studies (129 441 participants, 53.4% females) found that females were less likely to be on lipid-lowering therapy compared with males (odds ratio .74, 95% confidence interval .66–.85). Importantly, females were less likely to reach an LDL-C < 2.5 mmol/L (odds ratio .85, 95% confidence interval .74–.97). Similarly, treated LDL-C levels were higher in females. Despite this, male sex was associated with a two-fold greater relative risk of major adverse cardiovascular events including myocardial infarction, atherosclerotic cardiovascular disease, and cardiovascular mortality.

**Conclusions:**

Females with FH were less likely to be treated intensively and to reach guideline-recommended LDL-C targets. This sex bias represents a surmountable barrier to clinical care.

## Structured Graphical Abstract

**Structured Graphical Abstract:**
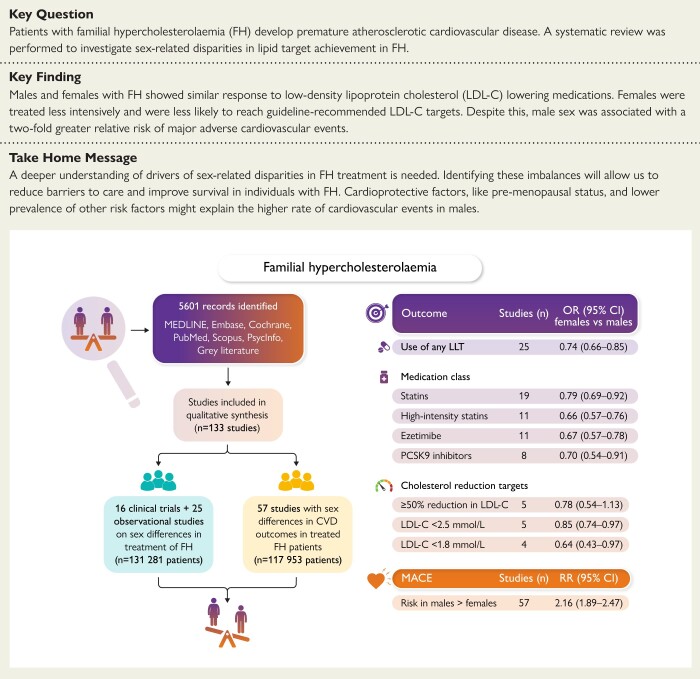
Sex differences in the treatment of familial hypercholesterolaemia: a systematic review and meta-analysis. CI, confidence interval; CVD, cardiovascular disease; FH, familial hypercholesterolaemia; LDL-C, low-density lipoprotein cholesterol; LLT, lipid-lowering therapy; MACE, major adverse cardiovascular events; OR, odds ratio; PCSK9, proprotein convertase subtilisin/kexin type 9; RR, risk ratio.

**See the editorial comment for this article ‘Familial hypercholesterolaemia: need for equitable treatment in women and men’, by R.D. Santos, https://doi.org/10.1093/eurheartj/ehae464.**

## Introduction

Familial hypercholesterolaemia (FH) is a highly prevalent monogenic disorder characterized by lifelong elevated blood levels of LDL cholesterol (LDL-C). Its worldwide prevalence is 1/311 with an estimated 25–30 million people affected globally.^1^ Left untreated, it leads to premature atherosclerotic cardiovascular disease (ASCVD), particularly coronary artery disease (CAD), in addition to greater medical costs and a reduced health-related quality of life.^2–4^ Prompt recognition and treatment with statins and other lipid-lowering therapies (LLTs) is highly efficacious and can normalize life expectancy.

Despite this, FH remains under-recognized and under-treated worldwide. The reasons for under-treatment remain incompletely understood, and there is limited information on barriers to care in FH. As an autosomal semi-dominant trait, FH affects males and females equally. Yet, there is growing recognition that sex may play a role in the clinical presentation and management of this illness, contributing to barriers to care. Increasing reports from our groups and others suggest that female patients with FH may have an increased burden of LDL-C compared with males, are diagnosed later, treated less aggressively with guideline-mandated medical therapies, and are less likely to reach recommended LDL-C targets or thresholds.^5–8^ These treatment differences were observed in both adults and children from the Familial Hypercholesterolaemia Studies Collaboration (FHSC), the largest global registry of patients with FH worldwide.^6,9^ Furthermore, whether sex is an independent predictor of outcomes in FH remains debated. While some studies have demonstrated greater ASCVD in males,^6^ others have shown no difference or greater risk in females.^10–12^ The FHSC reported that in 42 167 patients from 56 countries (53.6% females) the prevalence of CAD (17.4%), increasing with untreated LDL-C levels, was two times lower in females than in males, and the overall ASCVD risk was lower in index and non-index females.^6^ Despite this, females with FH are at greater risk of CAD mortality than non-FH females. Additionally, the same FH diagnostic criteria are used for both males and females, without consideration of variability of LDL-C levels by sex throughout life, especially during pregnancy and in post-menopausal years.

In order to better understand the scope and extent of this question, we aimed to characterize sex-related disparities in management and ASCVD in patients with FH. To address this, we conducted a systematic review and meta-analysis of studies examining the associations between sex and treatment, response, achieved LDL-C levels, and guideline-recommended targets in FH, both in clinical trials to detect whether response to fixed doses of LLT differs between sexes and in real-world observational data from registries and cohort studies. Associations between sex and cardiovascular disease (CVD) risk among patients with FH were subsequently investigated.

## Methods

### Protocol and registration

This systematic review study was registered in the PROSPERO prospective database for systematic reviews (CRD42022353297) and reported in accordance with the Preferred Reporting Items for Systematic reviews and Meta-Analyses (PRISMA),^13^ Meta-analysis Of Observational Studies in Epidemiology (MOOSE),^14^ and Sex and Gender Equity in Research (SAGER)^15^ consensus statements.

### Information sources and search strategy

Search strategies were developed and executed with the assistance of a medical librarian (L.H.) with expertise supporting systematic reviews. Database searches were completed for MEDLINE, Embase, The Cochrane library, PubMed, Scopus, and PsycInfo. Clinical trial registries including ClinicalTrials.gov, the International Clinical Trials Registry Platform, UK Clinical Trials Gateway, and the ProQuest Dissertations and Theses database were also searched. Grey literature was sourced from Google Scholar and Open Grey. Reference lists of relevant systematic reviews were also searched for additional citations. No language limits were applied. Searches were conducted from database inception to 21 July 2020; the Medline search was rerun prior to manuscript preparation in 26 April 2023. A complete description of the search strategy is provided in the Supplementary data online, Appendices. The authors acknowledge that while ‘female’ or ‘male’ refer to an individual's biological sex and ‘woman’ or ‘man’ refer to an individual's gender, historically these terms have been used interchangeable in the literature; all these terms were included in the search strategy. However, in the present study the terms ‘female’ or ‘male’ are used for consistency as it pertains to biological sex.

### Study selection and eligibility criteria

Candidate titles, abstracts, and full-text articles were evaluated in duplicate by five independent reviewers (A.G., J.G., I.I., I.R., L.E.A.) using Rayyan systematic review software (www.rayyan.ai). Disagreements were resolved by discussion to consensus. Studies were considered eligible for inclusion if they: (i) were interventional and/or observational studies in adult participants (age ≥18 years) with heterozygous FH (diagnosed using genetic and/or common clinical criteria) and (ii) reported data separately for male and female participants on our outcomes of interest, as described below. Non-human studies, case reports, editorials, conference abstracts, and narrative reviews were excluded. Any clinical definition of FH used in studies was accepted.

### Outcomes

Our primary outcome consisted of the number of females vs. males treated with any LLTs in the included studies. Treatment with specific drug classes where studies reported them [statins, ezetimibe, proprotein convertase subtilisin/kexin type 9 (PCSK9) inhibitors] was also examined. The secondary outcomes consisted of: (i) absolute and relative reductions in LDL-C experienced by male and female patients with FH treated with LLTs and (ii) attainment of guideline-recommended LDL-C reduction targets in these patients (defined as ≥50% reductions in LDL-C from baseline, LDL-C < 2.5 mmol/L, LDL-C < 1.8 mmol/L). Sex-specific differences in fatal and non-fatal major adverse cardiovascular events (MACE) were examined afterwards.

### Data extraction

Data were extracted from studies deemed to meet eligibility criteria by at least two independent reviewers (A.G., J.G., I.I., I.R., L.E.A.). These included details on general study characteristics (first author, design, recruitment period, duration of follow-up); information about the studied population (mean age, number and proportion of males and females, diagnostic and treatment characteristics); and information on the outcomes in the study. Characteristics of studies were summarized in tabular format and narratively synthesized. Unadjusted and adjusted measures of relative risk and 95% confidence intervals (CIs) were also extracted. Maximally adjusted risk measure that was available from studies and risk estimates corresponding to the longest duration of follow-up were used. Study authors were not contacted for additional data. Quality of eligible studies was assessed using the Effective Public Health Practice Project (EPHPP) Quality Assessment Tool for quantitative studies after assessing the following domains: selection bias, study design, confounders, blinding, data collection method, and withdrawals and dropouts.^16^ Generally, the global rating of a study was considered to be strong if none of the quality domains were rated as weak; moderate if one domain was rated as weak; and weak if two or more domains were rated as weak.

### Statistical analyses

Meta-analyses were conducted using inverse-variance models incorporating random effects estimated using the method of DerSimonian and Laird.^17^ For dichotomous outcomes, pooled estimates of effect size were calculated as odds ratios (OR) with 95% CIs. Weighted mean differences were calculated for continuous outcomes. Summary estimates were displayed graphically with forest plots. Heterogeneity was assessed using the *I*^2^ statistic; heterogeneity was interpreted using the following thresholds: 0%–40%: might not be important; 30%–60%: may represent moderate heterogeneity; 50%–90%: may represent substantial heterogeneity; and 75%–100%: considerable heterogeneity. Publication bias was assessed visually using funnel plots, and where analyses included >10 studies, formally using Egger’s weighted regression and significance set at *P* < .10.^18^ If publication bias was present, we used the trim-and-fill method to control for publication bias. This technique may not be entirely suitable when excess heterogeneity is present,^19^ and we therefore also reported heterogeneity using the *I*^2^ statistic (see Limitations). Sources of heterogeneity in our primary outcome were investigated through subgroup analyses and meta-regression. Subgroups were stratified by: year of publication (set at before and after 2016 to represent the introduction of ICD-10 codes for FH), FH diagnosis criteria (majority (>50%) with genetic testing, 100% with clinical criteria, combination of minority (<50%) with genetic testing and remaining with clinical criteria, and 100% using LDL-C cut-offs), study sample size (fewer or more than 1000 participants), and World Health Organization (WHO) geographical location (Americas, Europe, Western Pacific, and International). χ^2^ statistical test was used to detect differences between subgroups. Univariate meta-regression was performed to explore potential sources of heterogeneity using the following covariates: year of publication, age of participants, proportion of females in included studies, mean LDL-C reduction, and the proportion of individuals with CVD. One study included in the analyses sourced data from multiple national registries,^6^ creating the potential for overlap with multiple cohorts; accordingly, additional sensitivity analyses in which this study was excluded were conducted. Meta-regressions were conducted using a mixed-effects approach to account for between- and within-study heterogeneity, with restricted maximum likelihood estimation of between-study variance. For subgroup and meta-regression analyses, two-sided *P*-values <.05 were considered significant. Additional details of our analyses are described in the Supplementary data online, *Appendix*. Analyses were performed in Review Manager 5.4 and RStudio (version 2023.03.0 + 386).

## Results

**Figure 1:**
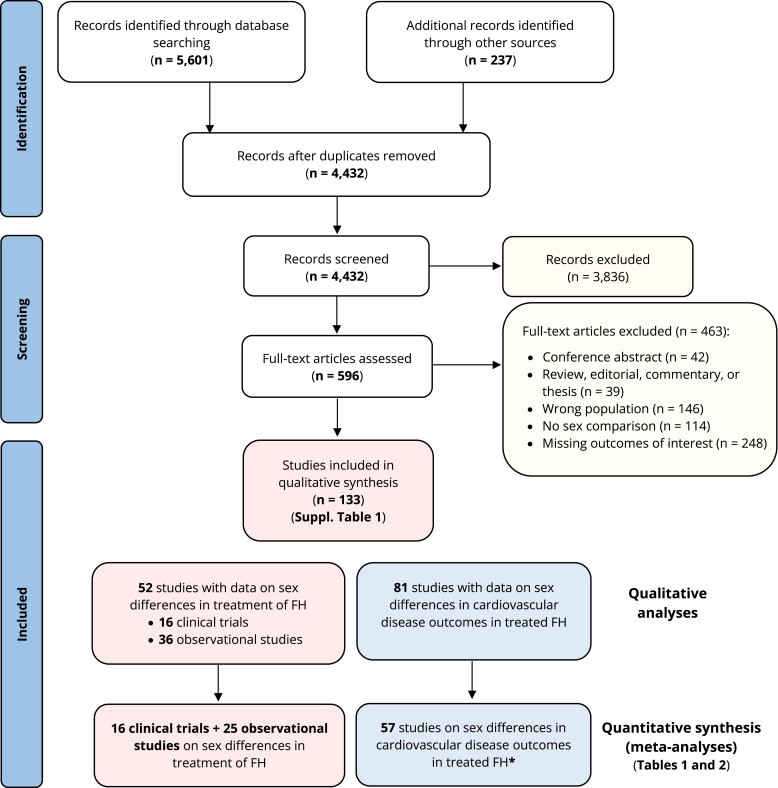
Preferred Reporting Items for Systematic reviews and Meta-Analyses flow chart of studies included in the systematic review of sex differences in the treatment of familial hypercholesterolaemia. *Seven studies were describing both data on sex differences in the treatment of familial hypercholesterolaemia and cardiovascular disease outcomes in patients with treated familial hypercholesterolaemia. FH, familial hypercholesterolaemia

Database searches identified 5601 records which were reduced to 4432 following duplicates removal. From initial abstract screening, 3836 studies were excluded, and a total of 596 full-text articles were reviewed. Of these, 133 studies met criteria for sex differences in the treatment of FH and were included in the qualitative analyses (*Figure 1*). These studies comprised 16 interventional clinical trials testing a lipid-lowering agent (eight randomized and eight non-randomized clinical trials), 36 observational studies presenting data on sex differences in FH treatment, and 81 observational studies on sex differences in CVD outcomes. Observational studies were prospective, retrospective, or cross-sectional cohort studies. Characteristics of all studies are shown in Supplementary data online, *Table S1*, while a risk of bias per study is presented in Supplementary data online, *Appendix S3*. When evaluated by the EPHPP tool, most clinical trials were rated as being moderate, while observational studies ranged from moderate to strong, with the greatest threats to validity being because of study design or blinding.

**Figure 2:**
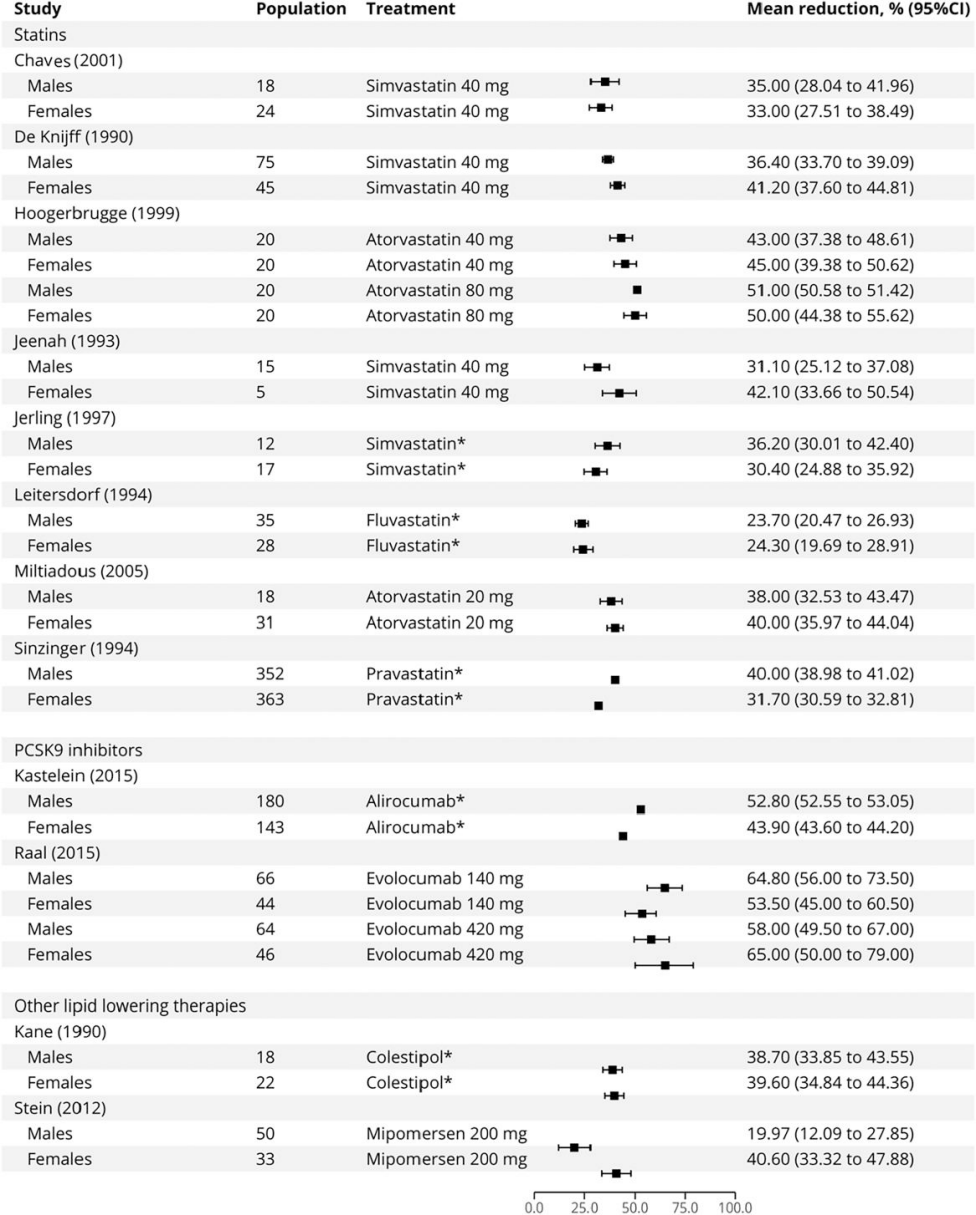
Mean LDL cholesterol percent reduction in clinical trials included in the systematic review. Asterisks denote studies where participants received progressive escalations of therapy to reach maximal doses. CI, confidence interval; PCSK9, proprotein convertase subtilisin/kexin type 9

There were 16 clinical trials of LLTs in which an analysis by sex was provided (1840 participants; 49.4% females). In 12 studies in which a mean percent LDL-C reduction value was available, there were no differences between males and females in response to fixed doses of LLTs (*Figure 2*), suggesting that patient sex was a not a determinant of therapeutic response. Absolute LDL-C reductions in males and females from the reviewed clinical trials are reported in Supplementary data online, *Figure S1*, while mean LDL-C reductions from LLT are shown in Supplementary data online, *Figure S2*.

**Figure 3:**
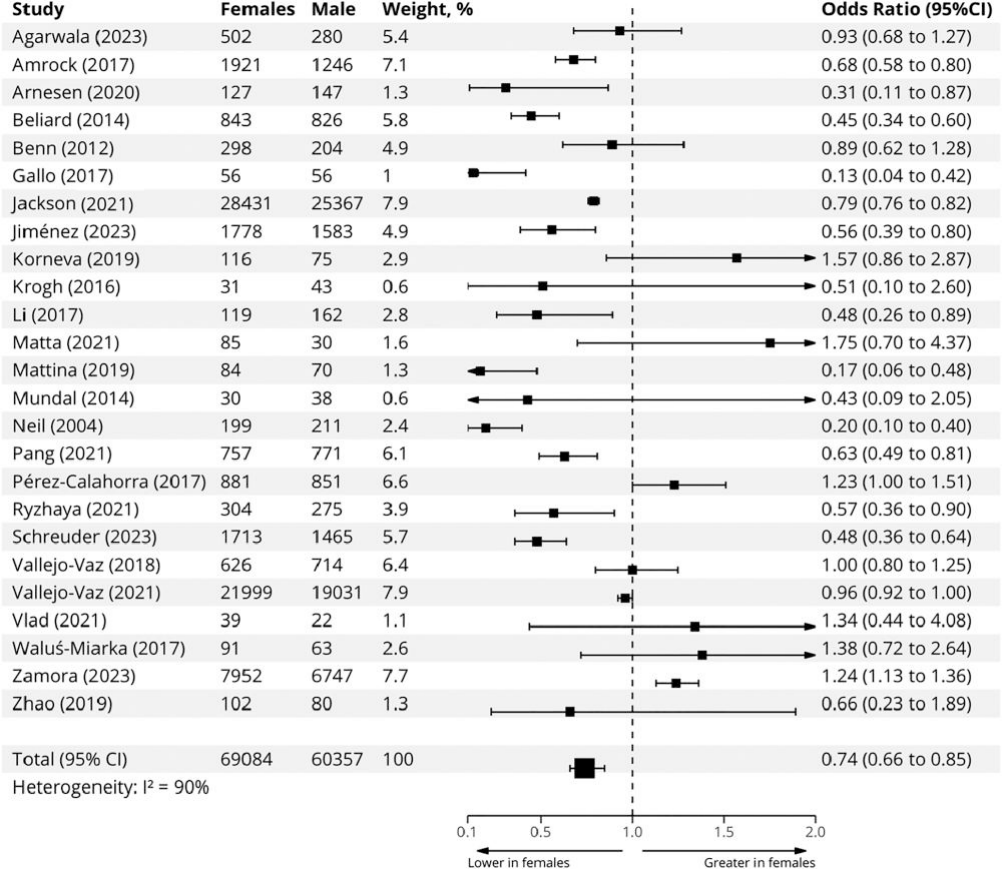
Meta-analysis of sex differences in treatment with lipid-lowering therapies in observational studies. Squares represent study-level odds ratios; horizontal lines represent 95% confidence intervals; large square represents pooled odds ratio derived under the random-effects model. CI, confidence interval

**Figure 4:**
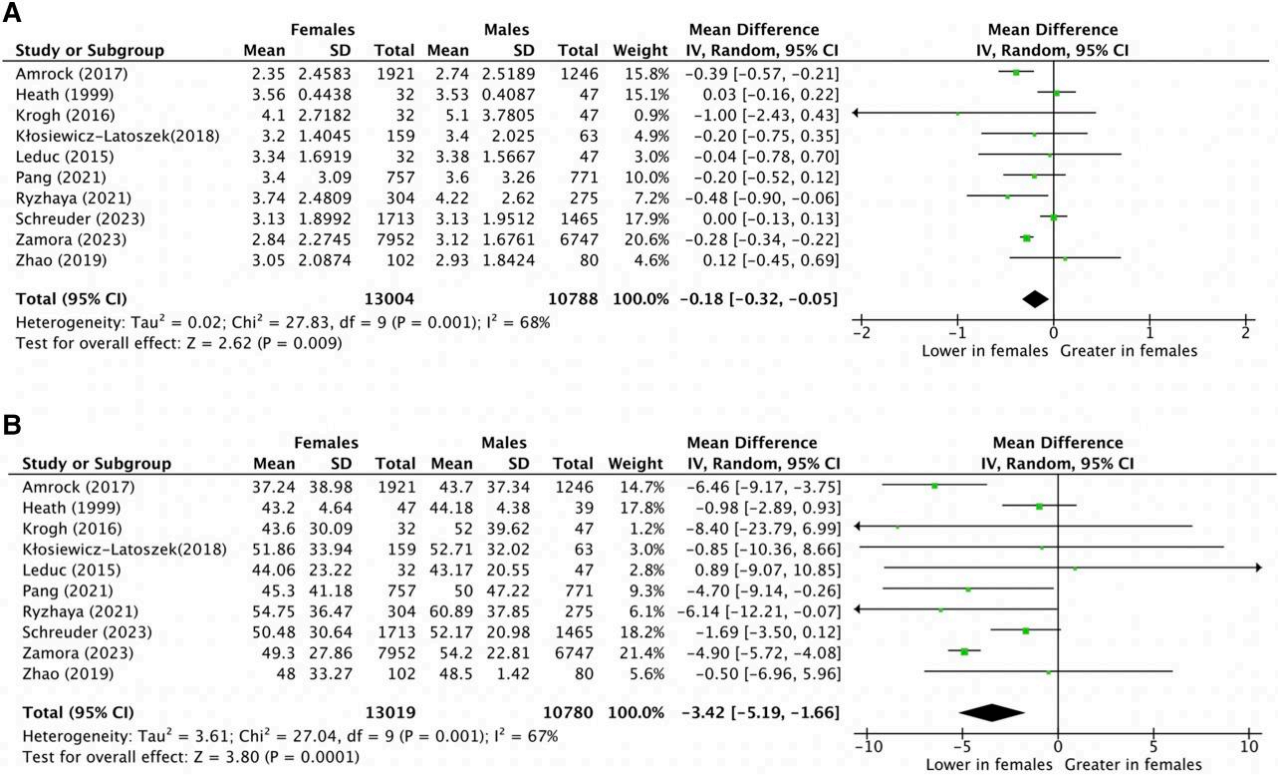
Sex differences in LDL cholesterol reductions in males and females in observational studies included in the systematic review of sex differences in the treatment of familial hypercholesterolaemia with lipid-lowering therapies. Panel (*A*) depicts sex differences in mean LDL cholesterol reduction (mmol/L) reported in observational studies. Panel (*B*) depicts sex differences in mean LDL cholesterol reduction (%) from baseline levels reported in observational studies. CI, confidence interval; SD, standard deviation

**Figure 5:**
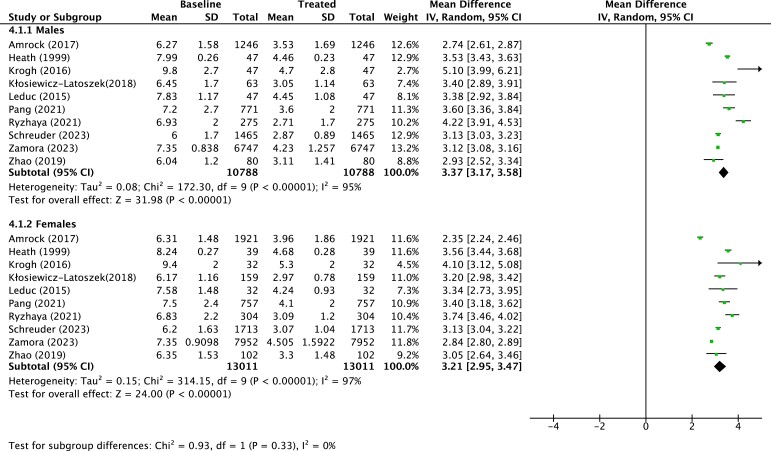
Absolute LDL cholesterol reductions (mmol/L) in males and females in observational studies included in the systematic review of sex differences in the treatment of familial hypercholesterolaemia. This figure depicts difference in means of LDL cholesterol from baseline to follow-up measurements reported in observational data. Squares represent mean differences; horizontal lines show 95% confidence intervals. Area of the square is proportional to the inverse variance of the estimate. Diamonds represent pooled estimates with 95% confidence intervals derived under the random-effects model. Solid vertical line indicates null effect. Test of subgroup differences refers to variations in the difference of means between male and female subgroups; *P*-values <.1 are considered significant. CI, confidence interval; SD, standard deviation

In the subsequent meta-analysis of real-world evidence data, observational studies with unavailable proportions of treated patients by sex, although implying sex differences in the treatment of FH, were excluded. Characteristics of patients from the remaining 25 observational studies are reported in *Table 1*.^6–8,10,11,20–40^ The majority of studies were published after the year 2016 (introduction of ICD-10 codes for FH) (*n* = 21). Excluding one large multi-national cohort,^6^ a total of 13 countries were represented in the sex differences in the treatment of FH meta-analyses, including Norway (*n* = 3), France (*n* = 3), Spain (*n* = 3), USA (*n* = 3), Canada (*n* = 2), and UK (*n* = 2), among others. A substantial number of studies comprised reports from national registries (*n* = 10), where ascertainment of FH was predominantly through a combination of clinical and genetic criteria (*Table 1*). Quality of included studies, as assessed by the EPHPP tool, was predominantly moderate or strong. Sex differences in treatment with LLT in observational studies are shown in *Figure 3*. Meta-analysis of data from the 25 studies (129 441 participants; 53.4% females) found that females with FH were less likely to be on LLT compared with males [OR .74 (95% CI .66–.85)], despite substantial heterogeneity (*I*^2^ = 90%). Age and previous history of ASCVD were not significantly different between males and females (data not shown). Mean LDL-C reductions in mmol/L and in percent change were compared between males and females as depicted in *Figure 4*. On average, LDL-C reductions inferred from baseline lipid values after treatment were greater in males than in females [mean difference in absolute LDL-C reduction of .18 mmol/L (.32–.05) mmol/L, and mean difference in percent LDL-C reduction of 3.42% (5.19–1.66)% greater in males vs. females] (*Figure 4*). This did not translate, however, in a statistical significant difference in absolute LDL-C reductions between sexes [−3.37 mmol/L (−3.17, −3.58) in males vs. −3.21 mmol/L (−2.95, −3.47) in females; *P* = .33] (*Figure 5*).

**Table 1:** Characteristics of the 25 real-world observational studies included in the meta-analysis of sex differences in the treatment of familial hypercholesterolaemia with lipid-lowering therapies

First author	Year	Country	Study design	Participants	Diagnosis criteria	Recruitment period	Males *n*	Females *n*	On LLT	LLT	Baseline LDL-C	Treated LDL-C	LDL-C % change
**Agarwala^20^**	2023	USA	Retrospective cohort	HeFH	DLCN SB MEDPED AHA Genetic	NR	280	502	Statins M: 84.0% F: 78.0%	Statins Ezetimibe PCSK9i	NR	M: 3.00 ± 1.29 F: 3.23 ± 1.24	NR
**Amrock^21^**	2017	USA	Cross-sectional analysis of registry data	HeFH HoFH	SB DLCN MEDPED	2014–16	1246	1921	Statins M: 74.6% F: 66.7%	Statins Ezetimibe Bile acid seq Niacin PCSK9i	M: 6.27 ± 1.58 F: 6.31 ± 1.48	M: 3.53 ± 1.69 F: 3.96 ± 1.86	M: −43.7% F: −37.2%
**Arnesen^22^**	2020	Norway	Retrospective cohort	HeFH	DLCN Genetic	2006	At last visit: 147	At last visit: 127	M: 96.6% F: 89.8%	Statins Ezetimibe Resins PCSK9i	NR	M: 2.8 (2.6–3.0) F: 3.3 (3.0–3.5)	NR
**Beliard^23^**	2014	France	Cross-sectional	HeFH	SB DLCN Genetic	1988–2011	826	843	M: 89.4% F: 79.1%	Statins Ezetimibe Bile acid seq Fibrate	NR	NR	NR
**Benn^10^**	2012	Denmark	Cross-sectional	HeFH	DLCN Genetic	1977–2011	204	298	M: 50.0% F: 47.0%	Statins Ezetimibe Bile acid seq other	NR	NR	NR
**Gallo^24^**	2017	France	Prospective	HeFH	Genetic	2015	56	56	M: 94.6% F: 69.6%	Statins Ezetimibe	NR	NR	NR
**Jackson^26^**	2021	USA	Retrospective cohort	FH	USA ICD code for FH	2016–19	25 367	28 431	Statins M: 65.8% F: 59.6%	Statins Ezetimibe PCSK9i, Fenofibrate Other	NR	NR	NR
**Jiménez^27^**	2023	Spain	Retrospective cohort—registry	HeFH	DLCN Genetic	NR	1583	1778	Statins: 84.1%	Statins PCSK9i	NR	NR	NR
**Korneva^28^**	2019	Russia	Retrospective cohort—registry	HeFH	DLCN	NR	75	116	Statins Overall: 65.0% M: 58.7% F: 69.0%	Statins	NR	NR	NR
**Krogh^29^**	2016	Norway	Retrospective cohort—registry	FH	DLCN Genetic	1989–2010	47 LLT data: 43	32 LLT data: 31	Statins: M: 93.0% F: 87.1%	Statins Ezetimibe Bile acid seq Niacin Other	M: 9.8 ± 2.7 F: 9.4 ± 2.0	M: 4.7 ± 2.8 F: 5.3 ± 2.0	M: −52.0% F: −43.6%
**Li^30^**	2017	China	Retrospective cohort	FH	DLCN Genetic	2011–16	162	119	CAD + M: 88.8% F: 78.6% CAD- M: 64.3% F: 57.1%	Statins	NR	NR	NR
**Matta^31^**	2021	Argentina	Prospective study	FH	DLCN	2015–20	30	85	Statins M: 26.7% F: 38.8%	Statins	NR	NR	NR
**Mattina^32^**	2019	France	Prospective study	FH	Genetic	2015–16	70	84	M: 92.9% F: 69.0%	Statins Ezetimibe	NR	NR	NR
**Mundal^11^**	2014	Norway	Registry	HeFH HoFH	Genetic	1992–2010	59 LLT data: 38	54 LLT data: 30	88.2%	Statins ± other LLT	NR	M: 4.4 ± 1.4 F: 5.0 ± 1.6	NR
**Neil^33^**	2004	UK	Cross-sectional study—registry	HeFH	SB	1980–96	211 CAD+:104 CAD−:107	199 CAD+: 55 CAD−:144	CAD + M: 99.0% F: 94.5% CAD− M: 92.5% F: 74.3%	Statins	NR	NR	NR
**Pang^34^**	2021	Australia	Registry	FH	DLCN Genetic	2015–19	771	757	LLT M: 84.3% F: 77.3%	Statins Ezetimibe PCSK9i	M: 7.2 ± 2.6 F: 7.5 ± 2.4	M: 3.6 ± 2.0 F: 4.1 ± 2.0	M: −50.0% F: −45.3%
**Pérez-Calahorra^35^**	2017	Spain	Cross-sectional analysis of registry data	HeFH	DLCN	2013–16	851	881	NR	NR	NR	NR	NR
**Ryzhaya^7^**	2021	Canada	Retrospective longitudinal study using registry data	FH	DLCN	NR	275	304	Statins M: 89.6% F: 88.4%	Statins Ezetimibe PCSK9i	M: 6.93 ± 2.0 F: 6.83 ± 2.2	M: 2.71 ± 1.7 F: 3.09 ± 1.2	M: −60.9% F: −54.8%
**Schreuder^36^**	2023	Netherlands Norway	Cross-sectional study	HeFH	DLCN Genetic	2011–17	1465	1713	Per type of LLT *Table 1*	Statins Ezetimibe PCSK9i	M: 6.0 ± 1.7 F: 6.2 ± 1.6	M: 2.8 ± .9 F: 3.1 ± 1.0	M: −52.2% F: −50.5%
**Vallejo-Vaz^37^**	2018	UK	Retrospective study	HeFH	Phenotypic	NR	714	626	M: 65.5% F: 65.5%	PCSK9i—Aliro 75/150mg	In mg/dL M: 150.8 ± 54.1 F: 159.6 ± 62.5	NR	NR
**Vallejo-Vaz^6^**	2021	International^a^	Retrospective cross-sectional—registry	HeFH	DLCN Genetic MEDPED SB Canadian JAS	NR	19 031	21 999	M: 61.1% F: 58.4%	Statins Ezetimibe Fibrates PCSK9i	NR	Median (IQR) M: 4.18 (3.16–5.51) F: 4.26 (3.24–5.75)	NR
**Vlad^38^**	2021	Romania	Prospective cohort	FH	SB DLCN MEDPED	2016–17	22	39	Statins monotherapy at registration M: 39.8% F: 38.5%	Statins Ezetimibe Fibrates	NR	NR	NR
**Waluś-Miarka^39^**	2017	Poland	Prospective cohort	FH	SB Genetic	2011–13	63	91	NR	NR	NR	NR	NR
**Zamora^8^**	2023	Spain	Cross-sectional	FH-phenotype	Phenotypic	2006–14	6747 CAD+: 1659 CAD−: 5088	7952 CAD+: 919 CAD−: 7033	Per type of statin *Table 2*	Statins Ezetimibe	Overall M: 7.35 ± .8 F: 7.35 ± .9 CAD + M: 7.44 ± .90 F: 7.44 ± .93 CAD− M: 7.31 ± .98 F: 7.34 ± .95	Overall M: 4.23 ± 1.3 F: 4.50 ± 1.6 CAD + M: 3.41 ± 1.24 F: 3.77 ± 1.29 CAD− M: 4.62 ± 1.5 F: 4.65 ± 1.6	Overall M: 42.4% F: 38.6% CAD + M: −54.2% F: −49.3% CAD− M: −36.8% F: −36.6%
**Zhao^40^**	2019	Canada	Bi-directional cohort	FH	Canadian Genetic	NR	80	102	M: 10.0% F: 6.9%	NR	M: 6.04 ± 1.2 F: 6.35 ± 1.53	M: 3.11 ± 1.41 F: 3.30 ± 1.48	M: −48.5% F: −48.0%

AHA, American Heart Association; Bile acid seq., bile acid sequestrants; CAD, coronary artery disease; DLCN, Dutch Lipid Clinic Network; F, females; FH, familial hypercholesterolaemia; HeFH, heterozygous familial hypercholesterolaemia; HoFH, homozygous familial hypercholesterolaemia; ICD code, International Classification of Diseases; IQR, interquartile range; JAS, Japanese Atherosclerosis Society; LDL-C, low-density lipoprotein cholesterol; LLT, lipid-lowering therapy; M, males; MEDPED, Making Early Diagnosis to Prevent Early Deaths; mg, milligrams; NR, not reported; PCSK9i, proprotein convertase subtilisin/kexin type 9 inhibitor; SB, Simon Broome; UK, United Kingdom; USA, United States of America.

Values are mean ± standard and units in mmol/L unless otherwise stated.

Heath *et al.*^25^: Detailed data on treatment of FH per sex in the full group were not available, but data on LDL-C measurements in a subgroup of 47 males vs. 39 females with tendon xanthomas were included in the analyses shown in *Figures 4* and *5*.

^a^56 countries (of 66) participating in the European Atherosclerosis Society's Familial Hypercholesterolaemia Studies Collaboration.

Country-specific estimates of sex differences in the treatment of FH with LLT showed heterogeneity in data, whereby in a majority of the 13 countries represented, with the exception of Argentina, Poland, Romania, and Russia, females were less likely to be treated than males (see Supplementary data online, *Table S2* and *Figure S3*). However, in subgroup analyses of sex differences in treatment with LLT by WHO geographical location demonstrated that in all regions (Americas, Europe, Western Pacific, and International), females with FH were less likely to be on LLT compared with males with FH (see Supplementary data online, *Figure S4*). They were also less likely to be treated compared with males in studies where a majority of participants (>50%) were diagnosed using genetic testing vs. phenotypical/clinical diagnosis (see Supplementary data online, *Figure S5*). A subgroup analysis of sex differences in treatment was further performed by year of publication of studies included, using year 2016 as a cut-point. There were no significant sex disparities between pooled results obtained before and after 2016 (*P* = .06, Supplementary data online, *Figure S6*). Similar findings were obtained when stratifying by study sample size, with fewer vs. more than 1000 patients used as a cut-point (*P* = .23, Supplementary data online, *Figure S7*).

The impact of various types and doses of LLTs between sexes was investigated next (summary estimates in Supplementary data online, *Table S3*). Using random-effects estimates, comparable trends were observed for all medication classes and intensity, with females with FH less likely to be treated with statins [OR .79 (.69–.92)], particularly high-intensity statins [OR .66 (.57–.76)], ezetimibe [OR .67 (.57–.78)], statins and ezetimibe [OR .64 (.48–.86)], PCSK9 inhibitors [OR .70 (.54–.91)], and two or more LLTs [OR .67 (.53–.84)] (see Supplementary data online, *Table S4* and *Figures S8*–*S11*). This observed trend seemed to diminish, however, with year of publication (see Supplementary data online, *Figure S12*).

**Figure 6:**
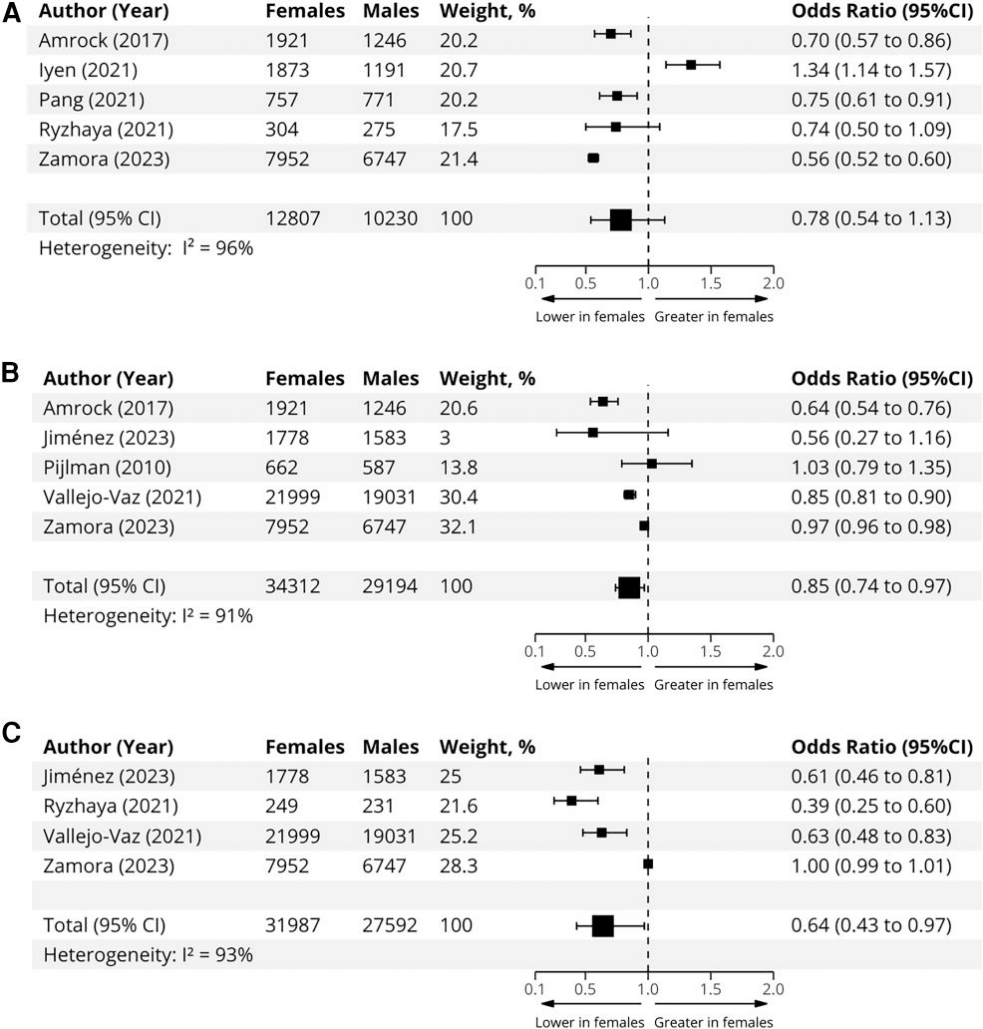
Meta-analyses of sex differences in LDL cholesterol reduction target attainment. Panel (*A*) depicts sex differences in attainment of ≥50% reductions in LDL cholesterol. Panel (*B*) depicts sex differences in attainment of an LDL <2.5 mmol/L. Panel (*C*) depicts sex differences in attainment of an LDL <1.8 mmol/L. Small squares indicate study-level estimates of sex differences in treatment (odds ratios); large squares represent pooled odds ratio derived under random-effects models; horizontal lines represent 95% confidence intervals; vertical dashed line represents null effect. CI, confidence interval

In achievement of guideline-recommended lipid targets or thresholds, females were also less likely to reach ≥50% reduction in LDL-C from baseline [OR .78 (.54–1.13)], an LDL-C < 2.5 mmol/L [OR .85 (.74–.97)], or an LDL-C < 1.8 mmol/L [OR .64, (.43–.97)] (*Figure 6* and Supplementary data online, *Table S4*).

**Figure 7:**
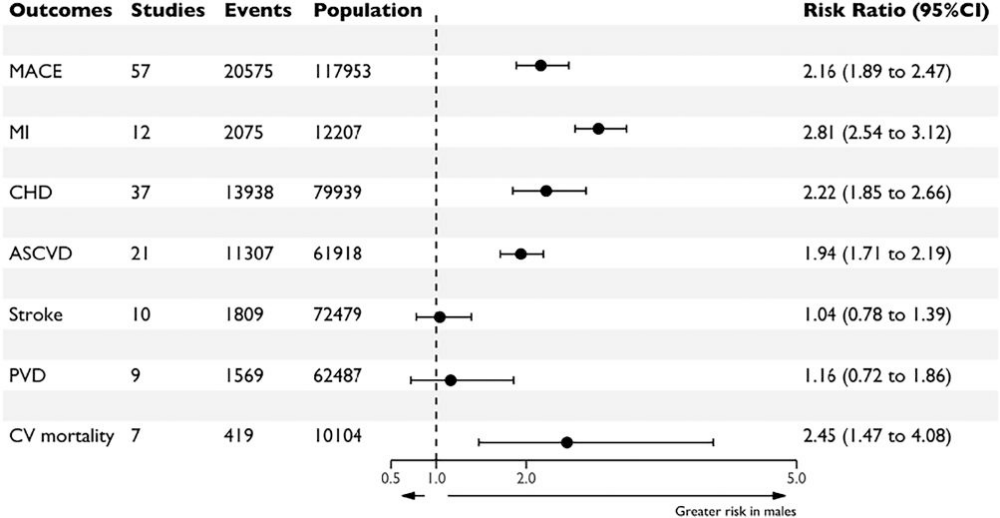
Risk of major adverse cardiovascular events in males vs. females with familial hypercholesterolaemia. This figure depicts pooled estimates (circles) with 95% confidence intervals (horizontal lines) for comparisons of the risk of major adverse cardiovascular events in males vs. females with familial hypercholesterolaemia. All pooled estimates are derived using inverse-variance weighting incorporating random-effects. ASCVD, atherosclerotic cardiovascular diseases; CHD, coronary heart disease; CI, confidence interval; CV, cardiovascular; MACE, major adverse cardiovascular events; MI, myocardial infarction; PVD, peripheral vascular disease

From all 133 studies included in this systematic review of sex differences in the treatment of FH, 57 studies reported data on CVD outcomes and were included in a meta-analysis of MACE. Characteristics of patients from these studies (117 953 participants) are shown in *Table 2*.^6,10,20,30,35,38,40–90^ Studies followed participants from a range of 12 weeks to 15 years. Pooling these studies (*Figure 7*) with 20 575 events, males with FH were identified as having an upward of two-fold greater relative risk of MACE compared with females (OR 2.16 [1.89–2.47]) and a significantly stronger risk of myocardial infarction (MI) [OR 2.81 (2.54–3.12)], with little heterogeneity between studies (*I*^2^ = 0%, *P* = .76). Males also had greater relative risk of coronary heart disease [OR 2.22 (1.85–2.66)], ASCVD [OR 1.94 (1.71–2.19)], and cardiovascular mortality [OR 2.45 (1.47–4.08)]. There were no differences in risk of stroke or peripheral vascular disease between males and females, in 10 studies (72 479 participants; 1809 events) for stroke and nine studies (62 487 participants; 1569 events) for peripheral vascular disease.

**Table 2:** Characteristics of the 57 studies included in the meta-analysis of sex differences in major adverse cardiovascular events in risk of familial hypercholesterolaemia

First author	Year	Country	Study design	Participants	Diagnosis criteria	Recruitment period	Males, *n*	Females, *n*	CVD outcomes	Follow-up time
**Agarwala^20^**	2023	USA	Retrospective cohort	HeFH	DLCN SB MEDPED AHA Genetic	NR	280	502	Premature ASCVD	NR
**Ahmad^41^**	2016	USA	Retrospective cohort	HeFH	Genetic	NR	42	51	Premature CHD	NR
**Allard^42^**	2014	Canada	Retrospective cohort	HeFH	DLCN	1970–2014	180	229	CVD	NR
**Alonso^43^**	2014	Spain	Registry	HeFH	Genetic	NR	921	1039	CVD	NR
**Beaumont^44^**	1976	France	Cross-sectional	FH	Phenotypic	NR	158	116	IVD: Angina, MI, PVD	NR
**Benn^10^**	2012	Denmark	Cross-sectional	HeFH	DLCN Genetic	1977–2011	204	298	CAD	NR
**Berard^45^**	2019	France	Retrospective cohort	HeFH	DLCN	1995–2005	35	32	Premature ASCVD	NR
**Bertolini^46^**	2013	Italy	Retrospective cohort	HeFH HoFH	DLCN Genetic	NR	818	951	CHD	NR
**Besseling^47^**	2014	Netherlands	Registry	HeFH	Genetic	1994–2013	6848	7435	CVD	NR
**Bhatnagar^48^**	2000	UK	Retrospective cohort	HeFH	SB	1987–98	183	197	Angina, MI, CABG, Stroke, CHD, CVD	NR
**Bogsrud^49^**	2019	Norway	Registry	HeFH	Genetic	2014–15	307	407	MI, CHD	11.1 ± 7.9 years
**Bowden^50^**	1994	Canada	Retrospective cohort	HeFH	Phenotypic	NR	48	67	CAD	NR
**Carmena^51^**	1996	Canada	Retrospective cohort	HeFH	Phenotypic	NR	45	53	ASCVD	NR
**Chan^52^**	2015	Australia	Cross-sectional	HeFH	Phenotypic Genetic	2007–14	171	219	CAD	NR
**De Sauvage Nolting^53^**	2003	Netherlands	Cross-sectional	HeFH	DLCN Genetic	NR	287	229	CVD	NR
**Doi^54^**	2021	Japan	Retrospective cohort	HeFH	Genetic JAS	2005–16	116	116	MI, revascularization	NR
**Duell^55^**	2019	USA	Registry	HeFH	DLCN Genetic MEDPED SB	NR	744	1156	ASCVD	20 ± 11 months
**Ershova^56^**	2017	Russia	Retrospective cohort	HeFH	DLCN	2012–13	7	23	CAD, MI	NR
**Firth^57^**	2008	South Africa	Retrospective cohort	HeFH	Phenotypic Genetic	NR	488	581	Angina, MI, IHD, Stroke, TIA, PVD, Death	NR
**Hill^58^**	1990	Canada	Cross-sectional	HeFH	Phenotypic	NR	CAD data: 115	CAD data: 173	Angina, CAD, MI, Stroke	NR
**Hirobe^59^**	1982	Japan	Cross-sectional	HeFH	Phenotypic	NR	30	22	CAD	NR
**Holmes^60^**	2005	Canada	Retrospective cohort	HeFH	SB	NR	173	215	CVD	NR
**Hoogerbrugge^61^**	1999	Netherlands	Clinical trial	HeFH	Phenotypic	NR	20	20	CAD	12 weeks
**Hopkins^62^**	2001	USA	Registry	HeFH	MEDPED	NR	112	150	Premature CAD	NR
**Iyen^63^**	2019	UK	Retrospective cohorty—registry	FH	DLCN SB	1999–2016	6578	7519	CVD	13.8 (8.4–17.7) years
**Jansen^64^**	2004	Netherlands	Retrospective cohorty—registry	HeFH	DLCN MEDPED SB Genetic	1989–99	1179	1221	CVD	CVD+: 4.7 (2.4–9.0) years CVD−: 3.2 (1.2–6.5) years
**Khoury^65^**	2021	Canada	Bi-directional cohort	FH	SB Genetic	NR	891	888	CVE	NR
**Li^30^**	2017	China	Retrospective cohort	FH	DLCN Genetic	2011–16	162	119	CAD, Premature CAD	NR
**Mabuchi^66^**	1977	Japan	Cohort	HeFH	Phenotypic	NR	IHD data: 37	IHD data: 46	IHD	NR
**Michikura^67^**	2017	Japan	Cross-sectional	HeFH	Phenotypic	2013–16	53	77	CAD	NR
**Miettinen^68^**	1988	Finland	Retrospective cohort	HeFH	Phenotypic	1968–70	48	48	CAD, CAD Mortality	15 years
**Miname^69^**	2019	Brazil	Prospective study	HeFH	Genetic	NR	75	131	MACE	Median (IQR) 3.7 (2.7–6.8) years
**Mohrschladt^70^**	2004	Netherlands	Retrospective cohort	FH	Phenotypic	NR	190	210	CVD	8 years
**Mundal^71^**	2016	Norway	Registry	HeFH HoFH	Genetic	1994–2009	2693	2845	CVD hospitalizations	Median (IQR) 5 (1–9) years
**Neil^72^**	2008	UK	Cross-sectional study—registry	HeFH	SB	1980–2006	1650	1732	Angina, CHD, CVD mortality, MI	Median M: 14.5 years F: 14.1 years
**Nenseter^73^**	2011	Norway	Retrospective cohort	HeFH	Genetic	2007–9	68	44	CHD	NR
**Panagiotakos^74^**	2003	Greece	Prospective cohort	HeFH	MEDPED	1987–97	295	344	CHD	15 years
**Pang^75^**	2018	South Africa Australia Brazil	Retrospective cohort—registry	HeFH	Genetic	1990–2017	399	476	CAD	NR
**Perak^76^**	2016	USA	Retrospective cohort	HeFH	AHA	1999–2010	1559	2291	ASCVD, CHD	≥10 years
**Pérez-Calahorra^35^**	2017	Spain	Cross-sectional analysis of registry data	HeFH	DLCN	2013–16	851	881	CVD	NR
**Perez de Isla^77^**	2017	Spain	Registry	HeFH	Genetic	2004–15	1087	1317	ASCVD	5.5 ± 3.2 years
**Perez Garcia^78^**	2018	Spain	Retrospective cohort	HeFH HoFH	Genetic	2001–17	67	66	CHD	NR
**Pisciotta^79^**	2005	Italy	Prospective cohort	HeFH	Phenotypic	NR	103	146	CAD	NR
**Pitsavos^80^**	2004	Greece USA	Retrospective cohort	HeFH	Phenotypic	1987–2002	295	344	CHD	6 ± 3 years
**Ramos^81^**	2020	Spain	Retrospective cohort	FH-phenotype	Phenotypic	2006–13	3047	4385	ASCVD	NR
**Sánchez-Ramos^82^**	2021	Spain	Prospective cohort	HeFH	Phenotypic	2004–7	602	105	MACE	6.6 ± 3.6 years
**Seed^83^**	1990	UK	Retrospective cohort	HeFH	SB	NR	61	54	CHD	12 months
**Silva^84^**	2016	Brazil	Prospective cohort	FH	Genetic	NR	302	516	CVD	1 year
**Simonen^85^**	1987	Finland	Retrospective cohort	HeFH	Phenotypic	1970s	49	48	Angina, CAD	NR
**Slack^86^**	1969	UK	Retrospective cohort	HeFH	Phenotypic	NR	51	53	IHD, IHD mortality	NR
**Tada^87^**	2023	Japan	Retrospective cohort	HeFH HoFH	JAS Genetic	2000–20	490	560	MACE	12.6 (9.1–17.4) years
**Vallejo-Vaz^6^**	2021	International**^a^**	Retrospective cross-sectional—registry	HeFH	DLCN Genetic MEDPED SB Canadian JAS	NR	19 031	21 999	CAD, PAD, Premature CAD, Stroke	NR
**Vlad^38^**	2021	Romania	Prospective cohort	FH	SB DLCN MEDPED	2016–17	CHD data in 61	CHD data in 61	ASCVD, CHD, PAD, Stroke	2 years
**Vuorio^88^**	1997	Finland	Registry	HeFH	Phenotypic Genetic	1992–96	73	106	CHD, MI	NR
**Wierzbicki^89^**	2000	UK	Retrospective cohort	HeFH	SB	NR	66	46	CHD	≥6 years
**Yaman^90^**	2020	Turkey	Cross-sectional	HeFH	DLCN	2010–16	119	248	CHD	NR
**Zhao^40^**	2019	Canada	Bi-directional cohort	FH	Canadian Genetic	NR	80	102	Premature MI	≤1 year

AHA, American Heart Association; ASCVD, atherosclerotic cardiovascular disease; CABG, coronary artery bypass graft surgery; CAD, coronary artery disease; CHD, coronary heart disease; CVD, cardiovascular disease; CVE, cardiovascular event; DLCN, Dutch Lipid Clinic Network; F, females; FH, familial hypercholesterolaemia; FU, follow-up; HeFH, heterozygous familial hypercholesterolaemia; HoFH, homozygous familial hypercholesterolaemia; IHD, ischemic heart disease; IQR, interquartile range; IVD, ischaemic vascular disease; JAS, Japanese Atherosclerosis Society; LDL-C, low-density lipoprotein cholesterol; M, males; MACE, major adverse cardiac events; MEDPED, Making Early Diagnosis to Prevent Early Deaths; MI, myocardial infarction; NR, not reported; PAD, peripheral arterial disease; PVD, peripheral vascular disease; SB, Simon Broome; UK, United Kingdom; USA, United States of America.

From the 133 studies included in the qualitative synthesis, i.e. in studies that were found to have data on sex differences in the treatment of FH, 57 were found to have quantitative data on the risk of MACE outcomes for meta-analysis.

^a^56 countries (of 66) participating in the European Atherosclerosis Society’s Familial Hypercholesterolaemia Studies Collaboration.

## Discussion

In the present study, important sex disparities in treatment and lipid target achievement in patients with FH were observed and should be taken into consideration. With data in more than 129 000 patients, this is the largest systematic review performed to date providing evidence for sex differences in treatment with LLT among individuals with FH. These results emphasize the importance of considering sex in risk-stratifying patients with FH and highlight the need for sex-specific strategies for CVD prevention.

In clinical trials using fixed doses of LLTs, males and females with FH displayed similar response to LDL-C lowering medications. Despite this, in observational studies, females were treated less intensively and were less likely to reach guideline-recommended LDL-C targets (see *Structured Graphical Abstract*). This was independent of WHO geographical location and the proportion of females studied, although the observed trend seemed to diminish with year of publication, suggesting that initiatives by national registries as well as international organizations such as the Family Heart FH Foundation and the FHSC led by the European Atherosclerosis Society may be having an impact to lessen these sex disparities.^91,92^ Further research is nevertheless needed to identify causes underlying these disparities.

The reasons behind these sex differences are not fully understood but are likely multifactorial. In terms of direct care, one possibility could be that females are reluctant to be treated with LLTs or under-estimate their own health risk with FH. However, our group has previously shown that females do not appear to minimize this risk associated with FH or CVD.^5^ Other reasons include adverse effects. It has been well described that in general, females report a significantly higher number of side events with LLT than males which may impede up-titration to optimal LLT.^36^ Healthcare providers might also play a role. In a nationwide multicentre Spanish registry with 3361 adult patients with FH, females had a 49% lower chance of being prescribed a PCSK9 inhibitor than males.^27^ However, prior studies on FH report no sex differences in adherence to LLT.^93,94^

In FH, LLT is recommended to reduce the risk of ASCVD without differences according to sex. Evidence from clinical trials of LLT in patients with FH indicates that statins are equally effective in both males and females in the prevention of ASCVD in high-risk populations. In the present meta-analysis, however, we confirm that males and females with FH are less likely to reach guideline-mandated therapeutic thresholds for primary and secondary prevention, with females being treated less intensively than males. These findings support recent studies where females received less high-potency statins and fewer females reached lipid targets of LDL <2.0 mmol/L.^7^ This lower intensity LLT was especially evident for females in secondary prevention. The differences in goal achievement can be partly explained by the finding that females with FH have higher LDL-C levels from an earlier age,^95^ are diagnosed 3–7 years later than males, and seldom use maximally tolerated statin doses or combination LLT.^6^ As a result, achievement of recommended LDL-C treatment goals is subsequently lower. These disparities in FH care impact ASCVD risk, with registry data showing the highest excess risk among younger females with FH.^11,96^

In the present study, even though females with FH were treated less intensively and reached their LDL-C goals less frequently, males had more than two-fold greater cardiovascular risk. This disparity was consistent across various subgroups and outcomes, including MI, ASCVDs, and cardiovascular death. The association between FH and ASCVD is widely recognized, but there has been uncertainty regarding equality of this excess risk in males and females. While an early report from the Copenhagen General Population Study found no meaningful difference in risk estimates between sexes,^10^ the UK Simon Broome and Norwegian registries have since documented greater cardiac morbidity and mortality among females.^11,96^ More recently, a multi-national cross-sectional study of FH registries demonstrated a greater risk of prevalent CAD in males.^6^ Part of the uncertainty in the evidence may be attributed to disharmony in outcomes examined by previous individual studies and the referral bias seen in disease-specific registries compared with general population settings. Further, females are generally underrepresented in FH and CVD literature, resulting in a lack of statistical precision in risk estimates. Finally, absence of direct comparisons between males and females with FH has made interpreting the limited available data challenging. In this meta-analysis, we aimed to address these shortfalls in the literature.

The sex differences reported here potentially reflect a culmination of genetic and hormonal factors, sex-specific health behaviours, and some systemic determinants. For example, our findings might suggest that other cardio-protective factors, such as pre-menopausal status, higher HDL cholesterol levels, lower prevalence of other cardiovascular risk factors, such as tobacco, or higher levels of triglycerides and remnant lipoprotein cholesterol in males might play a role. In fact, males in the present study may have had more cardiovascular risk factors than their female counterparts,^38^ which have been shown to exert cumulative^97^ and sex-specific impacts on CVD risk among those with FH. While some risk estimates included in our analyses accounted for these factors, it is likely that some were not fully adjusted for. Excess risk observed in males may have also been due to differences in treatment with LLT, as we were not able to account for treatment intensity, efficacy, or duration in our analyses. This explanation may be less likely, however, given our current results suggesting that males with FH are treated earlier^98^ and more aggressively than female counterparts and are more likely to reach cholesterol reduction targets.^27^ Finally, it may be possible that a greater proportion of females included in studies represented non-index cases given the earlier onset of cardiovascular events in males. Studies have demonstrated that affected relatives are detected several years earlier, with fewer cardiovascular risk factors and improved cardiovascular outcomes.^6^

Interestingly, no difference between males and females was found in the risk of stroke and peripheral vascular disease, contrasting patterns seen in the general population.^99–101^ A potential explanation for this may be similar rates of predisposing factors such as atrial fibrillation and heart failure among males and females with FH.^102^ Alternatively, it is possible that factors such as age, socioeconomic status, and lifestyle behaviours interact with sex and gender to impact the likelihood of stroke and peripheral vascular disease.^103^ If that were the case, uncovering these potentially protective determinants would present an important priority for future research.

This study has some strengths and limitations that merit consideration. Among its strengths are its exhaustive search, large sample size, diversity of study populations, extensive sensitivity investigations, and the important information it brings to the field. In terms of limitations, first omission of relevant reports cannot be ruled out despite extensive search efforts. However, the large number of studies included in our primary analysis made these results robust to the inclusion of any single investigation. Second, studies reporting significant associations between sex and cardiovascular outcomes might be more likely to be published. Third, it is recognized that the trim-and-fill method may not be valid in the presence of excess heterogeneity between studies.^19^ A high degree of heterogeneity (*I*^2^ > 70%) was observed for several analyses, likely explained by difference in studies design, diagnostic criteria, and endpoint definitions, suggesting bias between studies (see Supplementary data online, *Figure S13*). This is consistent with the meta-analysis being a study-level rather than a patient-level meta-analysis, with both retrospective and prospective studies included. We anticipated and accounted for this heterogeneity using random-effects models. Finally, while our study evaluated sex differences in outcomes in patients with FH, we were unable to account for gender identity and other important aspects of intersectionality in our analyses. Accordingly, these present pressing areas for future research.

## Conclusions

The present study found than males and females with FH show similar response to LDL-C lowering medications. Despite this, females seemed less likely to be treated intensively and to reach guideline-recommended LDL-C targets. A better understanding of drivers of sex-related disparities in FH treatment is needed. Identifying these imbalances will allow us to reduce barriers to care and improve survival in individuals with FH.

## Supplementary data

Supplementary data are available at *European Heart Journal* online.

## Supplementary Material

ehae417_Supplementary_Data

## Data Availability

The Rayyan file containing the studies reviewed and included in the systematic review is available from the corresponding author on reasonable request.
