# Supplementary material for: Sex differences in treatment of familial hypercholesterolaemia: a meta-analysis
Source: Eur Heart J. 2024 Jul 8;45(35):3231–50. doi: 10.1093/eurheartj/ehae417 (PMC11400737; doi:10.1093/eurheartj/ehae417)
Supplement: ehae417_Supplementary_Data [file ehae417_supplementary_data.pdf]

SUPPLEMENTARY APPENDICES

SEX DIFFERENCES IN THE TREATMENT OF FAMILIAL HYPERCHOLESTEROLEMIA: A SYSTEMATIC REVIEW AND META-ANALYSIS

Iulia Iatan*, Leo E. Akioyamen*, Isabelle Ruel, Amanda Guerin, Lindsay Hales, Thais Coutinho, Liam R. Brunham, Jacques Genest

These authors contributed equally to this work and share first authorship

Appendix 1: Supplementary Methods including:

1. Original search strategy
2. Supplementary Methods
3. Supplementary References

Appendix 2: Supplementary Tables and Figures

Appendix 3: Quality assessment of studies included in systematic review.

Appendix 4: Reference list of the 133 studies included in the qualitative synthesis from the systematic review of sex differences in the treatment of familial hypercholesterolemia.

Appendix 1.1. Search strategy.

Original search for Ovid MEDLINE(R) and Epub Ahead of Print, In-Process, In-Data-Review & Other Non-Indexed Citations and Daily <1946 to July 21, 2020>

#	Searches	Results
1	Hyperlipoproteinemia Type II/	6707
2	Hypercholesterolemia/ or Hyperlipidemia, Familial Combined/ or Hyperlipidemias/ge [Genetics]	28853
3	limit 2 to yr="1966 - 1979"	3659
4	((familia* or type* 2 or type* 2s or type* ii or type iis or type* iia* or type* iib* or essential* or autosomal dominant or genetic*) adj3 (hypercholesterolemi* or hypercholesterolaemi* or hyperlipoproteinemi* or hyperlipoproteinaemi* or hyper-cholesterolemi* or hyper-cholesterolaemi* or hyper-lipoproteinemi* or hyper-lipoproteinaemi* or apolipoprotein-b* or dyslipidemi*)).tw,kf.	10219
5	(HoFH or HFH or HzFH or HeFH or HHF).tw,kf.	1057
6	((extreme* or rare* or severe* or homozyg* or homo-zygo* or heterozygo* or hetero-zygo*) adj3 (hypercholesterolemi* or hypercholesterolaemi* or hyperlipoproteinemi* or hyperlipoproteinaemi* or hyper-cholesterolemi* or hyper-cholesterolaemi* or hyper-lipoproteinemi* or hyper-lipoproteinaemi* or apolipoprotein-b*)).tw,kf.	3249
7	(hyperbetalipoproteinemi* or hyperbetalipoproteinaemi* or hyper-beta-lipoproteinemi* or hyper-beta-lipoproteinaemi* or ((lipoproteinemi* or lipoproteinaemi*) adj3 (hyper-low* or hyper-beta* or hyperlow* or hyperbeta*)) or ldl receptor disorder*).tw,kf.	154
8	lipoid gout*.tw,kf.	4
9	(tendon* adj2 (xanthoma* or xanthogranulomatos*)).tw,kf.	404
10	((heterozygo* or hetero-zygo*) adj2 FH).tw,kf.	607
11	1 or 2 or 3 or 4 or 5 or 6 or 7 or 8 or 9 or 10	39819
12	Sex Characteristics/	54164
13	Sex/	7649
14	Sex ratio/	9234
15	Sex Factors/	263493
16	((sex* or gender* or man or men or male* or woman or women or female*) adj3 (difference* or different or characteristic* or ratio* or factor* or imbalanc* or issue* or both or specific* or disparit* or dependen* or gap or gaps or influenc* or discrepan* or distribut* or composition* or variability or comparison* or accept* or barrier* or perception* or perceiv* or between* or treat* or alirocumab or evolocumab or statin or atorvastatin or rosuvastatin or simvastatin or ezetimibe or ezetimib or niacin or enduracin or nicamin or nicobid or nicocap or nicolar or nicotinate or nicotinic or bile acid sequestrant or bempedoic acid or lomitapide or mipomersen or apheresis or PCSK9 inhibitor or anticholesteremic* or hypocholesteremic* or hmg-coa or hydroxymethylglutaryl or hydroxymethylglutaryl-coa or hydroxymethylglutaryl-coenzyme or (cholesterol adj2 inhibitor*))).tw,kf.	606145
17	((men or men's) adj2 women*).tw,kf.	127692
18	(gender*-related or gender*-based).tw,kf.	8281

19	or/12-18	874971
20	11 and 19	3040

Original search for Embase (Ovid)

#	Searches	Results
1	familial hypercholesterolemia/	9624
2	((familia* or type* 2 or type* 2s or type* ii or type iis or type* iia* or type* iib* or essential* or autosomal dominant or genetic*) adj3 (hypercholesterolemi* or hypercholesterolaemi* or hyperlipoproteinemi* or hyperlipoproteinaemi* or hyper-cholesterolemi* or hyper-cholesterolaemi* or hyper-lipoproteinemi* or hyper-lipoproteinaemi* or apolipoprotein-b* or dyslipidemi*)).tw,kw.	14268
3	(HoFH or HFH or HzFH or HeFH or HHF).tw,kw.	1943
4	((extreme* or rare* or severe* or homozyg* or homo-zygo* or heterozygo* or hetero-zygo*) adj3 (hypercholesterolemi* or hypercholesterolaemi* or hyperlipoproteinemi* or hyperlipoproteinaemi* or hyper-cholesterolemi* or hyper-cholesterolaemi* or hyper-lipoproteinemi* or hyper-lipoproteinaemi* or apolipoprotein-b*)).tw,kw.	4286
5	lipoid gout*.tw,kw.	0
6	(tendon* adj2 (xanthoma* or xanthogranulomatos*)).tw,kw.	604
7	((heterozygo* or hetero-zygo*) adj2 FH).tw,kw.	865
8	1 or 2 or 3 or 4 or 5 or 6 or 7	18974
9	exp sexual characteristics/	1706
10	sex/ or sex differentiation/	43123
11	sex ratio/	70111
12	sex factor/	7412
13	((sex* or gender* or man or men or male* or woman or women or female*) adj3 (difference* or different or characteristic* or ratio* or factor* or imbalanc* or issue* or both or specific* or disparit* or dependen* or gap or gaps or influenc* or discrepan* or distribut* or composition* or variability or comparison* or accept* or barrier* or perception* or perceiv* or between* or treat* or alirocumab or evolocumab or statin or atorvastatin or rosuvastatin or simvastatin or ezetimibe or ezetimib or niacin or enduracin or nicamin or nicobid or nicocap or nicolar or nicotinate or nicotinic or bile acid sequestrant or bempedoic acid or lomitapide or mipomersen or apheresis or PCSK9	832945

	inhibitor or anticholesteremic* or hypocholesteremic* or hmg-coa or hydroxymethylglutaryl or hydroxymethylglutaryl-coa or hydroxymethylglutaryl-coenzyme or (cholesterol adj2 inhibitor*))).tw,kw.	
14	((men or men's) adj2 women*).tw,kw.	172190
15	(gender*-related or gender*-based).tw,kw.	10902
16	9 or 10 or 11 or 12 or 13 or 14 or 15	1000931
17	8 and 16	994
18	("31652114" or "32536129" or "32405159" or "32284160" or "31914797" or "32299761" or "32671484" or "32128483" or "30563368" or "31840162" or "31674218" or "31235401" or "32576364" or "32592555" or "32616509" or "32655404" or "31868931" or "30549443" or "30957178" or "30974472" or "30567480" or "31877157" or "30986362" or "31280039" or "31153370" or "31870448" or "31696945" or "30683281" or "31878353" or "31523650" or "31733217" or "31048275" or "30953107" or "30573307" or "31336553" or "31380502" or "30609272" or "31617858" or "31617858" or "30649025" or "31378630" or "31758975" or "30993573" or "31307727" or "31847331" or "31596376" or "31618540" or "31708406" or "32237625" or "31133496" or "31092766" or "30129670" or "31409451" or "31003151" or "31818452" or "30380048" or "31860991" or "29569117" or "29852873" or "31483296" or "30652328" or "31196897" or "31171318" or "30442422" or "30934611" or "30794474" or "30653535" or "30937890" or "30004840" or "30786876" or "32245299" or "31293133" or "29124388" or "30755550" or "31197927" or "30720747" or "31555747" or "31229021" or "30640363" or "30460370" or "31006811" or "30753598" or "31130525" or "31130525" or "30732662" or "30527766" or "30158971" or "29459263" or "30172432" or "29863817" or "29500790" or "29149237" or "29973570" or "30253291" or "30371190" or "29137927" or "29795368" or "30433876" or "29447778" or "30055622" or "29969922" or "30374235" or "30156607" or "29321515" or "27738812" or "30055758" or "29566018" or "29576254" or "29178257" or "29980385" or "29407882" or "30260983" or "30270087" or "29789037" or "29622598" or "30039844" or "30203672" or "30021394" or "29983553" or "29458704" or "29788966" or "29534385" or "29630642" or "29315217" or "30270070" or "30276217" or "29703506" or "29673349" or "29174032" or "29331793" or "30259501" or "29154680" or "29512172" or "30026278" or "29716848" or "29751283" or "30580708" or "29609857" or "30012291" or "30270058" or "29800656" or "29461386" or "29321397" or "30005909" or "29386415" or "29453308" or "29402233" or "29726288" or "29377473" or "29247152" or "29459468" or "30007775" or "29791657" or "30625075" or "29229197" or "29412322" or "29871648" or "28600126" or "27794107" or "28826564" or "30375557" or "26686841" or "28663044" or "28685504" or "28637586" or "29109861" or "28321063" or "28878106" or "28153993" or "29264879" or "29473516" or "28480675" or "27822850" or "27920219" or "28946037" or "28156256" or "29286319" or "28762908" or "29169169" or "28595498" or "28081939" or "28941610" or "28990939" or "28532484" or "27811231" or "28559401" or "28652530" or "28225859" or "28158877" or "28038989" or "28356271" or "28419274" or "28062275" or "28461020" or "28906356" or "27932355" or "27923207" or "28110940" or "28458923" or "28882818" or "28515188" or "28078997" or "28738470" or "28460769" or "28379035" or "29146640" or "28992466" or "29050004" or "27885059" or "28825891" or "28457799" or "28759039" or "28430795" or "28241992" or "28499375" or "27853041" or "28376458" or "28314542" or "28445539" or "27426616" or "28526006" or "28776358" or "28699974" or "28434814" or "28615375" or "28623742" or "28697983" or "28554703" or	233

	"29080546" or "27697065" or "28449836" or "28532431" or "26896267" or "27556703" or "27655594" or "26539650" or "27916084" or "27208892" or "26970227" or "26555221" or "27294152" or "26875131" or "26670865" or "27048383" or "26957235" or "26302681" or "26999119" or "27538393" or "27100157" or "26589821" or "26889924" or "26506085" or "26806045" or "27062405" or "27019267" or "27444203" or "26521021" or "27452484" or "27119267" or "27664850" or "27558979" or "27127013" or "27125949" or "26387811").pm.	
19	("27486898" or "27559550" or "27532919" or "27559556" or "27206944" or "27307718" or "26466344" or "27678432" or "26586781" or "26810159" or "27678436" or "27595674" or "29537209" or "26807712" or "26932978" or "27225142" or "27021286" or "27525363" or "26910623" or "27221952" or "27316775" or "27389632" or "26669922" or "26975627" or "27429668" or "28197499" or "27919349" or "26976914" or "27543802" or "27998915" or "27098076" or "26795925" or "26820799" or "27671328" or "26733115" or "27462052" or "27698541" or "27055902" or "26608823" or "25810065" or "27070077" or "27620358" or "26900131" or "27919363" or "26577223" or "26496982" or "26892126" or "26589291" or "25580863" or "26652826" or "25799502" or "26227068" or "25649483" or "26704520" or "26520897" or "26255620" or "25514902" or "26458977" or "25891209" or "26474596" or "26178021" or "26714540" or "26195677" or "25857271" or "26178535" or "25904591" or "25681607" or "26109505" or "25809853" or "25274942" or "26516874" or "26437530" or "26121190" or "25532994" or "26135220" or "25935113" or "25944971" or "25572014" or "26411937" or "26623014" or "25978320" or "25819605" or "26788101" or "26633047" or "26656346" or "26402926" or "26228672" or "25670360" or "26436287" or "26044581" or "26112012" or "26116691" or "25034455" or "24882148" or "25931040" or "26146893" or "25658659" or "26011609" or "25953004" or "25815614" or "26066290" or "26161751" or "25273856" or "26203098" or "25936320" or "25952300" or "25687463" or "26687704" or "26423083" or "25559341" or "26603088" or "26112890" or "26027631" or "26135349" or "25936329" or "26564631" or "25862452" or "26648432" or "25856966" or "26319815" or "25997074" or "25787240" or "26160405" or "26297334" or "25906268" or "25952471" or "26079405" or "26047876" or "25443400" or "25716968" or "26174092" or "25330368" or "25989805" or "25760954" or "25907492" or "25905475" or "24681217" or "24831481" or "25126774" or "25333326" or "24630267" or "24906846" or "24611005" or "25623096" or "25216757" or "24085382" or "25120083" or "25264877" or "25156893" or "25709121" or "25516229" or "24531085" or "25195188" or "25122648" or "24430948" or "24993977" or "24645848" or "25152081" or "25468658" or "24936728" or "24287284" or "25074400" or "24572249" or "24652812" or "25139168" or "26316666" or "24192652" or "25056604" or "25347198" or "24350931" or "25082159" or "24742812" or "25034080" or "25163226" or "24600594" or "25190228" or "24800666" or "24504152" or "25536410" or "24781821" or "24668570" or "24327522" or "24676369" or "24449092" or "24476220" or "22739687" or "24667004" or "26982659" or "24263182" or "25176936" or "24720534" or "24806352" or "24206823" or "24728953" or "24743011" or "25210095" or "24452112" or "25600043" or "24325864" or "24944061" or "24853155" or "25341306" or "24529147" or "24662777" or "25335544" or "25097940" or "24721317" or "24632281" or "25425215" or "24712315" or "24300152" or "24378270" or "24321022" or "24025637" or "23246328" or "23070631" or "23805252" or "23560969" or "23196351" or "23294904" or "23510561" or "23919842" or "23018766" or "23866938" or "23677857" or "22345688" or "23058720" or "23458042" or "23517282" or "24296750" or "22664309" or "24217264" or "23551672" or "23565931" or "24028260" or "24244645" or "23623012" or "23302603" or "22975714" or "23463454" or "24099726" or "23332383" or "22971964" or "23947939" or "22770541" or "23541013" or "22954159" or "22144263" or "23758630" or "23841468" or "22633791" or "23480442" or "23647398" or "23357154" or "29805870" or "24024768" or "22842974" or "23137925" or "23628306" or "23357141" or "23991608" or "23506430" or "24094079" or "23459084").pm.	227

20	("23221025" or "24314361" or "23902756" or "23933695" or "23382331" or "23623643" or "23759268" or "23642324" or "24156549" or "23594919" or "23638783" or "23157464" or "23122768" or "23671433" or "23256179" or "22647482" or "24070336" or "24007717" or "23864194" or "24238499" or "22936204" or "23985183" or "24044467" or "23375686" or "23642926" or "23835301" or "24579506" or "23071140" or "24158101" or "23919812" or "23876348" or "23254630" or "23193114" or "23162007" or "23266621" or "23346951" or "22866937" or "22243772" or "22578720" or "21821844" or "21416117" or "23228012" or "22031213" or "22481692" or "22119890" or "22785138" or "22469073" or "22626876" or "22689050" or "23140032" or "22153777" or "22148868" or "22933212" or "22701100" or "25058963" or "22727798" or "22906083" or "22468143" or "22517421" or "22100028" or "22153979" or "22170216" or "22293316" or "22717466" or "22775419" or "22856164" or "22087585" or "22504754" or "22192156" or "23336188" or "22030117" or "21641813" or "22883727" or "22390808" or "22890973" or "22893402" or "23362564" or "22017319" or "21940276" or "23108199" or "22978800" or "22071413" or "22981166" or "22732744" or "22840347" or "22483404" or "22341486" or "20645024" or "22698766" or "23264238" or "22226369" or "22672914" or "22809114" or "22471945" or "22547221" or "22819352" or "22611827" or "22261195" or "22429228" or "22699246" or "21918828" or "22533665" or "22572422" or "22799293" or "21810288" or "23045823" or "22190137" or "22221517" or "22836151" or "23815022" or "22712740" or "22908087" or "22459551" or "21640350" or "20509032" or "21332946" or "20580254" or "21354724" or "22070582" or "19939652" or "20606469" or "22580728" or "22111468" or "20739064" or "21123766" or "21605293" or "21256834" or "21452966" or "21348923" or "21821527" or "22104509" or "21273557" or "20807166" or "21199964" or "21714932" or "21376325" or "22018443" or "21815708" or "21862153" or "21274756" or "22082357" or "22128042" or "20807459" or "21297494" or "21324888" or "20546164" or "21804350" or "20171845" or "21483226" or "21791073" or "21198993" or "21513480" or "21775565" or "21372492" or "21247526" or "21640762" or "22087679" or "21557673" or "21796141" or "21486801" or "21715698" or "21807618" or "22185846" or "21311603" or "21701079" or "21728907" or "21585569" or "21488754" or "21295847" or "21356148" or "21332996" or "21407165" or "21550577" or "22157149" or "21487128" or "21687917" or "20973765" or "21625818" or "21519634" or "20837371" or "21798967" or "21764497" or "21947005" or "21257349" or "21814848" or "21670376" or "20957811" or "19927160" or "21092333" or "21248454" or "20842602" or "19995791" or "19958209" or "20036859" or "20367964" or "21154329" or "20630099" or "19834104" or "20571286" or "19895880" or "20339287" or "19217673" or "21067805" or "20002875" or "20660987" or "20305563" or "20512225" or "20561029" or "19699070" or "19505972" or "20806200" or "19954956" or "20723257" or "20409259" or "20044742" or "20965319" or "21189169" or "20733267" or "19494836" or "20965889" or "19695854" or "20534947" or "20870177" or "20060988" or "20072809" or "20347450" or "20202105" or "19851300" or "20383691" or "21122701" or "21073069" or "20859896" or "20435353" or "20045568" or "20460242" or "20142527" or "20739726" or "20881421" or "20379119" or "20634651" or "20495954" or "20726197" or "20446878" or "20006530" or "20668250" or "20144596" or "21591389" or "20435231" or "20107198" or "20081323" or "20034957" or "20679142" or "20339369" or "20550064" or "20186243" or "22338441" or "20351074" or "20236527" or "19081646" or "19995404" or "20815157" or "20377162" or "20591696" or "20660284").pm.	253
21	("19726160" or "20956490" or "20482882" or "20375102" or "19798074" or "20200337" or "20670729" or "20719839" or "20648735" or "20964105" or "20005542" or "20063111" or "20170916" or "19528346" or "18325138" or "19092705" or "19204599" or "19285607" or "18824493" or "19231603" or "19436781" or "20209716" or "19423132" or "19331683" or "19672745" or "18270526" or "25998591" or "18845302" or "19761211" or "19273871" or "20139652" or "19380898" or "19656039" or "18608549" or "19196895" or "19165168" or	261

	"20129372" or "20065615" or "19873941" or "18932049" or "19252236" or "19008872" or "19154954" or "19671400" or "19439299" or "19535749" or "19114224" or "19493634" or "19627212" or "19247187" or "19398913" or "19254121" or "19827646" or "19827648" or "19219403" or "19209184" or "20172409" or "19096366" or "19253714" or "18479686" or "19286092" or "20514896" or "19835569" or "19286194" or "19605566" or "19026412" or "20131928" or "19393847" or "19469439" or "19380709" or "19443470" or "19702122" or "19017774" or "20020602" or "19429836" or "19922832" or "20183988" or "19273719" or "19552291" or "19725447" or "19675201" or "18715284" or "19159492" or "19402225" or "19070521" or "19122174" or "19537224" or "19476583" or "20583681" or "19628659" or "19520994" or "24683220" or "19331437" or "19825213" or "19558660" or "19251275" or "19399742" or "18649056" or "18372576" or "18472352" or "18379204" or "18461008" or "18799983" or "19186337" or "18728169" or "17714881" or "18258533" or "19009746" or "17714716" or "18240193" or "18689364" or "18460478" or "19167588" or "18601600" or "18838533" or "18317846" or "19301716" or "18469202" or "18332151" or "17399968" or "18782143" or "18502227" or "18609063" or "18054295" or "18173546" or "18727282" or "21291723" or "18654948" or "18164018" or "18385045" or "19169496" or "18481232" or "18714150" or "18446311" or "18377643" or "18616850" or "18388034" or "18191050" or "18670365" or "18385536" or "19166165" or "18974708" or "18926611" or "17901467" or "18719340" or "18442505" or "18172039" or "18309236" or "18426997" or "18806522" or "18219179" or "18397584" or "18274806" or "18070819" or "19064527" or "17981917" or "19000490" or "18681780" or "18714375" or "18277138" or "18684751" or "18506369" or "18241302" or "18762168" or "18548846" or "18346648" or "18495951" or "19161079" or "18376349" or "18700439" or "18309111" or "18806630" or "18302312" or "18378244" or "18479282" or "18826453" or "18356332" or "18791341" or "18603596" or "18700238" or "18727833" or "18564121" or "18350202" or "18223624" or "18334776" or "19157268" or "18684164" or "18364261" or "18718484" or "18720274" or "17953518" or "18727989" or "17123536" or "18334815" or "18160469" or "19011318" or "18430270" or "18803993" or "18796541" or "18243212" or "18420951" or "18246242" or "17517690" or "17526655" or "17510467" or "17986900" or "17160750" or "17209184" or "17557999" or "17641228" or "17718788" or "17490923" or "17513208" or "17186134" or "17311944" or "17324737" or "17435857" or "17199722" or "17972053" or "17380167" or "17161236" or "16616756" or "17526978" or "17988409" or "17105840" or "17264848" or "17985810" or "17664376" or "17217555" or "17233690" or "17563556" or "17546618" or "18217459" or "17985033" or "17386092" or "17050011" or "17826698" or "18260936" or "18260968" or "17394870" or "17353585" or "17468091" or "17935245" or "18156097" or "17721029" or "17299378" or "17167279" or "17961529" or "17143557" or "18074758" or "17919399" or "17762454" or "17908002" or "17379491" or "17803342" or "18080553" or "18082090" or "23074505" or "23074505" or "17306735" or "17645871" or "17625723" or "18038584" or "17536607").pm.	
22	("17705891" or "17884344" or "18158077" or "17673701" or "18180216" or "17431185" or "18000290" or "17719720" or "17445284" or "17123537" or "17334091" or "17215383" or "17824470" or "17911781" or "18154231" or "17996658" or "17606543" or "17341535" or "17578886" or "17705568" or "17383264" or "17889493" or "18095777" or "17576866" or "17644340" or "17081219" or "17903646" or "17227806" or "17900516" or "17629824" or "16970954" or "16540111" or "17623813" or "17903577" or "18019216" or "17407633" or "17725268" or "17124393" or "17927933" or "17366357" or "17569881" or "17317371" or "17265193" or "17996508" or "17552140" or "17275490" or "17935545" or "17143134" or "16855199" or "17016617" or "17101888" or "16648620" or "16988848" or "16465756" or "16802576" or "16549264" or "16979399" or "16716095" or "16808132" or "16356567" or "16574890" or "16722972" or "16528005" or "16873790" or "17001216" or "16443564" or "16713434" or "16858752" or "16881992" or "16009526" or "16458974" or "16516587" or	260

	"16961668" or "17085984" or "16945743" or "16799230" or "16990079" or "17027563" or "16509450" or "17784544" or "16736386" or "17173797" or "17203827" or "17191025" or "17633399" or "16368469" or "17019381" or "16709304" or "16931783" or "17144426" or "16821269" or "16864925" or "16095600" or "16703925" or "16978137" or "16704556" or "16529663" or "16532085" or "17323605" or "16882279" or "17007658" or "17130276" or "16603852" or "17109362" or "16575282" or "16631436" or "17333747" or "16433936" or "16705668" or "16849409" or "16412525" or "16910905" or "16636482" or "16728551" or "17145421" or "16556855" or "16741320" or "16721398" or "16740849" or "17046559" or "16935763" or "16964311" or "16796076" or "16860258" or "16482073" or "16860175" or "16735520" or "16464813" or "16875509" or "16990155" or "16466730" or "16269257" or "16271780" or "16510091" or "16375582" or "16639078" or "16858759" or "16246347" or "17274457" or "16293256" or "16963350" or "16783272" or "17001217" or "16879111" or "16750103" or "16520721" or "17001218" or "16719843" or "30000416" or "30000420" or "30000421" or "17179595" or "15699227" or "16123850" or "15857272" or "16389851" or "15774702" or "16285998" or "16106049" or "16030523" or "16092050" or "15333620" or "15975790" or "16157786" or "16583932" or "15986112" or "15766689" or "15891275" or "16309370" or "15920065" or "16030068" or "16555576" or "16014090" or "15656878" or "16176168" or "16009795" or "15687443" or "16144549" or "15777550" or "15836544" or "15887425" or "16154486" or "15703509" or "20527433" or "16003294" or "16602438" or "15827865" or "15798121" or "15864114" or "15769763" or "16207260" or "15955465" or "16034448" or "16093405" or "16305587" or "15309416" or "16541719" or "15703504" or "15741835" or "15826890" or "16227145" or "15846260" or "15942550" or "15910869" or "16123315" or "16020749" or "16014208" or "15691040" or "16204427" or "15988705" or "15658097" or "15746228" or "16141286" or "15935359" or "16279505" or "16036545" or "16142019" or "15984375" or "15781755" or "15508014" or "15932697" or "15749623" or "15619173" or "16220697" or "15864113" or "15830122" or "15982505" or "15536473" or "16477767" or "16141779" or "15817854" or "15547298" or "15824910" or "15776996" or "15904881" or "15674310" or "15671032" or "15823288" or "15907855" or "15777557" or "16020744" or "16041450" or "15876802" or "16407605" or "15797163" or "15990755" or "16400780" or "15857334" or "15703508" or "16602445" or "16061941" or "16344385" or "15687137" or "15514264" or "15110745" or "14746413" or "15135857" or "15375596" or "15226625" or "15199439" or "14968337" or "17516697" or "14722362" or "15055240").pm.	
23	("15186959" or "15611671" or "15184285" or "14604830" or "15687407" or "14709677" or "15313964" or "15353416" or "15628647" or "15540545" or "15111542" or "15177701" or "15639694" or "15096336" or "14709372" or "15164320" or "15462690" or "15317046" or "15177301" or "15005509" or "15103514" or "15282667" or "15717141" or "15623679" or "15037535" or "15300004" or "15073652" or "15177127" or "15327691" or "15565928" or "15446673" or "15064110" or "15687405" or "15111372" or "15466496" or "15547022" or "15648805" or "15340676" or "15119832" or "15012019" or "15327591" or "15128051" or "15077569" or "14972073" or "14693979" or "15649295" or "15631531" or "14999031" or "14757692" or "15291694" or "15214960" or "15242063" or "15119835" or "15475176" or "15184689" or "14693778" or "15212768" or "15066061" or "15195867" or "15257723" or "15554949" or "15564707" or "14996476" or "15238453" or "15455181" or "15853118" or "15765037" or "15458693" or "15059707" or "15199349" or "15174739" or "15211364" or "15158296" or "15532130" or "15334371" or "15630635" or "15194017" or "15247960" or "15277400" or "14988449" or "15630632" or "15326068" or "14766115" or "15223989" or "15633853" or "14985690" or "15598092" or "14962437" or "14967166" or "15199031" or "15256790" or "14724558" or "15617195" or "15884199" or "15225529" or "14740240" or "15477786" or "15136072" or "15650343" or "15220952" or "15531664" or "15639688" or "15682353" or "15639697" or "14994781" or "15566253" or "14656730" or "15530458" or	263

	"12889988" or "15222635" or "14564313" or "14975168" or "14639019" or "14699858" or "12878452" or "12646733" or "12966126" or "12832311" or "15116471" or "12707402" or "12939356" or "14624049" or "12872711" or "12829221" or "12776271" or "12714863" or "12877676" or "14578611" or "14625131" or "12745196" or "14593381" or "12671581" or "14501025" or "14735020" or "12648031" or "14663597" or "12756407" or "12706470" or "14618093" or "14564085" or "12600905" or "12791613" or "14629322" or "14644397" or "12886133" or "12737782" or "14506545" or "14671811" or "12755140" or "14725205" or "12923015" or "12967690" or "12769254" or "12637115" or "12601531" or "12806724" or "14709849" or "12687649" or "12689687" or "12915428" or "12975599" or "12824094" or "15190632" or "12801604" or "12963571" or "12535745" or "14608054" or "12614415" or "12754449" or "12708250" or "12887727" or "12802899" or "12885701" or "24944394" or "12939864" or "12511745" or "14522571" or "12819993" or "12524233" or "12933534" or "14704504" or "12859032" or "14704511" or "12727230" or "15562735" or "12818409" or "12840178" or "12932630" or "14671463" or "12719276" or "14638534" or "12700893" or "14624727" or "12588950" or "14704508" or "12756561" or "12492446" or "12750110" or "12735892" or "12544724" or "12639974" or "14693301" or "12548313" or "12668913" or "14679272" or "12705620" or "12805251" or "12600847" or "12887719" or "14597936" or "12488246" or "12938249" or "12681161" or "12742284" or "12208481" or "11928067" or "11947911" or "12503475" or "12196085" or "11897443" or "12436707" or "12356004" or "11834528" or "12473547" or "12081819" or "11922642" or "12324284" or "12149041" or "12494016" or "12494221" or "12417832" or "12480773" or "12223326" or "11910049" or "12011578" or "12241128" or "12356387" or "12044587" or "12141531" or "12116743" or "12082364" or "12364448" or "11840368" or "12118856" or "12182255" or "12534449" or "11910299" or "12237025" or "12118909" or "12063444" or "12187397" or "11982953" or "12138367" or "12817199" or "12417279" or "11858478" or "12357133" or "11966345" or "11901049" or "12208171" or "12470198" or "12450888" or "11833047" or "12356389").pm.	
24	("11923123" or "12001831" or "12473254" or "12219187" or "12073409" or "12164469" or "12366606" or "12409747" or "11985406" or "12474722" or "12052483" or "12077742" or "12126462" or "12122545" or "12029990" or "12401533" or "12218765" or "12217263" or "11782586" or "12119208" or "12165052" or "12464375" or "19667596" or "12394687" or "11790693" or "11888526" or "12045169" or "12423709" or "11821719" or "12427649" or "11834531" or "12626213" or "12027776" or "11833826" or "11900713" or "12137985" or "12435337" or "12202481" or "12046033" or "12390056" or "12195798" or "12123487" or "12419484" or "11815311" or "11879241" or "12172393" or "12048142" or "12403804" or "12087575" or "11434800" or "11324369" or "11481566" or "11711272" or "11213091" or "11571834" or "11293949" or "11248748" or "11434192" or "12063772" or "11328302" or "11325764" or "11243306" or "11253736" or "11255880" or "11181283" or "11320358" or "11213168" or "11230787" or "11412677" or "11500190" or "11794823" or "11688949" or "11412807" or "11358159" or "11933923" or "11721164" or "11928592" or "11182189" or "11338344" or "11376309" or "11730829" or "11297296" or "11556929" or "11480153" or "11900393" or "11860839" or "12439378" or "11246583" or "11501710" or "12063773" or "11257254" or "11374884" or "11498127" or "11474894" or "11427213" or "11675946" or "11678968" or "11583720" or "11230837" or "11438195" or "11324372" or "11368750" or "11520486" or "11136703" or "11136952" or "11828923" or "11791398" or "11347120" or "11570118" or "11735685" or "11772284" or "11738396" or "11507971" or "11573752" or "11304891" or "11488557" or "11383131" or "11683775" or "11586494" or "11298777" or "11773670" or "11775412" or "11483625" or "11572515" or "11379378" or "11405386" or "11383320" or "11449080" or "11704319" or "11253155" or "11582971" or "11520484" or "12596954" or "10744590" or "10949188" or "10785876" or "11082147" or "10980587" or "10729389" or "10845875" or "10909990" or "11128726" or "10911769" or "11031214" or	263

	"10947889" or "11044482" or "11252539" or "10607765" or "11798745" or "10641021" or "10849014" or "10953872" or "10729393" or "10998931" or "10781644" or "10751747" or "10962844" or "10922444" or "11084779" or "11174884" or "10781516" or "10819699" or "10694781" or "11143768" or "10972631" or "11214517" or "10893651" or "10942873" or "10785571" or "10882184" or "10944066" or "10821140" or "10832141" or "10662741" or "10794587" or "10686277" or "10702770" or "11200606" or "10935153" or "11065222" or "11201819" or "10653927" or "10720164" or "10999996" or "10833789" or "11082213" or "10631147" or "10995978" or "10845868" or "10672131" or "10955373" or "10685126" or "10567851" or "10872897" or "10947880" or "10993000" or "10865825" or "10869898" or "10736328" or "10919931" or "11140287" or "10642706" or "11191012" or "10843151" or "10922437" or "11173718" or "11930385" or "10960724" or "11076412" or "11094610" or "10946010" or "11082978" or "10980214" or "11213534" or "10781646" or "10716139" or "10812582" or "10895106" or "10867084" or "11234461" or "11005639" or "10795432" or "10894817" or "11194009" or "11118175" or "10978268" or "10999646" or "10704632" or "11126143" or "10656841" or "11155392" or "10935359" or "10948881" or "11092283" or "10837282" or "10648260" or "10869546" or "10687879" or "10487490" or "10525487" or "10332687" or "10030394" or "10356595" or "10527295" or "10224985" or "10514155" or "10566571" or "10482152" or "10705560" or "10408004" or "10389464" or "10511836" or "10621930" or "10610245" or "10593347" or "10208056" or "10455066" or "10095984" or "10493153" or "10941422" or "11138933" or "10575807" or "10615519" or "10551206").pm.	
25	("10406451" or "10084016" or "10551207" or "10611909" or "10636448" or "10081033" or "10024863" or "10381159" or "10447222" or "10570905" or "10487500" or "10075320" or "10208479" or "10426702" or "10372308" or "10376199" or "9927392" or "10448768" or "10368792" or "9973073" or "10199475" or "10482153" or "10606208" or "11122694" or "10580395" or "10529757" or "10439497" or "10547136" or "10565602" or "10690329" or "10395624" or "10386509" or "10493319" or "10498131" or "10735582" or "11279918" or "10584419" or "9880433" or "10522984" or "10408999" or "11122686" or "10529129" or "10475870" or "10094580" or "10322565" or "10503616" or "10572218" or "10394436" or "9920511" or "11503831" or "10500014" or "10445837" or "9578244" or "9603795" or "9642116" or "10933428" or "9893006" or "9656651" or "9819102" or "9554107" or "9647874" or "9651456" or "9688439" or "9856223" or "9756426" or "9646096" or "9484771" or "9713078" or "9766706" or "9465706" or "9619005" or "9434647" or "9519020" or "9588433" or "10085714" or "9701246" or "9805562" or "9611906" or "9424571" or "9684746" or "9800230" or "9830842" or "9709409" or "9645827" or "9598575" or "9808796" or "9699905" or "9789853" or "9882961" or "9622280" or "9596233" or "9533233" or "9457050" or "9839103" or "10335028" or "9740341" or "9531256" or "9551392" or "9465989" or "9678774" or "9711171" or "9738161" or "9861590" or "9915661" or "9845140" or "9809137" or "9504445" or "9651560" or "9571357" or "9550499" or "9580388" or "9791635" or "9497178" or "9808644" or "9622289" or "9792666" or "9799908" or "9706584" or "9424570" or "9699897" or "9714133" or "9573756" or "9484425" or "9729642" or "9688236" or "9781976" or "9614611" or "11907931" or "9571339" or "9419190" or "9082037" or "9105570" or "9262494" or "9505937" or "9117913" or "9081694" or "9409936" or "9050775" or "9303663" or "9340086" or "9358426" or "9098596" or "9211063" or "9297170" or "9183227" or "9372993" or "9476817" or "8994481" or "9174230" or "9499282" or "9192911" or "9033222" or "9103094" or "9409324" or "9476683" or "9157944" or "9110123" or "9280882" or "9252956" or "9409937" or "9043971" or "9245545" or "9101108" or "9603697" or "9183304" or "9379730" or "9048115" or "9217585" or "9267993" or "9012636" or "9354440" or "9355889" or "9189647" or "9340188" or "9411422" or "9062840" or "9409318" or "11517479" or "9011785" or "9024732" or "9091527" or "9395808" or "9358017" or "9519126" or "9108789" or "9192227" or "9426354" or "9392426" or "9178790" or "9039882" or "9217583" or "9182655" or "9271481" or "9029226" or "9217584" or "9135930" or "9377752" or "9372997" or "9100140" or "9380384" or "9334463" or "9152737"	302

	or "8970509" or "8650967" or "8974213" or "8979120" or "8696963" or "8686314" or "8839657" or "8792826" or "8847874" or "8740948" or "8807702" or "8998799" or "8880894" or "8783032" or "8678927" or "8561065" or "9006811" or "9083865" or "8903864" or "8679872" or "8784361" or "8917325" or "8664620" or "9010744" or "9015672" or "9091946" or "8724114" or "8889490" or "8843050" or "8814524" or "9003110" or "9008925" or "8851938" or "8999461" or "8635262" or "8681371" or "8729584" or "8759095" or "8722158" or "8685519" or "8620340" or "8864962" or "8974552" or "8867362" or "9302828" or "8693169" or "9395578" or "8964318" or "8992854" or "8722744" or "8762687" or "8759943" or "8768633" or "8957200" or "8774269" or "9269097" or "8734860" or "8609958" or "8572841" or "8656540" or "8721863" or "8596497" or "8824022" or "8676819" or "8683643" or "8843892" or "8769353" or "8548413" or "8902157" or "8644746" or "8726633" or "8647378" or "8929255" or "8656142" or "8677767" or "8681156" or "8949979" or "8930228" or "9225220" or "9226461" or "8787369" or "8642233" or "7503003" or "7561980" or "7652481" or "7616853" or "7604783" or "7478472" or "7614520" or "7867176" or "7752281" or "7676216" or "7491685" or "7702230" or "7585299" or "7474191" or "7627699" or "7485220").pm.	
26	("7711988" or "7723899" or "7606845" or "7476293" or "7572747" or "7573090" or "7738190" or "8770321" or "7858682" or "8620056" or "8610084" or "8520892" or "7597541" or "7583549" or "7604798" or "8838332" or "7772062" or "7866933" or "8522729" or "7484265" or "7642011" or "8579620" or "7828385" or "7494939" or "7575771" or "7778537" or "7769402" or "7642176" or "7781495" or "9827075" or "7801966" or "7606643" or "7571764" or "7604799" or "7585284" or "7578834" or "7489031" or "7625348" or "7625364" or "7852377" or "7490996" or "8520275" or "7897787" or "7840078" or "7672076" or "7604805" or "7699185" or "8549923" or "7634440" or "7662446" or "7604800" or "7650079" or "7595099" or "7744541" or "8871266" or "8561652" or "7893737" or "7676762" or "7579744" or "7548643" or "8867781" or "8296711" or "7966894" or "7933263" or "7797219" or "8012881" or "7860175" or "8163645" or "7730737" or "7979101" or "8282821" or "7954676" or "7809768" or "8186654" or "7802728" or "7919081" or "8017448" or "7960472" or "8143006" or "10150300" or "7886290" or "7979498" or "7517836" or "8012882" or "7726691" or "8048085" or "7910281" or "8270757" or "8274473" or "7517834" or "7710201" or "8122946" or "8181150" or "8016034" or "19489169" or "7882658" or "8017469" or "8113822" or "8066704" or "8002848" or "8047526" or "8082956" or "7517829" or "8004802" or "8185885" or "7988779" or "7866081" or "7863486" or "8017474" or "8044949" or "8060385" or "8208397" or "7918304" or "7816977" or "8202188" or "8066357" or "8297199" or "8147544" or "7857367" or "7914265" or "8106688" or "7997692" or "7918305" or "8064900" or "7931039" or "7980716" or "7917813" or "7968073" or "7803063" or "8172843" or "7989900" or "8334949" or "8518573" or "8425468" or "8328379" or "8317435" or "8519205" or "8410106" or "8011962" or "8378736" or "8348061" or "8513315" or "8363164" or "8297546" or "7681515" or "8342189" or "8254266" or "8289097" or "8337460" or "8144284" or "8405029" or "8450292" or "8463785" or "8440848" or "8409092" or "8502905" or "8361860" or "8492598" or "8486007" or "8292095" or "8320981" or "8399083" or "8036577" or "8512044" or "8503516" or "8518354" or "8457250" or "8506890" or "8235557" or "7941761" or "8257617" or "8366183" or "8334804" or "24921113" or "8465806" or "8213502" or "7732293" or "8240474" or "8482062" or "8370576" or "8480942" or "8503586" or "8460835" or "8460836" or "8439103" or "8480943" or "8423226" or "8287156" or "8506889" or "8363165" or "8495121" or "8345800" or "8268084" or "8446869" or "8144283" or "8480959" or "8501483" or "8239396" or "8443740" or "8274921" or "8111502" or "8114261" or "8403359" or "8430625" or "8336217" or "1516292" or "1536350" or "1731860" or "1603147" or "1407133" or "10148720" or "10146977" or "1626496" or "1738814" or "1516875" or "1291621" or "1635494" or "1736560" or "1425090" or "1302897" or "1475701" or "1348666" or "1466918" or "1449569" or "1296541" or "1509220" or "1632851" or "1482576" or "1405284" or "1534257" or "1420786" or "1479313" or "1390323" or "1360061" or "1341866" or "1619198" or "1334574" or "1587400" or "1546864" or "1327742" or "1596851" or "1483414" or "1597213" or "1546702" or "1477994"	306

	or "1315570" or "1591376" or "1734550" or "1296550" or "1352930" or "1727908" or "1576582" or "1310566" or "1583818" or "1542261" or "1341147" or "1506163" or "1414915" or "1534286" or "1359022" or "1609919" or "1550087" or "1499466" or "1468089" or "1424049" or "1306590" or "1296548" or "1610539" or "1466665" or "1285697" or "1576628" or "1518504" or "1734676" or "1360559" or "1488408" or "1581939" or "1429490" or "1477992" or "1375692" or "1342268" or "1480808" or "1627020" or "1329482" or "1513124" or "1308800" or "2011075" or "1790033" or "1880840" or "1899938" or "2004412" or "1790627" or "2040869" or "2040207" or "1751710" or "1880861" or "1785285" or "1655015" or "1750429" or "1992960" or "1754864" or "1915205" or "2044648" or "1775703" or "1832729" or "1879732" or "2025730" or "1991249" or "1813637").pm.	
27	("2044257" or "1927944" or "1903849" or "1858187" or "1659171" or "1854109" or "1750045" or "1660530" or "1911720" or "1747553" or "1680391" or "1998646" or "1763862" or "2071804" or "1771957" or "1911722" or "1767696" or "2029780" or "1899896" or "1861922" or "1650421" or "1936105" or "1842523" or "1685207" or "1809892" or "1910254" or "1988005" or "2016863" or "2071797" or "1819418" or "2002818" or "1941853" or "1880482" or "1898717" or "1746296" or "1933004" or "1872664" or "2073672" or "2320553" or "1965418" or "2296124" or "2181692" or "2073668" or "2301164" or "2371635" or "2371640" or "2374043" or "2218778" or "2240916" or "2262252" or "1670530" or "2218650" or "2308515" or "2391728" or "2353352" or "2106919" or "2363952" or "2271263" or "2254766" or "2372967" or "2082621" or "2188241" or "2332817" or "2267362" or "2083935" or "2286856" or "2268697" or "2343674" or "2402946" or "2206030" or "2243428" or "2380678" or "2073969" or "2243435" or "2360291" or "2229942" or "2332214" or "2275284" or "2271215" or "2184712" or "2154513" or "2390138" or "2184006" or "2344298" or "2186451" or "2109783" or "2205799" or "2206036" or "2212879" or "2359503" or "2405605" or "2919540" or "2912434" or "2784236" or "2798366" or "2607722" or "2733124" or "2784930" or "2787564" or "2709545" or "2606449" or "2736571" or "2677611" or "2709886" or "2585960" or "2773118" or "2495023" or "2600493" or "2650524" or "2600495" or "2925333" or "2676653" or "2734659" or "2807707" or "2628954" or "2712708" or "2725596" or "2809554" or "2638436" or "2815034" or "2689125" or "2648923" or "2802415" or "2531881" or "2711758" or "2692571" or "2551157" or "2712858" or "2736820" or "2812486" or "2690048" or "2715556" or "2811031" or "3180583" or "3141689" or "3185048" or "3188982" or "3231844" or "3202019" or "3288476" or "3041982" or "3224001" or "3226223" or "3414733" or "3267710" or "3348758" or "3247710" or "3042197" or "3178382" or "3378557" or "3363513" or "3264450" or "3389027" or "3188664" or "3202023" or "3042201" or "3348841" or "3810359" or "3494320" or "3827979" or "3501638" or "3310570" or "3824155" or "3118060" or "3695108" or "3661536" or "2961916" or "2951547" or "2885572" or "3812209" or "3678235" or "3826039" or "3632751" or "3687114" or "3120215" or "3680913" or "3628256" or "3110726" or "2886738" or "3941501" or "3940678" or "2873641" or "3535128" or "3543817" or "3714568" or "3457291" or "2871933" or "3955643" or "3946460" or "3535814" or "3728290" or "3961687" or "3820578" or "3524520" or "3526270" or "3940684" or "3951912" or "3513134" or "2952074" or "3718994" or "3534334" or "2955619" or "3711914" or "3953435" or "2873741" or "4062130" or "2934849" or "3874756" or "4008771" or "4068979" or "4072526" or "4058254" or "4021807" or "4015511" or "3835855" or "4035101" or "4023047" or "3974449" or "3891266" or "4046317" or "3907645" or "6521201" or "6730738" or "6590003" or "6323545" or "6491620" or "6531358" or "6731433" or "6697533" or "6531453" or "6723318" or "6368715" or "6331021" or "6492568" or "6465119" or "6469537" or "6422891" or "6521204" or "6374831" or "6373280" or "6710113" or "6588552" or "6361300" or "6731432" or "6480065" or "6573160" or "6860476" or "6414238" or "6645197" or "6685165" or "6659567" or "6228091" or "6680537" or "6873209" or "6665222" or "6844393" or "6579340" or "6396733" or "6648889" or "7046692" or "7123589" or "7183053" or "6125861" or "7136019" or "6132433" or "7058778" or "6752528" or "7092575" or "7138620" or "7079619" or "7042218" or "7154491" or "7120357" or "6818977" or "7170492" or "7068811" or "7175379" or "7225167" or "6112635" or "7259072" or	308

	"6945753" or "7303665" or "6118533" or "7301182" or "7339867" or "7322621" or "7033903" or "7289368" or "7293252" or "7329510" or "7052358" or "7464557" or "7298021" or "7225568" or "7263187" or "7240364" or "6781999" or "7247985" or "7314922" or "6118924" or "7355782" or "7395296" or "7353267" or "6992525" or "7428376" or "7392357" or "7365983" or "7245576").pm.	
28	("7456148" or "7461569" or "7003789" or "7363194" or "7404060" or "7395212" or "7419983" or "7360532" or "6105293" or "7433204" or "6106825" or "7000350" or "7454136" or "6261774" or "7382258" or "7380228" or "525299" or "472597" or "758999" or "523488" or "453047" or "499929" or "217273" or "486513" or "161872" or "218071" or "227426" or "549252" or "461210" or "515458" or "86108" or "425874" or "513825" or "642859" or "207905" or "705116" or "214000" or "756589" or "738551" or "359229" or "717136" or "744681" or "622025" or "276566" or "366234" or "217108" or "215033" or "919596" or "417264" or "928817" or "928814" or "198911" or "402755" or "414113" or "884862" or "909482" or "610428" or "920421" or "871667" or "835511" or "412058" or "850311" or "597353" or "580920" or "856227" or "579623" or "958089" or "1269099" or "1106167" or "1244378" or "979847" or "946691" or "183321" or "768657" or "932167" or "179295" or "1257681" or "1017536" or "992559" or "958648" or "956689" or "1261061" or "1013392" or "132862" or "188434" or "1251811" or "1261427" or "951135" or "174850" or "183322" or "966076" or "1014624" or "770089" or "947672" or "1024628" or "187428" or "959629" or "178813" or "813212" or "947671" or "971346" or "792498" or "975971" or "1022601" or "178981" or "1144181" or "1096564" or "803123" or "803015" or "172533" or "1144817" or "164351" or "168737" or "1221379" or "1133650" or "171564" or "1180126" or "1107622" or "1105982" or "1157505" or "1057908" or "1104153" or "46958" or "1218738" or "1055951" or "1132094" or "1109485" or "47120" or "4377199" or "4810415" or "4819522" or "4548792" or "4813182" or "4548616" or "4421665" or "4810110" or "4427862" or "4828565" or "4424231" or "4548621" or "4425852" or "4548623" or "4603103" or "4370795" or "4824624" or "4281944" or "4371219" or "4422442" or "4366521" or "4456570" or "4373491" or "4442440" or "4445779" or "4821152" or "4366188" or "4424128" or "4598837" or "4369519" or "4377403" or "4821138" or "4449718" or "4600585" or "4371664" or "4691568" or "4763632" or "4801348" or "4581362" or "4719577" or "4726137" or "4121135" or "4693217" or "4695649" or "4585638" or "4715262" or "4578640" or "4357674" or "4763484" or "4605045" or "4351694" or "4742851" or "4771864" or "4766416" or "4760474" or "4741607" or "4755806" or "4550038" or "5019626" or "4665817" or "5048273" or "4635921" or "4642637" or "4145246" or "4110639" or "4564745" or "4551212" or "4657673" or "5049870" or "5073094" or "4678573" or "4335955" or "4340015" or "4649059" or "5051656" or "4344330" or "4563228" or "4565986" or "5009984" or "4112223" or "5072454" or "5081824" or "4542788" or "4563491" or "4665218" or "4649021" or "5539637" or "5567019" or "5132448" or "5556474" or "5094655" or "4257675" or "5121121" or "5563708" or "5574509" or "4945162" or "5546857" or "5132453" or "5095238" or "4937603" or "5132459" or "5568147" or "5108055" or "5097214" or "5132445" or "5443911" or "5465463" or "5493461" or "5420082" or "5004789" or "5508171" or "5450912" or "4914806" or "5452617" or "4246109" or "5782537" or "5774257" or "5823719" or "5396178" or "4987837" or "5819156" or "5385981" or "5822873" or "4306948" or "4386628" or "5304406" or "5640427" or "4888159" or "5682336" or "4241117" or "6052854" or "6040635" or "6031907" or "6019837" or "6018544" or "5988709" or "5957695" or "14328911" or "14217037" or "14232613" or "14235883" or "14119979" or "14074817" or "14047397" or "14104298" or "13606150" or "14911391" or "20722144").pm.	164
29	or/18-28	2840
30	limit 17 to (conference abstract or conference paper or "conference review")	288

31	limit 30 to yr="2018 -Current"	70
32	31 not 29	70
33	17 not (29 or 30)	205
34	from 32 keep 1-70	70

Original search for Cochrane (Wiley)

	Search	Results
	((familia* or type* 2 or type* 2s or type* ii or type iis or type* iia* or type* iib* or essential* or autosomal dominant or genetic*) NEAR/3 (hypercholesterolemi* or hypercholesterolaemi* or hyperlipoproteinemi* or hyperlipoproteinaemi* or hyper-cholesterolemi* or hyper-cholesterolaemi* or hyper-lipoproteinemi* or hyper-lipoproteinaemi* or apolipoprotein-b* or dyslipidemi*)):ti,ab,kw	2152
2	(HoFH or HFH or HzFH or HeFH or HHF):ti,ab,kw	372
3	((extreme* or rare* or severe* or homozyg* or homo-zygo* or heterozygo* or hetero-zygo*) NEAR/3 (hypercholesterolemi* or hypercholesterolaemi* or hyperlipoproteinemi* or hyperlipoproteinaemi* or hyper-cholesterolemi* or hyper-cholesterolaemi* or hyper-lipoproteinemi* or hyper-lipoproteinaemi* or apolipoprotein-b*)):ti,ab,kw	545
4	((hyperbetalipoproteinemi* or hyperbetalipoproteinaemi* or hyper-beta-lipoproteinemi* or hyper-beta-lipoproteinaemi* or lipoproteinemi* or lipoproteinaemi*) NEAR/3 (hyper-low* or hyper-beta* or hyperlow* or hyperbeta* or ldl receptor disorder*)):ti,ab,kw	8
5	lipoid gout*:ti,ab,kw	0
6	(tendon* NEAR/2 (xanthoma* or xanthogranulomatos*)):ti,ab,kw	11
7	((heterozygo* or hetero-zygo*) NEAR/2 FH):ti,ab,kw	59
8	#1 or #2 or #3 or #4 or #5 or #6 or #7	2436
9	((sex* or gender* or man or men or male* or woman or women or female*) NEAR/3 (difference* or different or characteristic* or ratio* or factor* or imbalanc* or issue* or both or specific* or disparit* or dependen* or gap or gaps or influenc* or discrepan* or distribut* or composition* or variability or comparison* or accept* or barrier* or perception* or perceiv* or between* or treat* or alirocumab or evolocumab or statin or atorvastatin or rosuvastatin or simvastatin or ezetimibe or ezetimib or niacin or enduracin or nicamin or nicobid or nicocap or nicolar or nicotinate or nicotinic or bile acid sequestrant or bempedoic acid or lomitapide or mipomersen or apheresis or PCSK9 inhibitor or anticholesteremic* or hypocholesteremic* or hmg-coa or hydroxymethylglutaryl or hydroxymethylglutaryl-coa or hydroxymethylglutaryl-coenzyme or (cholesterol NEAR/2 inhibitor*))):ti,ab,kw	86557
10	((men or men's) NEAR/2 women*):ti,ab,kw	17051
11	((gender* NEXT related) or (gender* Next based)):ti,ab,kw	415
12	#9 or #10 or #11	98335
13	#8 and #12	254
14	EMBASE:AN	552478
15	PUBMED:AN	673088
16	#14 or #15	1016113
17	#13 not #16	53

Original search for PubMed (NLM)

	Search	Results
1	(familia*[Text Word]) OR type* 2[Text Word]) OR type* 2s[Text Word]) OR type* ii[Text Word]) OR type iis[Text Word]) OR type* iia*[Text Word]) OR type* iib*[Text Word]) OR essential*[Text Word]) OR autosomal dominant[Text Word]) AND hypercholesterolemi*[Text Word] OR hypercholesterolaemi*[Text Word] OR hyperlipoproteinemi*[Text Word] OR hyperlipoproteinaemi*[Text Word] OR hyper-cholesterolemi*[Text Word] OR hyper-cholesterolaemi*[Text Word] OR hyper-lipoproteinemi*[Text Word] OR hyper-lipoproteinaemi*[Text Word] OR apolipoprotein-b*[Text Word] OR dyslipidemi*[Text Word]	67, 005
2	HoFH[Text Word] OR HFH[Text Word] OR HzFH[Text Word] OR HeFH[Text Word] OR HHF[Text Word]	1091
3	(extreme*[Text Word] OR rare*[Text Word] OR severe*[Text Word] OR homozyg*[Text Word] OR homo-zygo*[Text Word] OR heterozygo*[Text Word] OR hetero-zygo*[Text Word]) AND (hypercholesterolemi*[Text Word] OR hypercholesterolaemi*[Text Word] OR hyperlipoproteinemi*[Text Word] OR hyperlipoproteinaemi*[Text Word] OR [Text Word] OR apolipoprotein b*[Text Word])	5,160
4	((hyperbetalipoproteinemi*[Text Word] OR hyperbetalipoproteinaemi[Text Word]) OR (lipoproteinaemi*[Text Word] OR lipoproteinemi*[Text Word] OR lipoproteinaemi*[Text Word])) AND (hyperlow*[Text Word] OR hyperbeta*[Text Word] OR ldl receptor disorder*[Text Word])	123
5	lipoid gout*[Text Word]	4
6	tendon* xanthoma*[Text Word]) OR tendon* xanthogranulomatos*[Text Word]	383
7	**((heterozygo*[Text Word] or hetero-zygo*[Text Word]) AND FH[Text Word])**	1,399
8	#1 or #2 or #3 or #4 or #5 or #6 or #7	69,112
9	(sex[Text Word] OR sexes[Text Word] OR gender*[Text Word] OR man[Text Word] OR men[Text Word] OR male*[Text Word] OR woman[Text Word] OR women[Text Word] OR female*[Text Word]) AND (difference*[Text Word] OR different[Text Word] OR characteristic*[Text Word] OR ratio*[Text Word] OR factor*[Text Word] OR imbalanc*[Text Word] OR issue*[Text Word] OR both[Text Word] OR specific*[Text Word] OR disparit*[Text Word] OR dependen*[Text Word] OR gap[Text Word] OR gaps[Text Word] OR influenc*[Text Word] OR discrepan*[Text Word] OR distribut*[Text Word] OR composition*[Text Word] OR variability[Text Word] OR comparison*[Text Word] OR accept*[Text Word] OR barrier*[Text Word] OR perception*[Text Word] OR perceiv*[Text Word] OR between*[Text Word] OR treat*[Text Word] OR alirocumab[Text Word] OR evolocumab[Text Word] OR statin[Text Word] OR atorvastatin[Text Word] OR rosuvastatin[Text Word] OR simvastatin[Text Word] OR ezetimibe[Text Word] OR ezetimib[Text Word] OR niacin[Text Word] OR enduracin[Text Word] OR nicamin[Text Word] OR nicobid[Text Word] OR nicocap[Text Word] OR nicolar[Text Word] OR nicotinate[Text Word] OR nicotinic[Text Word] OR bile acid sequestrant[Text Word] OR bempedoic acid[Text Word] OR lomitapide[Text Word] OR mipomersen[Text Word] OR apheresis[Text Word] OR PCSK9 inhibitor[Text Word] OR anticholesteremic*[Text Word] OR hypocholesteremic*[Text Word] OR hmg-coa[Text Word] OR	8,374,455

	hydroxymethylglutaryl[Text Word] OR hydroxymethylglutaryl-coa[Text Word] OR hydroxymethylglutaryl-coenzyme[Text Word] OR cholesterol inhibitor*[Text Word])	
10	gender-related [Text Word] OR gender-based [Text Word]	8,318
11	#9 or #10	8,374,824
12	#8 and #11	39, 079
13	("2020/07/13"[Date - Create] : "3000"[Date - Create])	37,536
13	#12 and #13	41

Original search for Scopus (Elsevier)

	Search	Results
1	((TITLE-ABS-KEY ((familia* OR type*-2 OR type*-2s OR type-ii OR type-iis OR type-iia* OR type-iib* OR essential* OR autosomal-dominant) W/3 (hypercholesterolemi* OR hypercholesterolaemi* OR hyperlipoproteinemi* OR hyperlipoproteinaemi* OR hyper-cholesterolemi* OR hyper-cholesterolaemi* OR hyper-lipoproteinemi* OR hyper-lipoproteinaemi* OR apolipoprotein-b*))) OR (TITLE-ABS-KEY (HoFH or HFH or HzFH or HeFH or HHF)) OR (TITLE-ABS-KEY (extreme* OR rare* OR severe* OR homozyg* OR homo-zygo* OR heterozygo* OR hetero-zygo*) W/3 (hypercholesterolemi* OR hypercholesterolaemi* OR hyperlipoproteinemi* OR hyperlipoproteinaemi* OR hyper-cholesterolemi* OR hyper-cholesterolaemi* OR hyper-lipoproteinemi* OR hyper-lipoproteinaemi* OR apolipoprotein-b*)) OR (TITLE-ABS-KEY (hyperbetalipoproteinemi* OR hyperbetalipoproteinaemi* OR hyper-beta-lipoproteinemi* OR hyper-beta-lipoproteinaemi* OR ((lipoproteinemi* OR lipoproteinaemi*) W/3 (hyper-low* OR hyper-beta* OR hyperlow* OR hyperbeta*)) OR ldl AND receptor AND disorder*)) OR (TITLE-ABS-KEY (lipoid AND gout*)) OR (TITLE-ABS-KEY ((tendon*) W/2 (xanthoma* OR xanthogranulomatos*))) OR (TITLE-ABS-KEY ((heterozygo* OR hetero-zygo*) W/2 (fh)))) AND ((TITLE-ABS-KEY ((sex* OR gender* OR man OR men OR male* OR woman OR women OR female*) W/3 (difference* OR different OR characteristic* OR ratio* OR factor* OR imbalanc* OR issue* OR both OR specific* OR disparit* OR dependen* OR gap OR gaps OR influenc* OR discrepan* OR distribut* OR composition* OR variability OR comparison* OR accept* OR barrier* OR perception* OR perceiv* OR between* OR treat* OR alirocumab OR evolocumab OR statin OR atorvastatin OR rosuvastatin OR simvastatin OR ezetimibe OR ezetimib OR niacin OR enduracin OR nicamin OR nicobid OR nicocap OR nicolar OR nicotinate OR nicotinic OR bile-acid-sequestrant OR bempedoic-acid OR lomitapide OR mipomersen OR apheresis OR pcsk9-inhibitor OR anticholesteremic* OR hypocholesteremic* OR hmg-coa OR hydroxymethylglutaryl OR hydroxymethylglutaryl-coa OR hydroxymethylglutaryl-coenzyme OR (cholesterol W/2 inhibitor*)))) OR (TITLE-ABS-KEY ((men OR men's) W/2 (women*))) OR (TITLE-ABS-KEY (gender*-related OR gender*-based)))	1053

Original search for PsycInfo (OVID)

#	Searches	Results
1	((familia* or type* 2 or type* 2s or type* ii or type iis or type* iia* or type* iib* or essential* or autosomal dominant or genetic*) adj3 (hypercholesterolemi* or hypercholesterolaemi* or hyperlipoproteinemi* or hyperlipoproteinaemi* or hyper-cholesterolemi* or hyper-cholesterolaemi* or hyper-lipoproteinemi* or hyper-lipoproteinaemi* or apolipoprotein-b* or dyslipidemi*)).tw.	149
2	(HoFH or HFH or HzFH or HeFH or HHF).tw.	10
3	((extreme* or rare* or severe* or homozyg* or homo-zygo* or heterozygo* or hetero-zygo*) adj3 (hypercholesterolemi* or hypercholesterolaemi* or hyperlipoproteinemi* or hyperlipoproteinaemi* or hyper-cholesterolemi* or hyper-cholesterolaemi* or hyper-lipoproteinemi* or hyper-lipoproteinaemi* or apolipoprotein-b*)).tw.	15
4	lipoid gout*.tw.	0
5	(tendon* adj2 (xanthoma* or xanthogranulomatos*)).tw.	14
6	((heterozygo* or hetero-zygo*) adj2 FH).tw.	3
7	1 or 2 or 3 or 4 or 5 or 6	177
8	((sex* or gender* or man or men or male* or woman or women or female*) adj3 (difference* or different or characteristic* or ratio* or factor* or imbalanc* or issue* or both or specific* or disparit* or dependen* or gap or gaps or influenc* or discrepan* or distribut* or composition* or variability or comparison* or accept* or barrier* or perception* or perceiv* or between* or treat* or alirocumab or evolocumab or statin or atorvastatin or rosuvastatin or simvastatin or ezetimibe or ezetimib or niacin or enduracin or nicamin or nicobid or nicocap or nicolar or nicotinate or nicotinic or bile acid sequestrant or bempedoic acid or lomitapide or mipomersen or apheresis or PCSK9 inhibitor or anticholesteremic* or hypocholesteremic* or hmg-coa or hydroxymethylglutaryl or hydroxymethylglutaryl-coa or hydroxymethylglutaryl-coenzyme or (cholesterol adj2 inhibitor*))).tw.	242471
9	((men or men's) adj2 women*).tw.	49897
10	(gender*-related or gender*-based).tw.	5947
11	8 or 9 or 10	264683

12	7 and 11		18

Appendix 1.2. Supplementary Methods

Database strategies

The literature search was originally run July 21, 2020 and then was rerun prior to publication on May 10, 2022 and April 26, 2023. The following databases were searched for relevant studies: MEDLINE (via Ovid 1946 to 2020/07/21); Embase Classic + Embase (via Ovid 1947 to 2020/07/21); The Cochrane Central Register of Controlled Trials (via The Cochrane Library, Issue 7 of 12, January 2020); PubMed (National Library of Medicine 2020/07/14 – 2020/07/21); PsycInfo (via OVID 1987 – 2020/07/21) and Scopus (via Elsevier 1788 – 2020/07/21). The search strategies designed by a librarian used text words and relevant indexing to identify studies with FH patients and sex or gender differences in their diagnosis and treatment. The full MEDLINE strategy was applied to all databases, with modifications to search terms as necessary. No language limits were applied. Search strategies were peer-reviewed by two librarians.

Meta-analyses

Continuous outcomes

Where studies reported median values of LDL-C with interquartile ranges, we first assessed the skewness of the estimates and calculated means and standard deviations in instances where the data were not significantly skewed using validated methods. Where studies reported means and 95% confidence intervals, we estimated standard deviations by dividing the length of the confidence interval by 3.92, and then multiplying by the square root of the sample size:

$$SD = \sqrt{N} \times (upper\ limit - lower\ limit)/3.92$$

For absolute reductions in LDL-cholesterol (LDL-C), we first computed a difference in means between baseline and follow-up measurements reported for male and female subpopulations in included studies. We subsequently compared the mean reductions in LDL-C between men and women through random-effects meta-analysis, calculating a weighted mean difference with 95% confidence intervals.

Dichotomous outcomes

For dichotomous outcomes, where studies reported count data in separate subgroups (e.g., individuals with and without coronary artery disease), sums of the data were used in the meta-analyses. Where studies reported separate effect sizes for different subgroups, we calculated a pooled odds ratio with 95% CI using a fixed effects model, which was subsequently used in the meta-analysis. Where studies reported adjusted and unadjusted effect sizes, we used maximally adjusted estimates; hazard ratios, odds ratios, and relative risks were considered equivalent measures of risk.

Map

Using Microsoft Excel, we generated a world map with color indicators of country-level estimates of sex-differences in FH treatment. If more than 1 publication reported estimates for a given country, a random-effects pooled odds ratio and 95% CI was calculated and displayed.

Appendix 1.3. Supplementary References

Estimating the mean:

D. Luo, X. Wan, J. Liu and T. Tong* (2018), "Optimally estimating the sample mean from the sample size, median, mid-range and/or mid-quartile range", Statistical Methods in Medical Research, 27: 1785-1805.

Estimating the standard deviation:

X. Wan, W. Wang, J. Liu and T. Tong* (2014), "Estimating the sample mean and standard deviation from the sample size, median, range and/or interquartile range", BMC Medical Research Methodology, 14: 135

Appendix 2: Supplementary Tables and Figures

Supplementary Table 1. Characteristics of patients from the 133 studies included in the qualitative synthesis from the systematic review of sex differences in the treatment of familial hypercholesterolemia.

First author	Year	Country	Study design	Participants	FH Diagnosis	Recruitment period	Males n	Females n	Mean Age, y	On LLT	LLT details	Baseline LDL-C*	Treated LDL-C*	LDL-C % change	CVD outcomes (risk of MACE meta-analysis)	With baseline CVD (%)	Follow-up time	Included in risk of MACE or TRT Meta-Analyses
**Agarwala[1]**	2021	USA	Retrospective cohort	HeFH	DLCN<br>SB<br>MEDPED<br>AHA<br>Genetic	NR	295	513	NR	NR					ASCVD	NR	NR	
**Agarwala[2]**	2023	USA	Retrospective cohort	HeFH	DLCN<br>SB<br>MEDPED<br>AHA<br>Genetic	NR	280	502	M: 52±14<br>F: 55±15	Statins<br>M: 84.0%<br>F: 78.0%	Statins<br>Ezetimibe<br>PCSK9i	NR	M: 3.00±1.29<br>F: 3.23±1.24	NR	Premature ASCVD	M: 98 (35.0%)<br>F: 89 (17.7%)	NR	TRT<br>MACE
**Ahmad[3]**	2016	USA	Retrospective cohort	HeFH	Genetic	NR	42	51	M: 50±11<br>F: 51±13	NR					Premature CHD	M: 22 (52.4%)<br>F: 16 (31.4%)	NR	MACE
**Allard[4]**	2014	Canada	Retrospective cohort	HeFH	DLCN	1970-2014	180	229	CVD+: 66.6±13.5<br>CVD-: 58.6±15.2	CVD+: 33.3%<br>CVD-: 25.8%					CVD	M: 67 (37.2%)<br>F: 44 (19.2%)	NR	MACE
**Alonso[5]**	2008	Spain	Registry	HeFH	Genetic	NR	383	428	M: 45.5±13.3<br>F: 48.4±14.4	More than 80%					CVD	M: 116 (30.2%)<br>F: 62 (14.5%)	NR	
**Alonso[6]**	2014	Spain	Registry	HeFH	Genetic	NR	921	1039	Overall: 44.4±15.6	Approx. 90%					CVD	Overall: 12.6%	NR	MACE
**Alonso[7]**	2021	Spain	Registry	HeFH	Genetic	2016-2020	228	205	Median (IQR)<br>Overall:<br>55 (47-64)	M: 100%<br>F: 100%	PCSK9i	NR	NR	NR	-	Overall: 40.6%	Median (IQR)<br>2.5 years<br>(1.6–3.0)	
**Amrock[8]**	2017	USA	Cross-sectional analysis of registry data	HeFH<br>HoFH	SB<br>DLCN<br>MEDPED	2014-2016	1246	1921	Median (IQR)<br>M: 54 (42-63)<br>F: 59 (48-67)	Statins<br>M: 74.6%<br>F: 66.7%	Statins<br>Ezetimibe<br>Bile acid seq<br>Niacin<br>PCSK9i	M: 6.27±1.58<br>F: 6.31±1.48	M: 3.53±1.69<br>F: 3.96±1.86	M: -43.7%<br>F: -37.2%	-	M: 463 (37.2%)<br>F: 453 (23.6%)	NR	TRT
**Arnesen[9]**	2020	Norway	Retrospective cohort	HeFH	DLCN<br>Genetic	2006	144	129	NR	M: 96.6%<br>F: 89.8%	Statins<br>Ezetimibe<br>Resins<br>PCSK9i	NR	M: 2.8 (2.6-3.0)<br>F: 3.3 (3.0-3.5)	NR	-	Overall: 30.8%	Median 10.0 years	TRT
**Beaumont[10]**	1976	France	Cross-sectional	FH	Phenotypic	NR	158	116	M: 42±14<br>F: 49±18	NR					IVD: Angina, MI, PVD	IVD<br>M: 95 (60.1%)<br>F: 53 (45.7%)	NR	MACE
**Beheshti[11]**	2018	Denmark	Prospective	HeFH	SB<br>DLCN<br>MEDPED<br>Genetic	2003-	Possible FH: 1684	Possible FH: 2367	NR	NR					NR	NR	NR	
**Beliard[12]**	2014	France	Cross-sectional	HeFH	SB<br>DLCN<br>Genetic	1988-2011	826	843	Overall: 46.4±15.2	M: 89.4%<br>F: 79.1%	Statins<br>Ezetimibe	NR	NR	NR	-	Overall: 12.0%	NR	TRT

											Bile acid seq<br>Fibrate							
**Beliard**[13]	2018	France	Registry	HeFH	DLCN<br>Genetic	2015-2018	565	216	Overall: 60±13	Statins: 48.0%						Overall: 100%	NR	
**Benn**[14]	2012	Denmark	Cross-sectional	HeFH	DLCN<br>Genetic	1977-2011	204	298	Median (IQR)<br>Definite FH: 58 (46-64)<br>Probable FH: 59 (52-66)	M: 50.0%<br>F: 47.0%	Statins<br>Ezetimibe<br>Bile acid seq<br>other	NR	NR	NR	CAD	Definite/Probable FH: 291 (28.0%)	NR	TRT<br>MACE
**Berard**[15]	2019	France	Retrospective cohort	HeFH	DLCN	1995-2005	35	32	NR	70.1%					Premature ASCVD	Overall: 11.9%	NR	MACE
**Bertolini**[16]	2000	Italy	Retrospective cohort	HeFH	Phenotypic<br>Genetic	NR	132	172	NR	NR					CAD	M: 45 (34.1%)<br>F: 35 (20.3%)	NR	
**Bertolini**[17]	2013	Italy	Retrospective cohort	HeFH<br>HoFH	DLCN<br>Genetic	NR	818	951	M: 35.24±19.0<br>F: 38.14±20.0	NR					CHD	M: 212 (25.9%)<br>F: 130 (13.7%)	NR	MACE
**Besseling**[18]	2014	Netherlands	Registry	HeFH	Genetic	1994-2013	6848	7435	Overall: 38.3±21.2	37.7%					CVD	Overall: 9.2%	NR	MACE
**Bhatnagar**[19]	2000	UK	Retrospective cohort	HeFH	SB	1987-1998	183	197	NR	NR					Angina, MI, CABG, Stroke, CHD, CVD	CVD<br>M: 71 (38.8%)<br>F: 61 (31.0%)	NR	MACE
**Bogsrud**[20]	2019	Norway	Registry	HeFH	Genetic	2014-2015	307	407	Overall FU age: 44±16.3	Statins 89.0%	Statins<br>Ezetimibe<br>PCSK9i				MI, CHD	CHD<br>Overall: 15.1%	11.1±7.9 years	MACE
**Bowden**[21]	1994	Canada	Retrospective cohort	HeFH	Phenotypic	NR	48	67	Overall: 47.5±15.1	NR					CAD	M: 31 (64.0 %)<br>F: 24 (36.0%)	NR	MACE
**Braamskamp**[22]	2015	Netherlands	Clinical trial	HeFH	Phenotypic	1997-1999	94	111	Overall:<br>24±3.2	82.4%	Various type and dose of statins ± ezetimibe or cholestagel	NR	NR	NR	-	-	-	Clinical trial
**Budinsky**[23]	2001	Austria	Clinical trial	HeFH	Genetic	NR	7	8	M: 35.0±6.3<br>F: 31.6±6.3	100%	Pre-run 4 weeks dietary counseling then 250g/day of broiled edible pulp of prickly pear (opuntia robusta nutrient).	M: 5.88±0.48<br>F: 5.74±0.89	M: 5.39±0.36<br>F: 5.37±0.64	M: -8.33%<br>F: -6.45%	-	-	-	Clinical trial
**Carmena**[24]	1996	Canada	Retrospective cohort	HeFH	Phenotypic	NR	45	53	ASCVD+: 49 (29-73)<br>ASCVD-: 39 (18-64)	NR					ASCVD	M: 19 (42.2%)<br>F: 16 (30.2%)	NR	MACE
**Chan**[25]	2015	Australia	Cross-sectional	HeFH	Phenotypic<br>Genetic	2007-2014	171	219	46±16	67.0%					CAD	M: 41 (23.8%)<br>F: 23 (10.5%)	NR	MACE
**Chaves**[26]	2001	Spain	Clinical trial	HeFH	Genetic	NR	18	24	M: 43.0±16.4<br>F: 45.5±16.3	100%	Simvastatin 20 mg/day; Duration: 6 weeks	NR	NR	M: -35.0%<br>F: -33.0%	-	-	-	Clinical trial

**Coutinho**[27]	2021	Brazil	Registry	FH	Genetic	2011-2018	74	124	Median (IQR)<br>Overall: 66 (62-71)	88.5%					ASCVD	Overall: 40.9%		
**deGoma**[28]	2016	USA	Cross-sectional analysis of prospective registry data	HeFH	DLCN<br>SB<br>MEDPED<br>Genetic	NR	527	768	Overall: 57 (43-66)	83.7%	Per type of LLT Table 3	NR	NR	NR	-	Overall: 37.9%	NR	
**De Knijff**[29]	1990	Netherlands	Clinical trial	HeFH	Phenotypic	NR	75	45	M: 42.1 (18-65)<br>F: 51.0 (27-73)	100%	Simvastatin 40 mg/day; Duration: 12 weeks	M: 9.13±2.02<br>F: 9.72±2.42	M: 5.72±1.30<br>F: 5.59±1.32	M: -36.4%<br>F: -41.2%	-	-	-	Clinical trial
**De Sauvage Nolting**[30]	2003	Netherlands	Cross-sectional	HeFH	DLCN<br>Genetic	NR	287	229	Overall: 47.5±13.2	100%	Simvastatin 80mg				CVD	Overall: 37.2%	NR	MACE
**Doi**[31]	2021	Japan	Retrospective cohort	HeFH	Genetic<br>JAS	2005-2016	116	116	NR	NR					MI, revascularization	NR	NR	MACE
**Duell**[32]	2019	USA	Registry	HeFH	DLCN<br>Genetic<br>MEDPED<br>SB	NR	744	1156	Overall: 56.1±14.8	93.0%					ASCVD	M: 350 (47.0%)<br>F: 354 (30.6%)	20±11 months	MACE
**Ershova**[33]	2017	Russia	Retrospective cohort	HeFH	DLCN	2012-2013	7	23	57 (54-62)	Statins: 23.3%					CAD, MI	Overall: 40.0%	NR	MACE
**Ferrieres**[34]	1995	Canada	Retrospective cohort	HeFH	Genetic	NR	116	147	NR	NR					CAD	M: 54 (46.6%)<br>F:35 (23.8%)	NR	
**Firth**[35]	2008	South Africa	Retrospective cohort	HeFH	Phenotypic<br>Genetic	NR	488	541	NR	NR					Angina, MI, IHD, Stroke, TIA, PVD, Death	IHD<br>M: 251 (52.0%)<br>F: 187 (34.6%)	NR	MACE
**Galema-Boers**[36]	2014	Netherlands	Retrospective cohort	HeFH	Phenotypic<br>Genetic	2008-2009	152	169	Overall:<br>45.6±13.9	M: 100%<br>F: 100%	Statins	NR	NR	NR	-	Overall: 12.8%	NR	
**Gallo**[37]	2017	France	Prospective	HeFH	Genetic	2015	56	56	Median (IQR)<br>M: 44 (36-51)<br>F: 46.5 (38-55)	M: 94.6%<br>F: 69.6%	Statins<br>Ezetimibe	NR	NR	NR	-	NR	NR	TRT
**Hill**[38]	1990	Canada	Cross-sectional	HeFH	Phenotypic	NR	CAD data: 115	CAD data: 173	NR	NR					Angina, CAD, MI, Stroke	CAD<br>M: 47 (40.9%)<br>F: 187 (15.0%)	NR	MACE
**Hirobe**[39]	1982	Japan	Cross-sectional	HeFH	Phenotypic	NR	30	22	NR	NR					CAD	M: 17 (56.7%)<br>F: 6 (27.3%)	NR	MACE
**Holmes**[40]	2005	Canada	Retrospective cohort	HeFH	SB	NR	173	215	Overall:<br>53±16	NR					CVD	NR	NR	MACE
**Hoogerbrugge**[41]	1999	Netherlands	Clinical trial	HeFH	Phenotypic	NR	20	20	M: 43.0±11.0<br>F: 42.0±9.0	100%	Atorvastatin 40 mg/day for 6 weeks, then atorvastatin 80 mg/day for 6 weeks	M: 7.86±1.64<br>F: 8.28±2.27	Atorvastatin 40 mg/day<br>M: 4.50±1.40<br>F: 4.52±1.54<br>Atorvastatin 80 mg/day:<br>M: 3.88±1.17<br>F: 4.05±1.18	Atorvastatin 40 mg/day:<br>M: -43.0%<br>F: -45.0%<br>Atorvastatin 80 mg/day:<br>M: -51.0%<br>F: -50.0%	CAD	M: 4 (20.0%)<br>F: 0 (0%)	12 weeks	Clinical trial<br>MACE
**Hopkins**[42]	2001	USA	Registry	HeFH	MEDPED	NR	112	150	CVD+: 56±10.8<br>CVD-: 47.7±12.2	CVD+: 63.0%<br>CVD-: 37.0%					Premature CAD	M: 46 (41.1%)<br>F: 22 (14.7%)	NR	MACE
**Hovland**[43]	2019	Norway	Registry	HeFH<br>HoFH	Genetic	2001-2009	1472	1694	39.9±14.9	NR					Ischemic stroke	M: 9 (0.6%)<br>F: 9 (0.5%)	NR	
**Hu**[44]	2013	China	Retrospective cohort	HeFH	Phenotypic<br>MEDPED<br>Genetic	1990-2000	100	152	37±17	NR					CHD	NR	NR	

**Humphries**[45]	2018	UK	Retrospective cohort - registry	HeFH	DLCN SB	1980-2015	1724	1829	Median (IQR) M: 42.3 (32.2-52.3) F: 49.4 (33.3-59.8)	NR					CHD	M: 466 (27.0%) F: 342 (18.7%)	Up to 36 years	
**Iyen**[46]	2019	UK	Retrospective cohort - registry	FH	DLCN SB	1999-2016	6578	7519	Overall: 42.5±11.7	19.1%					CVD	NR	13.8 (8.4-17.7) years	MACE
**Iyen**[47]	2020	UK	Retrospective cohort - registry	HeFH	SB	1980-2010	1418	1570	M: 41.1±15.0 F: 46.1±16.8	NR					CHD	M: 352 (24.8%) F: 276 (17.6%)	Median (IQR) M: 17.9 (11.2-24.0) F: 18.1 (11.6-23.7) years	
**Iyen**[48]	2021	UK	Retrospective cohort - registry	HeFH	FH diagnosis code in care record	1988-2020	1191	1873	NR	100%	Statins	NR	NR	NR	-	NR	24 months	
**Jackson**[49]	2021	USA	Retrospective cohort	FH	USA ICD code for FH	2016-2019	25367	28431	NR	Statins M: 65.8% F: 59.6%	Statins Ezetimibe PCSK9i, Fenofibrat e Other	NR	NR	NR	-	Overall: 23.5%	NR	TRT
**Jansen**[50]	2004	Netherla nds	Retrospective cohort - registry	HeFH	DLCN MEDPED SB Genetic	1989-1999	1179	1221	CVD+: 56±11.4 CVD-: 46.4±12.7	95					CVD	M: 487 (41.3%) F: 295 (24.2%)	CVD+: 4.7 (2.4-9.0) years CVD-: 3.2 (1.2-6.5) years	MACE
**Jeenah**[51]	1993	South Africa	Clinical trial	HeFH	Genetic	NR	15	5	M: 44.1±7.3 F: 50.6±7.4	100%	Simvastatin 40 mg/day; Duration: 18 weeks	M: 7.8±0.1 F: 9.1±1.7	M: 5.2±0.67 F: 5.3±0.7	M: -31.1% F: -42.1%	-	-	-	Clinical trial
**Jerling**[52]	1997	South Africa	Clinical trial	HeFH	Phenotypic Genetic	NR	12	17	Median age: M: 37.5 [13.9] F: 42.7 [13.9]	100%	Simvastatin 10 mg/day 4 weeks, then simvastatin 20 mg/day 10 weeks	M: 7.54±1.78 F: 7.34±0.15	M: 4.50±1.51 F: 5.11±0.82	M: -36.2% F: -30.4%	-	-	-	Clinical trial
**Jiménez**[53]	2023	Spain	Retrospective cohort - registry	HeFH	DLCN Genetic	NR	1583	1778	Overall: 48.9±14.2	Statins: 84.1%	Statins PCSK9i	NR	NR	NR	-	Overall: 13.9%	4.2± 2.4	TRT
**Jung**[54]	2018	Korea	Retrospective cohort	HeFH	MEDPED	1994-2004	293	247	Overall: 45.8±13.0	NR					ASCVD	NR	14.6 years	
**Kane**[55]	1990	USA	Clinical trial	HeFH	Phenotypic Genetic	NR	18	22	M: 40.1±9.0 F: 42.2±14.0	100%	One or more of: colestipol up to 30 g/day, niacin up to 7.5 g/day, lovastatin 40 or 60 mg/day	M: 7.47±1.3 F: 7.19±1.6	M: 4.58±1.9 F: 4.34±1.4	M: -38.7% F: -39.6%	-	-	-	Clinical trial

											Duration: 26 months							
**Kastelein[56]**	2015	North America, Europe, and South Africa	Clinical trial	HeFH	DLCN SB Genetic	NR	180	143	Overall: FHI: 52.1±12.9 FH II: 53.2±12.9	100%	Alirocumab 75 mg Q2W or placebo; Duration: 78 weeks; endpoints measured at Week 24	NR	NR	Alirocumab M: -52.8% W: -43.9% Placebo M: +7.30% W: +6.70%	-	-	-	Clinical trial
**Khoury[57]**	2021	Canada	Bi-directional cohort	FH	SB Genetic	NR	891	888	NR	NR					CVE	M: 320 (35.9%) F: 138 (15.5%)	NR	MACE
**Kłosiewicz-Latoszek[58]**	2018	Poland	Retrospective cohort	HeFH	DLCN Genetic	1993-2016	63	159	At last FU M: 48.2±13.2 F: 57.9±16.4	Statins: 91.9%	Statins Ezetimibe Fibrate	M: 6.45±1.70 F: 6.17±1.16	M: 3.05±1.14 F: 2.97±0.78	M: -52.7% F: -51.9%	-	M: 21 (32.2%) F: 11 (17.5%)	M: 8.4±5.8 F: 7.4±5.3 years	
**Koeijvoets[59]**	2006	Netherlands	Retrospective cohort - registry	HeFH	DLCN Genetic	1989-2002	977	1047	NR	NR					CVD	M: 395 (40.4%) F: 247 (23.6%)	NR	
**Koeijvoets[60]**	2008	Netherlands	Retrospective cohort - registry	HeFH	DLCN Genetic	1989-2002	879	951	M: 48.8±0.4 F: 50.8±0.5	NR					CVD	M: 359 (40.8%) F: 224 (23.6%)	NR	
**Korneva[61]**	2019	Russia	Retrospective cohort - registry	HeFH	DLCN	NR	75	116	Median (IQR) Overall: 52.5 (39.0-60.0) High-dose statin: 58.0 (53.0-60.0) No statin use: 55.5 (36.0-62.0)	Statins Overall: 65.0% M: 58.7% F: 69.0%	Statins	NR	NR	NR	IHD	Overall: 35.6%	Duration LLT: 5.3±0.7 years	TRT
**Krogh[62]**	2016	Norway	Retrospective cohort - registry	FH	DLCN Genetic	1989-2010	47	32	Age at time of death: M: 60±11.6 F: 59±15.3	Statins: M: 93.0% F: 87.1%	Statins Ezetimibe Bile acid seq Niacin Other	M: 9.8±2.7 F: 9.4±2.0	M: 4.7±2.8 F: 5.3±2.0	M: -52.0% F: -43.6%	CVD	M: 47 (100%) F: 27 (84.4%)	NR	TRT
**Latkovskis[63]**	2018	Latvia	Retrospective cohort - registry	FH	DLCN	2015-2017	69	112	Overall: 51.3±14.1	54.1%					Premature CHD	M: 27 (39.1%) F: 37 (33.0%)		
**Leduc[64]**	2016	Canada	Cross-sectional cohort	HeFH	Genetic	NR	48	37	M: 45.5±9.9 F: 45.8±12.7	100%	Statins	M: 7.83±1.17 F: 7.58±1.48	M: 4.45±1.08 F: 4.24±0.93	M: -43.2% F: -44.1%	-	NR	NR	
**Leitersdorf[65]**	1994	Israel	Clinical trial	HeFH	Phenotypic Genetic	NR	35	28	NR	100%	Placebo 4 weeks then fluvastatin escalating dosage of 5, 10, 20 and 40 mg/day for 4 weeks	M: 7.3±1.5 F: 7.3±1.7	NR	M: -23.7% F: -24.3%	-	-	-	Clinical trial
**Li[66]**	2017	China	Retrospective cohort	FH	DLCN Genetic	2011-2016	162	119	CAD+: M: 47.9±9.7 F: 56.3±8.6	CAD+ M: 88.8% F: 78.6%	Statins	NR	NR	NR	CAD, Premature CAD	CAD M: 134 (82.7%) F: 84 (70.6%)	NR	TRT MACE

									CAD-:<br>M: 39.8±13.6<br>F: 50.8±16.9	CAD-<br>M: 64.3%<br>F: 57.1%								
**Mabuchi**[67]	1977	Japan	Cohort	HeFH	Phenotypic	NR	IHD data:<br>37	IHD data:<br>46	IHD data:<br>M: 42<br>F: 43	NR					IHD	Overall: 43.3%	NR	MACE
**Macedo**[68]	2008	Brazil	Cross-sectional	HeFH	DLCN	2003-2005	42	68	Overall: 48.9±16.2	NR					CHD	M: 18 (42.9%)<br>F: 12 (17.6%)	NR	
**Masana**[69]	2019	Spain	Prospective study	FH-phenotype	Phenotypic	2006-2008	5226	7597	ASCVD+: 67.7±10.7<br>ASCVD-: 60.5 ±13.6	ASCVD+:<br>97.4%<br>ASCVD-:<br>87.1%					ASCVD	M: 1394 (26.7%)<br>F: 808 (10.6%)	5 years	
**Mata**[70]	2002	Spain	Registry	FH	MEDPED<br>Genetic	1997-2000	370	449	M: 49.3±15<br>F: 45.8±13	NR					Premature CVD	M: 114 (30.8%)<br>F: 64 (14.3%)	NR	
**Matta**[71]	2021	Argentina	Prospective study	FH	DLCN	2015-2020	30	85	Overall: 56±10	Statins<br>M: 26.7%<br>F: 38.8%	Statins	NR	NR	NR	-	Overall: 5.4%	5 years	TRT
**Mattina**[72]	2019	France	Prospective study	FH	Genetic	2015-2016	70	84	M: 46.5±12.8<br>F: 49.8±13.7	M: 92.9%<br>F: 69.0%	Statins<br>Ezetimibe	NR	NR	NR	-	NR	NR	TRT
**Michikura**[73]	2017	Japan	Cross-sectional	HeFH	Phenotypic	2013-2016	53	77	Overall: 53.2±18.6	Statins:<br>83.0%					CAD	Overall: 26.0%	NR	MACE
**Miettinen**[74]	1988	Finland	Retrospective cohort	HeFH	Phenotypic	1968-1970	48	48	Overall: 55.9±1.6	NR					CAD, CAD Mortality	NR	15 years	MACE
**Miltiadous**[75]	2005	Greece	Clinical trial	HeFH	Genetic	NR	18	31	Overall: 44±15	100%	Atorvastatin 20 mg/day;<br>Duration:<br>12 weeks	M: 7.37±1.19<br>F: 7.68±1.31	M: 4.57±0.96<br>F: 4.61±0.98	M: -38.0%<br>F: -40.0%	-	-	-	Clinical trial
**Miname**[76]	2019	Brazil	Prospective study	HeFH	Genetic	NR	75	131	Overall: 45±14	68.9%					MACE	Overall: 7.2%	Median (IQR)<br>3.7 (2.7-6.8)<br>years	MACE
**Mohrschladt**[77]	2004	Netherlands	Retrospective cohort	FH	Phenotypic	NR	190	210	NR	86.2%					CVD	Overall: 32.8%	8 years	MACE
**Morisaki**[78]	1990	Japan	Clinical trial	HeFH	Phenotypic	NR	21	30	NR	100%	Probucol<br>1000<br>mg/day;<br>Duration:<br>average 10<br>months	NR	NR	NR	-	-	-	Clinical trial
**Mundal**[79]	2014	Norway	Registry	HeFH<br>HoFH	Genetic	1992-2010	59<br>LLT data:<br>38	54<br>LLT data:<br>30	Mean (range)<br>At inclusion:<br>M: 52.5 (12.2-81.1)<br>F: 57.4 (29.0-92.4)	88.2%	Statins ±<br>other LLT	NR	M: 4.4±1.4<br>F: 5.0±1.6	NR	CVD mortality	NR	19 years	TRT

**Mundal**[80]	2016	Norway	Registry	HeFH<br>HoFH	Genetic	1994-2009	2693	2845	At registration<br>M: 32.5±18.6<br>F: 35.0±19.4<br><br>First hospitalization<br>M: 44.9±15.2<br>F: 45.3±17.8	NR					CVD<br>hospitalizations	NR	Median (IQR)<br>5 (1-9) years	MACE
**Mundal**[81]	2018	Norway	Registry	HeFH<br>HoFH	Genetic	1992-2009	2062	2211	At registration<br>M: 31.2±18.1<br>F: 34.1±18.9	Statins<br>estimated<br>90.0%					Acute MI<br>CHD	NR	CHD<br>8 years	
**Neil**[82]	2003	UK	Prospective -<br>registry	HeFH	SB	1980-1998	1405	1466	NR	NR					CHD mortality	NR	12 754<br>person years	
**Neil**[83]	2004	UK	Cross-<br>sectional study<br>- registry	HeFH	SB	1980-1996	211<br>CAD+:104<br>CAD-:107	199<br>CAD+: 55<br>CAD-:144	CAD+<br>M: 56±10.2<br>F: 56.6±10.4<br>CAD-<br>M: 44.2±12.5<br>F: 44.8±14.4	CAD+<br>M: 99.0%<br>F: 94.5%<br>CAD-<br>M: 92.5%<br>F: 74.3%	Statins	NR	NR	NR	CAD	M: 104 (49.3%)<br>F: 55 (27.6%)	NR	TRT
**Neil**[84]	2008	UK	Cross-<br>sectional study<br>- registry	HeFH	SB	1980-2006	1650	1732	Median (IQR)<br>M: 43.1 (31.9-52.2)<br>F: 49.0 (32.7-59.7)	NR					Angina, CHD,<br>CVD mortality, MI	CHD<br>M: 462 (28.0%)<br>F: 338 (19.5%)	Median<br>M: 14.5 years<br>F: 14.1 years	MACE
**Nenseter**[85]	2011	Norway	Retrospective<br>cohort	HeFH	Genetic	2007-2009	68	44	Median (min-max)<br>CHD+: 53 (39-73)<br>CHD-: 58 (47-76)	Statins<br>CHD+:<br>98.3%<br>CHD-: 98.1%					CHD	NR	NR	MACE
**Nybo**[86]	2007	Denmar<br>k	Retrospective<br>cohort	HeFH	Phenotypic<br>Genetic	NR	438	600	NR	0%					CVD	Overall: 20.3%	NR	
**Panagiotakos**<br>[87]	2003	Greece	Prospective<br>cohort	HeFH	MEDPED	1987-1997	295	344	M: 41.7±12.1<br>F: 42.8±12.8	NR					CHD	Overall: 13.6%	15 years	MACE
**Pang**[88]	2018	South<br>Africa<br>Australia<br>Brazil	Retrospective<br>cohort -<br>registry	HeFH	Genetic	1990-2017	399	476	Overall: 45.1±14.2	59.2%					CAD	Overall: 24.5%	NR	MACE
**Pang**[89]	2021	Australia	Registry	FH	DLCN<br>Genetic	2015-2019	771	757	M: 52.9±14.8<br>F: 53.8±15.4	LLT<br>M: 84.3%<br>F: 77.3%	Statins<br>Ezetimibe<br>PCSK9i	M: 7.2±2.6<br>F: 7.5±2.4	M: 3.6±2.0<br>F: 4.1±2.0	M: -50.0%<br>F: -45.3%	CAD	M: 309 (40.1%)<br>F: 119 (15.7%)	NR	TRT
**Paquette**[90]	2017	Canada	Cross-<br>sectional study	HeFH	Genetic	NR	288	382	CVD+: 49.9±12.1<br>CVD-: 35.4±12.1	CVD+: 79.0%<br>CVD-: 72.0%					CVD	M: 123 (42.7%)<br>F: 107 (28.0%)	NR	
**Paquette**[91]	2017	Canada	Retrospective<br>cohort	HeFH	Genetic	NR	354	364	CVD+: 49.3±10.3<br>CVD-: 38.3±12.6	NR					CVD	M: 182 (51.4%)<br>F: 84 (23.1%)	NR	
**Paquette**[92]	2021	Canada<br>France<br>UK	Prospective<br>cohort -<br>registry	HeFH	DLCN<br>Genetic	NR	1748	2138	Overall: 43±13	46.8%					ASCVD	M: 247 (14.1%)<br>F: 136 (6.4%)	8 ± 9 years	
**Pasta**[93]	2020	Italy	Retrospective<br>cohort	HeFH	Genetic	1989-2019	130	164	M: 52±20<br>F: 52±19	87.8%					ASCVD	M: 57 (43.8%)<br>F: 34 (20.7%)	Median (IQR)<br>13 (5-21)<br>years	
**Perak**[94]	2016	USA	Retrospective<br>cohort	HeFH	AHA	1999-2010	1559	2291	NR	NR					ASCVD, CHD	NR	≥10 years	MACE
**Pereira**[95]	2014	Brazil	Cross-<br>sectional	HeFH	DLCN<br>Genetic	2009-2013	71	131	CVD+: 57.6±10.2<br>CVD-: 48.2±14.9	NR					CVD	M: 29 (40.8%)<br>F: 28 (21.4%)	NR	
**Pereira**[96]	2015	Brazil	Cross-<br>sectional	HeFH	DLCN<br>Genetic	2009-2013	71	131	Overall: 50.8±14.4	NR					CVD	Overall: 28.2%	NR	

**Pérez-Calahorra**[97]	2017	Spain	Cross-sectional analysis of registry data	HeFH	DLCN	2013-2016	851	881	Median (IQR) Overall: 52 (41-61)	NR	NR	NR	NR	NR	CVD	M: 161 (18.9%) F: 65 (7.4%)	NR	TRT MACE
**Perez de Isla**[98]	2016	Spain	Registry	HeFH	Genetic	2004-2013	1264	1488	Median (IQR) Overall: 49.5 (28.0-61.0)	On max LLT: 71.8%	Statins Ezetimibe Fibrates Bile acid seq	NR	NR	NR	-	Overall: 13.0%	5.1±3.1 years	
**Perez de Isla**[99]	2017	Spain	Registry	HeFH	Genetic	2004-2015	1087	1317	Overall: 45.5±15.4	At entry: 84.2%					ASCVD	At entry Overall: 12.8%	5.5±3.2 years	MACE
**Perez Garcia**[100]	2018	Spain	Retrospective cohort	HeFH HoFH	Genetic	2001-2017	67	66	Overall: 45.3±16.5	NR					CHD	M: 9 (13.4%) F: 2 (3.0%)		MACE
**Pijlman**[101]	2010	Netherlands	Cross-sectional	HeFH	Phenotypic Genetic	NR	587	662	Overall: 49	Statins: 96%	Statins Ezetimibe Fibrates Niacin Bile acid seq	NR	NR	NR	-	Overall: 17.0%	NR	
**Pisciotta**[102]	2005	Italy	Prospective cohort	HeFH	Phenotypic	NR	103	146	M: 51.9±10.5 F: 58.5±7.5	0%					CAD	M: 45 (43.7%) F: 54 (37.0%)	NR	MACE
**Pitsavos**[103]	2004	Greece USA	Retrospective cohort	HeFH	Phenotypic	1987-2002	295	344	M: 41±12 F: 43±13	CHD+: 74.7% CHD-: 70.0%					CHD	M: 53 (18.0%) F: 34 (9.9%)	6±3 years	MACE
**Raal**[104]	2015	International	Clinical trial	HeFH	SB	2013	Evo 140mg: 66 Evo 420mg :64	Evo 140mg: 44 Evo 420mg :46	Overall Evo 140mg: 52.6±12.3 Evo 420mg: 51.9±12.0	100%	Evolocumab 140mg Q2W or 420mg Q4W or placebo; Duration: 12 weeks	NR	NR	Evolocumab 140mg Q2W M: -64.8% F: -53.5% 420mg Q4W M: -58.0% F: -65.0%	-	-	-	Clinical trial
**Ramos**[105]	2020	Spain	Retrospective cohort	FH-phenotype	Phenotypic	2006-2013	3047	4385	60.5±13.6	87.1%					ASCVD	Derivation dataset Overall: 0%	NR	MACE
**Razek**[106]	2018	Canada	Retrospective longitudinal study using registry data	FH	DLCN Genetic	2015-2017	121	154	NR	PCSK9i M: 19.0% F: 16.2%	Statins Ezetimibe PCSK9i	NR	NR	NR	-	NR	NR	
**Roy**[107]	2022	Canada	Retrospective longitudinal study using registry data	HeFH	Genetic	NR	281	334	Overall: 42.4±12.9	NR	Statins	NR	NR	NR	-	NR	NR	
**Ryzhaya**[108]	2021	Canada	Retrospective longitudinal study using registry data	FH	DLCN	NR	275	304	M: 43.9±14.1 F: 46.6±15.8	Statins M: 89.6% F: 88.4%	Statins Ezetimibe PCSK9i	M: 6.93±2.0 F: 6.83±2.2	M: 2.71±1.7 F: 3.09±1.2	M: -60.9% F: -54.8%	-	M: 57 (20.7%) F: 30 (9.9%)	8 years	TRT
**Sánchez-Ramos**[109]	2021	Spain	Prospective cohort	HeFH	Phenotypic	2004-2007	602	105	37.2±10.5	7.8%					MACE	CVD Overall: 3.3%)	6.6±3.6 years	MACE
**Schreuder**[110]	2023	Netherlands Norway	Cross-sectional study	HeFH	DLCN Genetic	2011-2017	1465	1713	Median (IQR) M: 47.4 (36.9-58.1) F: 48.7 (33.0-61.0)	Per type of LLT Table 1	Statins Ezetimibe PCSK9i	M: 6.0±1.7 F: 6.2±1.6	M: 2.8±0.9 F: 3.1±1.0	M: -52.2% F: -50.5%	-	M: 275 (18.8%) F: 221 (12.9%)	NR	TRT
**Seed**[111]	1990	UK	Retrospective cohort	HeFH	SB	NR	61	54	CHD+: 47.6±9.0 CHD-: 42.0±13.8	NR					CHD	M: 35 (57.4%) F: 19 (35.2%)	12 months	MACE
**Silva**[112]	2016	Brazil	Prospective cohort	FH	Genetic	NR	302	516	NR	NR					CVD	NR	1 year	MACE

**Simon Broome†**[113]	1991	UK	Registry	HeFH	SB	1980-1989	282	244	NR	NR					CVD	MI<br>M: 42 (14.9%)<br>F: 19 (7.8%)	10 years	
**Simon Broome†**[114]	1999	UK	Registry	HeFH	SB	1980-1995	605	580	Median<br>M: 40.3<br>F: 43.9	NR					MI	M: 83 (13.7%)<br>F: 38 (6.6%)	M: 7.8 years<br>F: 6.9 years	
**Simonen**[115]	1987	Finland	Retrospective cohort	HeFH	Phenotypic	1970's	49	48	M: 39.2±1.5<br>F: 46.8±1.8	NR					Angina, CAD	CAD<br>M: 40 (81.6%)<br>F: 28 (58.3%)	NR	MACE
**Sinzinger**[116]	1994	Austria	Clinical trial	SH	Phenotypic	NR	352	363	M: 53.6±10.3<br>F: 58.6±11.7	100%	Pravastatin 10 mg/day then pravastatin 20 mg/day 4 weeks if LDL-C <10%, then pravastatin 40 mg/day 4 weeks if LDL-C <10%. Duration: 12 weeks	M: 6.09±1.07<br>F: 6.15±1.14	M: 3.62±0.86<br>F: 4.20±0.95	M: -40.6%<br>F: -31.7%	-	-	-	Clinical trial
**Slack**[117]	1969	UK	Retrospective cohort	HeFH	Phenotypic	NR	51	53	NR	NR					IHD, IHD mortality	M: 35 (68.6%)<br>F: 29 (54.7%)	NR	MACE
**Smilde**[118]	2000	Netherlands	Clinical trial	FH	Phenotypic<br>Genetic	NR	16	29	Overall: 46±10	100%	Various type and dose of statins: simva/atorva from 40 to 80 mg/day	NR	NR	NR	-	-	-	Clinical trial
**Stein**[119]	2012	Canada<br>USA	Clinical trial	HeFH	SB<br>Genetic	2008-2010	50	33	Overall: 56.2±9.7	100%	Mipomersen 200 mg per week; Duration: 26 weeks	M: 3.77±0.92<br>F: 4.25±1.64	M: 2.88±0.83<br>F: 2.39±0.81	M: -19.97%<br>F: -40.60%	-	-	-	Clinical trial
**Svendsen**[120]	2021	Norway	Prospective cohort	FH	Genetic	1992-2014	123	67	NR	NR					Acute MI	Overall: 100%	17 years	
**Tada**[121]	2021	Japan	Retrospective cohort	HeFH<br>HoFH	JAS<br>Genetic	1994-2019	490	560	Overall: 48±18	Statins: 98.2%					MACE	Overall: 10.8%	Median (IQR) 12.3 (9.1-17.5) years	
**Tada**[122]	2023	Japan	Retrospective cohort	HeFH<br>HoFH	JAS<br>Genetic	2000-2020	490	560	Overall: 49±16	Statins: 97.6%					MACE	M: 82 (16.7%)<br>F: 50 (8.9%)	12.6 (9.1-17.4) years	MACE
**Vallejo-Vaz**[123]	2018	UK	Retrospective study	HeFH	Phenotypic	NR	714	626	NR	M: 65.5%<br>F: 65.5%	PCSK9i – Aliro 75/150mg	In mg/dL<br>M: 150.8±54.1<br>F: 159.6±62.5	NR	NR	-	M: 1.3%<br>F: 0.2%	78 weeks	TRT
**Vallejo-Vaz**[124]	2021	International‡	Retrospective cross-sectional - registry	HeFH	DLCN<br>Genetic<br>MEDPED<br>SB<br>Canadian<br>JAS	NR	19031	21999	Median (IQR)<br>M: 44.9 (34.0-55.8)<br>F: 48.2 (35.0-60.0)	M: 61.1%<br>F: 58.4%	Statins<br>Ezetimibe<br>Fibrates<br>PCSK9i	NR	Median (IQR)<br>M: 4.18 (3.16-5.51)<br>F: 4.26 (3.24-5.75)	NR	CAD, PAD, Premature CAD, Stroke	Overall: 17.4%	NR	TRT<br>MACE

**Versmissen**[125]	2008	Netherlands	Retrospective cohort - registry	HeFH	DLCN<br>Genetic	1990-2002	924	1026	NR	Statins<br>M: 21.3%<br>F: 21.0%					MI	NR	8.5 years	
**Vlad**[126]	2021	Romania	Prospective cohort	FH	SB<br>DLCN<br>MEDPED	2016-2017	22	39	M: 46.1±1<br>F: 49.7±1	Statins monotherapy at registration<br>M: 39.8%<br>F: 38.5%	Statins<br>Ezetimibe<br>Fibrates	NR	NR	NR	ASCVD, CHD, PAD, Stroke	At registration:<br>M: 6 (27.3%)<br>F: 22 (56.4%)	2 years	TRT<br>MACE
**Vuorio**[127]	1997	Finland	Registry	HeFH	Phenotypic<br>Genetic	1992-1996	73	106	NR	NR					CHD, MI	CHD<br>M: 26 (35.6%)<br>F: 29 (27.4%)	NR	MACE
**Waluś-Miarka**[128]	2017	Poland	Prospective cohort	FH	SB<br>Genetic	2011-2013	63	91	M: 44.3±12.1<br>F: 48.2±14.0	NR	NR	NR	NR	NR	-	NR	NR	TRT
**Wierzbicki**[129]	2000	UK	Retrospective cohort	HeFH	SB	NR	66	46	CHD+: 54.3±11.6<br>CHD-: 50.1±13.9	NR					CHD	M: 21 (31.8%)<br>F: 8 (17.4%)	≥6 years	MACE
**Yaman**[130]	2020	Turkey	Cross-sectional	HeFH	DLCN	2010-2016	119	248	Overall: 50.5±11.7	42.0%					CHD	Overall: 19.9%	NR	MACE
**Zamora**[131]	2017	Spain	Cross-sectional	FH-phenotype	Phenotypic	2006-2014	6747	7952	Overall: 61.5±15.	86.5%	Statins<br>Ezetimibe	NR	NR	NR	-	Overall: 17.5%<br>M: 1659 (24.6%)<br>F: 919 (11.6%)	NR	
**Zamora**[132]	2023	Spain	Cross-sectional	FH-phenotype	Phenotypic	2006-2014	6747<br>CAD+:1659<br>CAD-: 5088	7952<br>CAD+: 919<br>CAD-: 7033	CAD+<br>M: 67±11<br>F: 73±11.4<br>CAD-<br>M: 58±16<br>F: 63±15.4	Per type of statin Table 2	Statins<br>Ezetimibe	Overall<br>M: 7.35±0.8<br>F: 7.35±0.9<br>CAD+<br>M: 7.44±0.90<br>F: 7.44±0.93<br>CAD-<br>M: 7.31±0.98<br>F: 7.34±0.95	Overall<br>M: 4.23±1.3<br>F: 4.50±1.6<br>CAD+<br>M: 3.41 ± 1.24<br>F: 3.77 ± 1.29<br>CAD-<br>M: 4.62 ± 1.5<br>F: 4.65 ± 1.6	Overall<br>M: 42.4%<br>F: 38.6%<br>CAD+<br>M: -54.2%<br>F: -49.3%<br>CAD-<br>M: -36.8%<br>F: -36.6%	-	M: 1659 (24.6%)<br>F: 919 (11.6%)	NR	TRT
**Zhao**[133]	2019	Canada	Bi-directional cohort	FH	Canadian<br>Genetic	NR	80	102	M: 44.9±14.5<br>F: 50.7±17.0	M: 10.0%<br>F: 6.9%	NR	M: 6.04±1.2<br>F: 6.35±1.53	M: 3.11±1.41<br>F: 3.30±1.48	M: -48.5%<br>F: -48.0%	Premature MI	M: 18 (22.5%)<br>F: 10 (9.8%)	≤1 year	TRT<br>MACE

*Values are mean ± standard and units in mmol/L unless otherwise stated
†Scientific Steering Committee on behalf of the Simon Broome Register Group.
‡56 countries (of 66) participating in the European Atherosclerosis Society's Familial Hypercholesterolemia Studies Collaboration.

Abbreviations: AHA - American Heart Association; ASCVD - atherosclerotic cardiovascular disease; Bile acid seq. – bile acid sequestrants; CABG – coronary artery bypass graft surgery; CAD - coronary artery disease; CHD - coronary heart disease; CVD - cardiovascular disease; CVE – cardiovascular event; DLCN - Dutch Lipid Clinic Network; Evo – evolocumab; F - females; FH - familial hypercholesterolemia; FU – follow-up; HeFH - heterozygous familial hypercholesterolemia; HoFH - homozygous familial hypercholesterolemia; ICD code - International Classification of Diseases; IHD – ischemic heart disease; IQR: interquartile range; IVD - Ischemic vascular disease; JAS - Japanese Atherosclerosis Society; LDL-C - low-density lipoprotein cholesterol; LLT - lipid lowering therapy; M - males; MACE – major adverse cardiac events; MEDPED – Making Early Diagnosis to Prevent Early Deaths; mg – milligrams; MI - myocardial infarction; NR - not reported; PCSK9i – proprotein convertase subtilisin/kexin type 9 inhibitor; PAD - peripheral arterial disease; PVD - peripheral vascular disease; SB – Simon Broome; SH – severe hypercholesterolemia; TRT – treatment; UK - United Kingdom; USA - United States of America.

Supplementary Table 2. Estimates of sex differences in treatment with lipid lowering therapy by country.

Country	Studies	Population	I^2, %	Odds Ratio (95% CI)
Argentina	1	115		1.75 [0.70, 4.37]
Australia	1	1,528		0.63 [0.49, 0.81]
Canada	2	761	0	0.58 [0.38, 0.89]
China	1	281		0.48 [0.26, 0.89]
Denmark	1	502		0.89 [0.62, 1.28]
France	3	1,935	70	0.25 [0.10, 0.59]
Norway	3	416	0	0.37 [0.17, 0.80]
Poland	1	154		1.38 [0.72, 2.64]
Romania	1	61		1.34 [0.44, 4.08]
Russia	1	191		1.57 [0.86, 2.87]
Spain	3	19,792	89	0.99 [0.70, 1.40]
United Kingdom	2	1,750	95	0.46 [0.10, 2.24]
USA	3	57,747	54	0.77 [0.68, 0.87]

Odds ratios are random-effects estimates.

Supplementary Table 3. Random-effects summary estimates of sex differences in treatment with lipid lowering therapy, with exclusion of EAS FHSC study data, Vallejo-Vaz et al[124].

Analysis	Sensitivity estimate (95% CI)	Primary analysis (95% CI)*
**Use of any lipid lowering therapy**	0.71 [0.60, 0.84]	0.76 [0.67, 0.87]
**Medication class**		
Statins	0.77 [0.65, 0.92]	0.79 [0.69, 0.92]
High-intensity statins	0.64 [0.54, 0.75]	0.66 [0.57, 0.76]
Ezetimibe	0.65 [0.59, 0.73]	0.67 [0.57, 0.78]
PCSK9 Inhibitors	0.70 [0.50, 0.97]	0.70 [0.54, 0.91]
**Combination therapy**		
2+ agents	0.60 [0.46, 0.78]	0.82 [0.79, 0.86]

Values are odds ratios (95% confidence intervals), derived under random effects models.
*male sex as a reference;
Abbreviations: EAS – European Atherosclerosis Society; FHSC – Familial Hypercholesterolemia Studies Collaboration; PCSK9 – proprotein convertase subtilisin/kexin type-9.

Supplementary Table 4. Fixed- and random-effects estimates of sex differences in treatment, cholesterol reduction, and target attainment.

Outcome	Studies	Fixed-effects estimate (95% CI)	Random-effects estimate (95% CI)
**Use of any lipid lowering therapy**	25	0.87 [0.85, 0.89]	0.74 [0.66, 0.85]
**Medication class**			
Statins	19	0.82 [0.80, 0.84]	0.79 [0.69, 0.92]
High-intensity statins	11	0.69 [0.67, 0.72]	0.66 [0.57, 0.76]
Ezetimibe	11	0.81 [0.78, 0.84]	0.67 [0.57, 0.78]
PCSK9 Inhibitors	8	0.85 [0.79, 0.91]	0.70 [0.54, 0.91]
**Combination therapy**			
Statins + ezetimibe	3	0.67 [0.58, 0.77]	0.64 [0.48, 0.86]
2 or more agents	5	0.82 [0.79, 0.86]	0.67 [0.53, 0.84]
**Cholesterol reduction targets**			
≥ 50% reduction in LDL-C	5	0.59 [0.55, 0.62]	0.78 [0.54, 1.13]
LDL-C < 2.5 mmol/L	5	0.96 [0.95, 0.97]	0.85 [0.74, 0.97]
LDL-C < 1.8 mmol/L	4	1.00 [0.99, 1.01]	0.64 [0.43, 0.97]
**Mean difference in cholesterol reduction***			
Absolute LDL-C reductions	10	-0.22 [-0.27, -0.17]	-0.18 [-0.32, -0.05]
Relative LDL-C reductions	10	-4.04 [-4.69, -3.38]	-3.42 [-5.10, -1.66]

Values are odds ratios (95% confidence intervals) unless otherwise specified. All models used inverse variance weighting.
*****Absolute LDL-C reductions in mmol/L; relative LDL-C reductions from baseline measurements in %.
Abbreviations: LDL-C – low-density lipoprotein cholesterol; mmol/L – millimoles per litre; PCSK9 – proprotein convertase subtilisin/kexin type-9.

Supplementary Figure 1. Absolute LDL-cholesterol reductions (mmol/L) in males and females in clinical trials included systematic review of sex differences in treatment of familial hypercholesterolemia. Figure depicts difference in means of LDL-cholesterol from baseline to follow-up measurements reported in clinical trials. Squares represent mean differences; horizontal lines show 95% confidence intervals (CI). Area of the square is proportional to the inverse variance of the estimate. Diamonds represent pooled estimates with 95% CIs derived under the random effects model. Solid vertical line indicates null effect. Test of subgroup differences refers to variations in the difference of means between male and female subgroups; p-values < 0.05 are considered significant.

	Baseline			Treated				Mean Difference
**Study or Subgroup**	**Mean**	**SD**	**Total**	**Mean**	**SD**	**Total**	**Weight**	**IV, Random, 95% CI**
**4.1.1 Males**								
Budinsky (2000)	5.88	0.48	7	5.39	0.36	7	12.1%	0.49 [0.05, 0.93]
De Knijff (1990)	9.13	2.02	71	5.72	1.3	71	11.7%	3.41 [2.85, 3.97]
Hoogerbrugge (1999)	7.86	1.64	20	3.88	1.17	20	10.1%	3.98 [3.10, 4.86]
Jeenah (1993)	7.8	0.1	15	5.2	0.67	15	12.5%	2.60 [2.26, 2.94]
Jerling (1997)	7.54	1.78	12	4.5	1.51	12	7.9%	3.04 [1.72, 4.36]
Kane (1990)	7.47	1.3	18	4.58	1.9	18	9.2%	2.89 [1.83, 3.95]
Miltiadous (2005)	7.37	1.19	18	4.57	0.96	18	11.0%	2.80 [2.09, 3.51]
Sinzinger (1994)	6.09	1.07	352	3.62	0.86	352	12.9%	2.47 [2.33, 2.61]
Stein (2012)	3.77	0.92	50	2.39	0.81	50	12.5%	1.38 [1.04, 1.72]
**Subtotal (95% CI)**			**563**			**563**	**100.0%**	**2.49 [1.90, 3.09]**
Heterogeneity: $Tau^2 = 0.70$; $Chi^2 = 133.50$, df = 8 ($P < 0.00001$); $I^2 = 94\%$								
Test for overall effect: Z = 8.23 ($P < 0.00001$)								
**4.1.2 Females**								
Budinsky (2000)	5.74	0.89	8	5.37	0.64	8	11.3%	0.37 [−0.39, 1.13]
De Knijff (1990)	9.73	2.42	43	5.59	1.32	43	10.9%	4.14 [3.32, 4.96]
Hoogerbrugge (1999)	8.28	2.27	20	4.05	1.18	20	9.2%	4.23 [3.11, 5.35]
Jeenah (1993)	9.1	1.7	5	5.3	0.7	5	6.8%	3.80 [2.19, 5.41]
Jerling (1997)	7.34	0.15	17	5.11	0.825	17	13.1%	2.23 [1.83, 2.63]
Kane (1990)	7.19	1.6	22	4.34	1.4	22	10.6%	2.85 [1.96, 3.74]
Miltiadous (2005)	7.68	1.31	31	4.61	0.98	31	12.3%	3.07 [2.49, 3.65]
Sinzinger (1994)	6.15	1.14	363	4.2	0.95	363	13.8%	1.95 [1.80, 2.10]
Stein (2012)	4.25	1.64	33	2.39	0.81	33	12.0%	1.86 [1.24, 2.48]
**Subtotal (95% CI)**			**542**			**542**	**100.0%**	**2.61 [2.02, 3.19]**
Heterogeneity: $Tau^2 = 0.65$; $Chi^2 = 79.93$, df = 8 ($P < 0.00001$); $I^2 = 90\%$								
Test for overall effect: Z = 8.68 ($P < 0.00001$)								

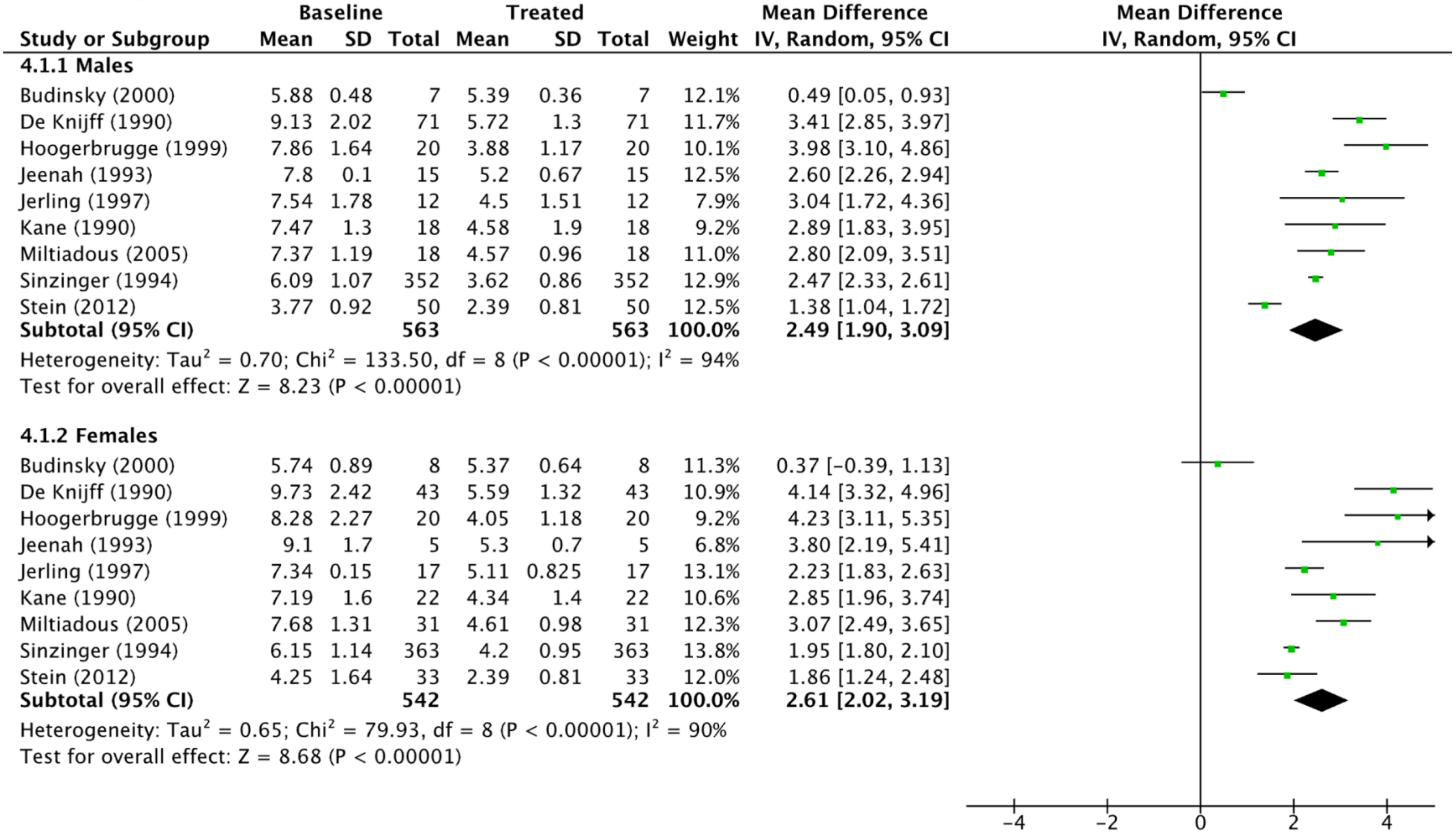

Test for subgroup differences: $Chi^2 = 0.07$, df = 1 ($P = 0.79$), $I^2 = 0\%$

Supplementary Figure 2. Sex differences in LDL-cholesterol reductions in males and females in clinical trials included systematic review of sex differences in treatment of familial hypercholesterolemia. Panel A depicts sex differences in mean LDL-cholesterol reduction (mmol/L) reported in clinical trials. Panel B depicts sex differences in mean LDL-cholesterol reduction (%) from baseline levels reported in clinical trials.

A

	Females			Males				Mean Difference
Study or Subgroup	Mean	SD	Total	Mean	SD	Total	Weight	IV, Random, 95% CI
Budinsky (2000)	0.39	0.8851	8	0.49	0.4758	7	13.5%	−0.10 [−0.81, 0.61]
De Knijff (1990)	4.14	2.6645	43	3.41	2.3659	71	10.4%	0.73 [−0.24, 1.70]
Hoogerbrugge (1999)	4.23	2.3931	20	3.98	1.8803	20	7.3%	0.25 [−1.08, 1.58]
Jeenah (1993)	3.8	1.2966	5	2.6	0.614	15	8.5%	1.20 [0.02, 2.38]
Jerling (1997)	2.23	0.778	17	3.04	2.0775	12	8.0%	−0.81 [−2.04, 0.42]
Kane (1990)	2.85	2.0073	22	2.89	2.1316	18	7.6%	−0.04 [−1.33, 1.25]
Miltiadous (2005)	3.07	1.5812	31	2.8	1.4277	18	11.6%	0.27 [−0.59, 1.13]
Sinzinger (1994)	1.95	1.4533	363	2.47	1.3355	352	19.3%	−0.52 [−0.72, −0.32]
Stein (2012)	1.86	1.7485	33	1.38	1.1964	50	13.8%	0.48 [−0.20, 1.16]
**Total (95% CI)**			**542**			**563**	**100.0%**	**0.11 [−0.34, 0.56]**

Heterogeneity: $Tau^2 = 0.26$; $Chi^2 = 23.49$, df = 8 (P = 0.003); $I^2 = 66\%$
Test for overall effect: Z = 0.48 (P = 0.63)

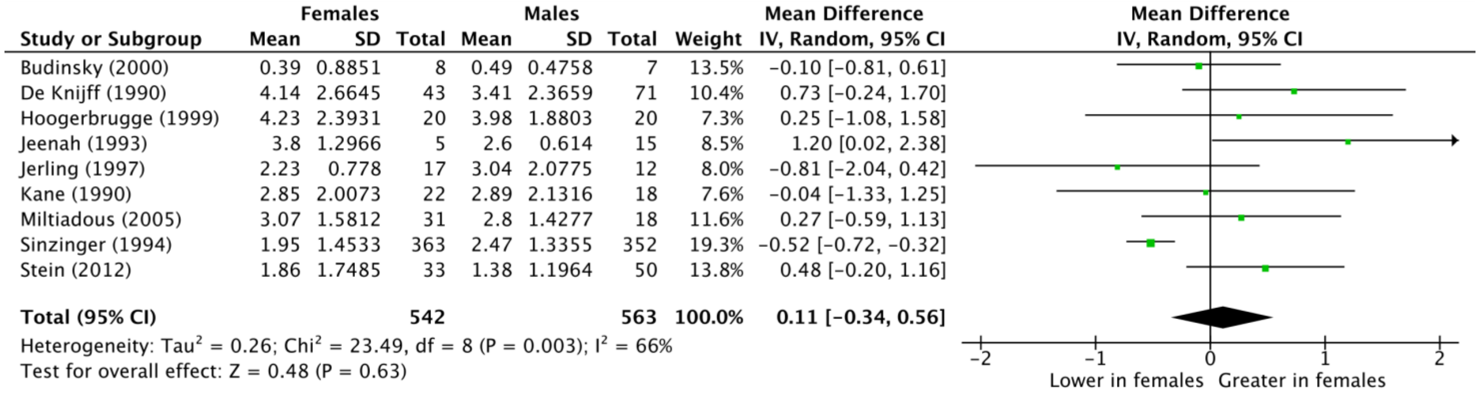

B

	Females			Males				Mean Difference
Study or Subgroup	Mean	SD	Total	Mean	SD	Total	Weight	IV, Random, 95% CI
Chavez (2001)	33	13.0014	24	35	13.9959	18	7.1%	−2.00 [−10.30, 6.30]
De Knijff (1990)	41.2	11.6976	43	36.4	11.407	71	10.0%	4.80 [0.41, 9.19]
Hoogerbrugge (1999)	50	12.0082	20	51	0.8974	20	9.3%	−1.00 [−6.28, 4.28]
Jeenah (1993)	42.1	6.7973	5	31.1	10.7985	15	7.2%	11.00 [2.92, 19.08]
Jerling (1997)	30.4	10.7361	17	36.2	9.7424	12	7.6%	−5.80 [−13.31, 1.71]
Kane (1990)	39.6	11.7282	22	38.7	9.7529	18	8.3%	0.90 [−5.76, 7.56]
Kastelein (2015)	43.9	1.8148	143	52.8	1.6997	180	12.0%	−8.90 [−9.29, −8.51]
Leitersdorf (1994)	24.3	11.8888	28	23.7	9.4029	35	9.3%	0.60 [−4.79, 5.99]
Miltiadous (2005)	40	10.9868	31	38	10.9997	18	8.5%	2.00 [−4.39, 8.39]
Raal (2015)	65	47.1439	46	58	34.0282	64	3.3%	7.00 [−8.97, 22.97]
Sinzinger (1994)	31.7	10.7541	363	40	9.7302	352	11.7%	−8.30 [−9.80, −6.80]
Stein (2012)	40.6	20.5311	33	19.97	27.7273	50	5.7%	20.63 [10.23, 31.03]
**Total (95% CI)**			**775**			**853**	**100.0%**	**0.27 [−3.17, 3.71]**

Heterogeneity: $Tau^2 = 25.72$; $Chi^2 = 135.13$, df = 11 (P < 0.00001); $I^2 = 92\%$
Test for overall effect: Z = 0.16 (P = 0.88)

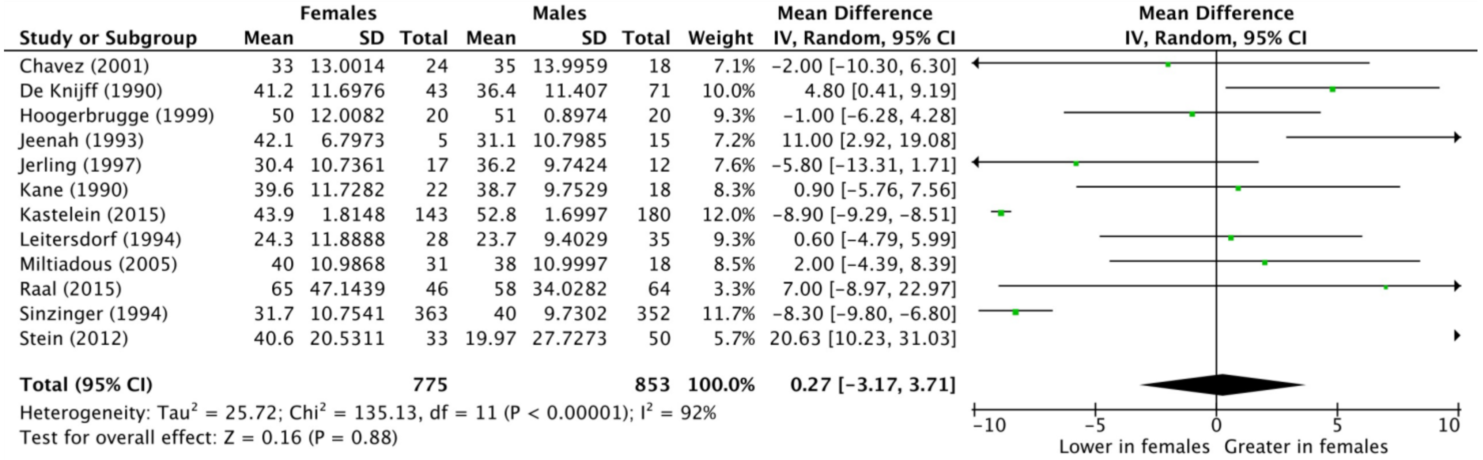

Supplementary Figure 3. Country-specific estimates of sex differences in treatment for familial hypercholesterolemia from observational studies included in the systematic review. When more than one study reported on the treatment of male and female FH patients in the same country, a pooled odds ratio was derived through meta-analysis using a random-effects model. Countries in which data were not available are shown in grey.

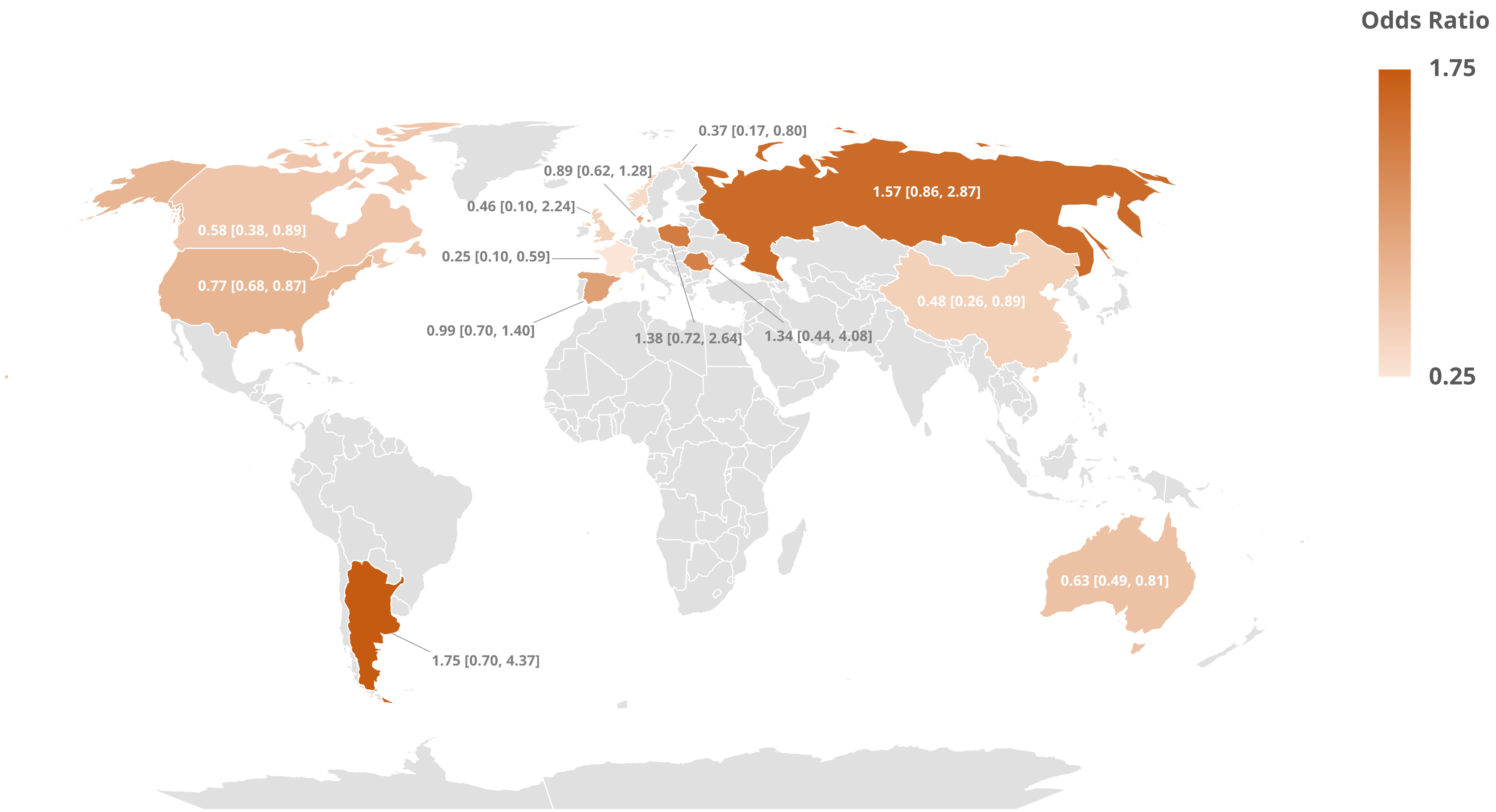

Supplementary Figure 4. Subgroup analysis of sex-differences in treatment with lipid lowering therapies by World Health Organization (WHO) geographical location from observational studies included in the systematic review. Odds ratios (squares) and 95% confidence intervals (CIs) (horizontal lines) for sex differences in treatment with lipid lowering therapies stratified by study sample size were derived using a random-effects model. Diamonds indicate pooled odds ratios and 95% CIs. Solid vertical line indicates null effect. P-values for the test of differences between the subgroup are considered significant at <0.05.

Study or Subgroup	log[Odds Ratio]	SE	Males Total	Females Total	Weight	Odds Ratio IV, Random, 95% CI
**2.3.1 Region of the Americas**						
Agarwala (2023)	−0.0726	0.1597	502	280	5.4%	0.93 [0.68, 1.27]
Amrock (2017)	−0.3857	0.0812	1921	1246	7.1%	0.68 [0.58, 0.80]
Jackson (2020)	−0.2357	0.0198	28431	25367	7.9%	0.79 [0.76, 0.82]
Ryzhaya (2021)	−0.5621	0.2345	304	275	3.9%	0.57 [0.36, 0.90]
Zhao (2019)	−0.4155	0.5378	102	80	1.3%	0.66 [0.23, 1.89]
**Subtotal (95% CI)**			**31260**	**27248**	**25.7%**	**0.76 [0.67, 0.85]**
Heterogeneity: $Tau^2 = 0.01$; $Chi^2 = 6.31$, df = 4 (P = 0.18); $I^2 = 37\%$						
Test for overall effect: Z = 4.79 (P < 0.00001)						
**2.3.2 European Region**						
Arnesen (2020)	−1.1712	0.5286	127	147	1.3%	0.31 [0.11, 0.87]
Beliard (2014)	−0.7985	0.143	843	826	5.8%	0.45 [0.34, 0.60]
Benn (2012)	−0.1165	0.1844	298	204	4.9%	0.89 [0.62, 1.28]
Gallo (2017)	−2.0402	0.6014	56	56	1.0%	0.13 [0.04, 0.42]
Jiménez (2023)	−0.5798	0.1846	1778	1583	4.9%	0.56 [0.39, 0.80]
Korneva (2019)	0.4511	0.3071	116	75	2.9%	1.57 [0.86, 2.87]
Krogh (2016)	−0.6733	0.8313	31	43	0.6%	0.51 [0.10, 2.60]
Matta (2021)	0.5596	0.4675	85	30	1.6%	1.75 [0.70, 4.37]
Mattina (2019)	−1.772	0.5314	84	70	1.3%	0.17 [0.06, 0.48]
Mundal (2014)	−0.844	0.798	30	38	0.6%	0.43 [0.09, 2.05]
Neil (2004)	−1.6094	0.3537	199	211	2.4%	0.20 [0.10, 0.40]
Pérez-Calahorra (2017)	0.207	0.1056	881	851	6.6%	1.23 [1.00, 1.51]
Schreuder (2023)	−0.734	0.1468	1713	1465	5.7%	0.48 [0.36, 0.64]
Vallejo-Vaz (2018)	0	0.1139	626	714	6.4%	1.00 [0.80, 1.25]
Vlad (2021)	0.2927	0.5682	39	22	1.1%	1.34 [0.44, 4.08]
Waluś-Miarka (2017)	0.3221	0.3319	91	63	2.6%	1.38 [0.72, 2.64]
Zamora (2023)	0.2151	0.0474	7952	6747	7.7%	1.24 [1.13, 1.36]
**Subtotal (95% CI)**			**14949**	**13145**	**57.4%**	**0.69 [0.52, 0.91]**
Heterogeneity: $Tau^2 = 0.24$; $Chi^2 = 145.69$, df = 16 (P < 0.00001); $I^2 = 89\%$						
Test for overall effect: Z = 2.60 (P = 0.009)						
**2.3.3 Western Pacific Region**						
Li (2017)	−0.734	0.3128	119	162	2.8%	0.48 [0.26, 0.89]
Pang (2021)	−0.462	0.1282	757	771	6.1%	0.63 [0.49, 0.81]
**Subtotal (95% CI)**			**876**	**933**	**9.0%**	**0.61 [0.48, 0.76]**
Heterogeneity: $Tau^2 = 0.00$; $Chi^2 = 0.65$, df = 1 (P = 0.42); $I^2 = 0\%$						
Test for overall effect: Z = 4.22 (P < 0.0001)						
**2.3.4 International**						
Vallejo-Vaz (2021)	−0.0408	0.0217	21999	19031	7.9%	0.96 [0.92, 1.00]
**Subtotal (95% CI)**			**21999**	**19031**	**7.9%**	**0.96 [0.92, 1.00]**
Heterogeneity: Not applicable						
Test for overall effect: Z = 1.88 (P = 0.06)						
**Total (95% CI)**			**69084**	**60357**	**100.0%**	**0.74 [0.66, 0.85]**
Heterogeneity: $Tau^2 = 0.05$; $Chi^2 = 228.82$, df = 24 (P < 0.00001); $I^2 = 90\%$						
Test for overall effect: Z = 4.50 (P < 0.00001)						
Test for subgroup differences: $Chi^2 = 31.61$, df = 3 (P < 0.00001), $I^2 = 90.5\%$						

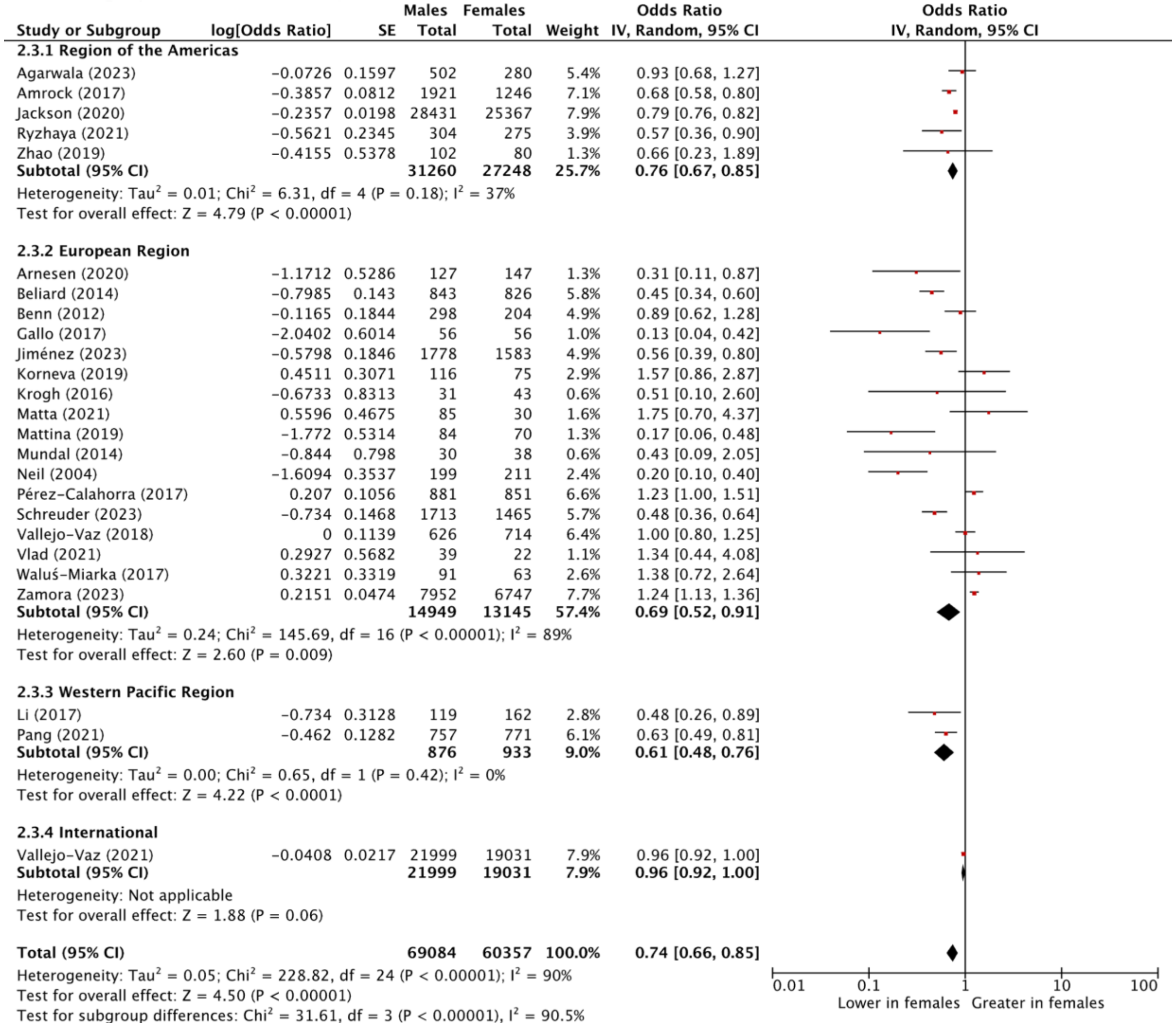

Supplemental Figure 5. Subgroup analysis of sex-differences in treatment with lipid lowering therapies by diagnostic criteria. Genetic criteria subgroup represents studies where the majority (>50%) of participants were diagnosed by genetic testing. Clinical criteria subgroup denotes studies where all participants were diagnosed using any of common clinical criteria (Dutch Lipid Clinic Network; Simon Broome; MEDPED, or Canadian definition). Combination subgroup represents studies where a minority of participants (<50%) were diagnosed using genetic testing, with the remainder diagnosed using common clinical criteria. Odds ratios (squares) and 95% confidence intervals (CIs) (horizontal lines) were derived using a random-effects model. Area of the square is proportional to the inverse variance of the estimate. Diamonds indicate pooled odds ratios and 95% CIs. Solid vertical line indicates null effect. P-values for the test of differences between the subgroup are considered significant at <0.05.

Study or Subgroup	log[Odds Ratio]	SE	Males Total	Females Total	Weight	Odds Ratio IV, Random, 95% CI
**2.8.1 Genetic**						
Arnesen (2020)	−1.1712	0.5286	127	147	1.2%	0.31 [0.11, 0.87]
Gallo (2017)	−2.0413	0.6608	56	56	0.8%	0.13 [0.04, 0.47]
Jiménez (2023)	−0.5798	0.1846	1778	1583	4.7%	0.56 [0.39, 0.80]
Krogh (2016)	−0.6733	0.8313	31	43	0.5%	0.51 [0.10, 2.60]
Li (2017)	−0.734	0.3128	119	162	2.7%	0.48 [0.26, 0.89]
Mattina (2019)	−1.772	0.5314	84	70	1.2%	0.17 [0.06, 0.48]
Mundal (2014)	−0.8473	0.7758	30	38	0.6%	0.43 [0.09, 1.96]
Perez de la Isla (2016)	−0.2614	0.1188	1183	987	6.1%	0.77 [0.61, 0.97]
**Subtotal (95% CI)**			**3408**	**3086**	**18.0%**	**0.44 [0.30, 0.66]**
Heterogeneity: Tau² = 0.15; Chi² = 17.80, df = 7 (P = 0.01); I² = 61%						
Test for overall effect: Z = 4.05 (P < 0.0001)						
**2.8.2 Clinical**						
Amrock (2017)	−0.3857	0.0812	1921	1246	6.9%	0.68 [0.58, 0.80]
Matta (2021)	0.5596	0.4675	85	30	1.5%	1.75 [0.70, 4.37]
Neil (2004)	−1.6094	0.3537	199	211	2.3%	0.20 [0.10, 0.40]
Pérez-Calahorra (2017)	0.207	0.1056	881	851	6.4%	1.23 [1.00, 1.51]
Ryzhaya (2021)	−0.5621	0.2345	304	275	3.8%	0.57 [0.36, 0.90]
Vlad (2021)	0.2927	0.5682	39	22	1.1%	1.34 [0.44, 4.08]
Zhao (2019)	−0.4155	0.5378	102	80	1.2%	0.66 [0.23, 1.89]
**Subtotal (95% CI)**			**3531**	**2715**	**23.2%**	**0.74 [0.48, 1.12]**
Heterogeneity: Tau² = 0.22; Chi² = 41.80, df = 6 (P < 0.00001); I² = 86%						
Test for overall effect: Z = 1.42 (P = 0.16)						
**2.8.3 Combination**						
Agarwala (2023)	−0.0726	0.1597	502	280	5.2%	0.93 [0.68, 1.27]
Beliard (2014)	−0.7985	0.143	843	826	5.6%	0.45 [0.34, 0.60]
Benn (2012)	−0.1165	0.1844	298	204	4.7%	0.89 [0.62, 1.28]
Pang (2021)	−0.462	0.1282	757	771	5.9%	0.63 [0.49, 0.81]
Schreuder (2023)	−0.734	0.1468	1713	1465	5.5%	0.48 [0.36, 0.64]
Vallejo-Vaz (2018)	0	0.1139	626	714	6.3%	1.00 [0.80, 1.25]
Vallejo-Vaz (2021)	−0.0408	0.0217	21999	19031	7.8%	0.96 [0.92, 1.00]
Waluś-Miarka (2017)	0.3221	0.3319	91	63	2.5%	1.38 [0.72, 2.64]
**Subtotal (95% CI)**			**26829**	**23354**	**43.5%**	**0.77 [0.61, 0.97]**
Heterogeneity: Tau² = 0.09; Chi² = 59.23, df = 7 (P < 0.00001); I² = 88%						
Test for overall effect: Z = 2.21 (P = 0.03)						
**2.8.4 LDL-C cut-offs**						
Jackson (2020)	−0.2357	0.0198	28431	25367	7.8%	0.79 [0.76, 0.82]
Zamora (2023)	0.2151	0.0474	7952	6747	7.5%	1.24 [1.13, 1.36]
**Subtotal (95% CI)**			**36383**	**32114**	**15.3%**	**0.99 [0.64, 1.54]**
Heterogeneity: Tau² = 0.10; Chi² = 77.01, df = 1 (P < 0.00001); I² = 99%						
Test for overall effect: Z = 0.05 (P = 0.96)						
**Total (95% CI)**			**70151**	**61269**	**100.0%**	**0.73 [0.65, 0.83]**
Heterogeneity: Tau² = 0.05; Chi² = 224.53, df = 24 (P < 0.00001); I² = 89%						
Test for overall effect: Z = 4.87 (P < 0.00001)						
Test for subgroup differences: Chi² = 8.08, df = 3 (P = 0.04), I² = 62.9%						

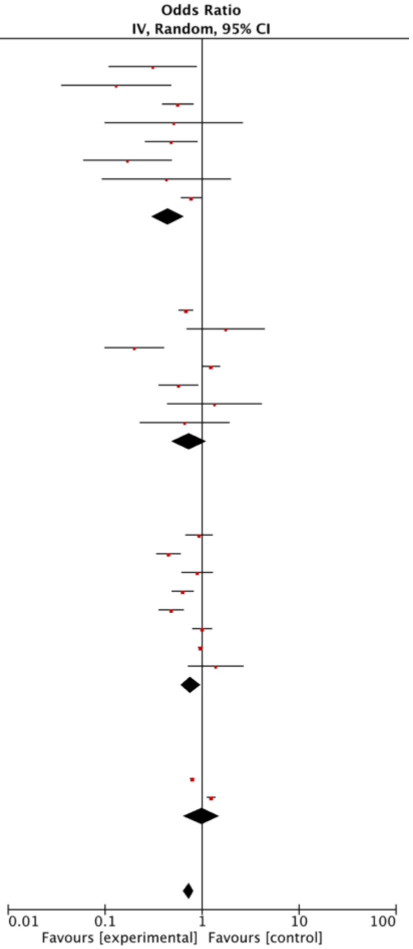

Supplementary Figure 6. Subgroup analysis of sex-differences in treatment with lipid lowering therapies by year of publication of observational studies included in the systematic review. Figure depicts odds ratios (squares) and 95% confidence intervals (CIs) (horizontal lines) for sex differences in treatment with lipid lowering therapies stratified by year of publication. Diamonds indicate pooled odds ratios and 95% CIs. Solid vertical line indicates null effect. Odds ratios were derived using a random-effects model. P-values for the test of differences between the subgroup are considered significant at <0.05.

Study or Subgroup	log[Odds Ratio]	SE	Males Total	Females Total	Weight	Odds Ratio IV, Random, 95% CI
**2.1.1 Before 2016**						
Beliard (2014)	−0.7985	0.143	843	826	5.8%	0.45 [0.34, 0.60]
Benn (2012)	−0.1165	0.1844	298	204	4.9%	0.89 [0.62, 1.28]
Krogh (2016)	−0.6733	0.8313	31	43	0.6%	0.51 [0.10, 2.60]
Mundal (2014)	−0.844	0.798	30	38	0.6%	0.43 [0.09, 2.05]
Neil (2004)	−1.6094	0.3537	199	211	2.4%	0.20 [0.10, 0.40]
**Subtotal (95% CI)**			**1401**	**1322**	**14.3%**	**0.46 [0.26, 0.81]**
Heterogeneity: $Tau^2 = 0.25$; $Chi^2 = 16.86$, df = 4 (P = 0.002); $I^2 = 76\%$						
Test for overall effect: Z = 2.69 (P = 0.007)						
**2.1.2 After 2016**						
Agarwala (2023)	−0.0726	0.1597	502	280	5.4%	0.93 [0.68, 1.27]
Amrock (2017)	−0.3857	0.0812	1921	1246	7.1%	0.68 [0.58, 0.80]
Arnesen (2020)	−1.1712	0.5286	127	147	1.3%	0.31 [0.11, 0.87]
Gallo (2017)	−2.0402	0.6014	56	56	1.0%	0.13 [0.04, 0.42]
Jackson (2020)	−0.2357	0.0198	28431	25367	7.9%	0.79 [0.76, 0.82]
Jiménez (2023)	−0.5798	0.1846	1778	1583	4.9%	0.56 [0.39, 0.80]
Korneva (2019)	0.4511	0.3071	116	75	2.9%	1.57 [0.86, 2.87]
Li (2017)	−0.734	0.3128	119	162	2.8%	0.48 [0.26, 0.89]
Matta (2021)	0.5596	0.4675	85	30	1.6%	1.75 [0.70, 4.37]
Mattina (2019)	−1.772	0.5314	84	70	1.3%	0.17 [0.06, 0.48]
Pang (2021)	−0.462	0.1282	757	771	6.1%	0.63 [0.49, 0.81]
Pérez-Calahorra (2017)	0.207	0.1056	881	851	6.6%	1.23 [1.00, 1.51]
Ryzhaya (2021)	−0.5621	0.2345	304	275	3.9%	0.57 [0.36, 0.90]
Schreuder (2023)	−0.734	0.1468	1713	1465	5.7%	0.48 [0.36, 0.64]
Vallejo-Vaz (2018)	0	0.1139	626	714	6.4%	1.00 [0.80, 1.25]
Vallejo-Vaz (2021)	−0.0408	0.0217	21999	19031	7.9%	0.96 [0.92, 1.00]
Vlad (2021)	0.2927	0.5682	39	22	1.1%	1.34 [0.44, 4.08]
Waluś-Miarka (2017)	0.3221	0.3319	91	63	2.6%	1.38 [0.72, 2.64]
Zamora (2023)	0.2151	0.0474	7952	6747	7.7%	1.24 [1.13, 1.36]
Zhao (2019)	−0.4155	0.5378	102	80	1.3%	0.66 [0.23, 1.89]
**Subtotal (95% CI)**			**67683**	**59035**	**85.7%**	**0.80 [0.70, 0.91]**
Heterogeneity: $Tau^2 = 0.05$; $Chi^2 = 188.59$, df = 19 (P < 0.00001); $I^2 = 90\%$						
Test for overall effect: Z = 3.31 (P = 0.0009)						
**Total (95% CI)**			**69084**	**60357**	**100.0%**	**0.74 [0.66, 0.85]**
Heterogeneity: $Tau^2 = 0.05$; $Chi^2 = 228.82$, df = 24 (P < 0.00001); $I^2 = 90\%$						
Test for overall effect: Z = 4.50 (P < 0.00001)						
Test for subgroup differences: $Chi^2 = 3.49$, df = 1 (P = 0.06), $I^2 = 71.4\%$						

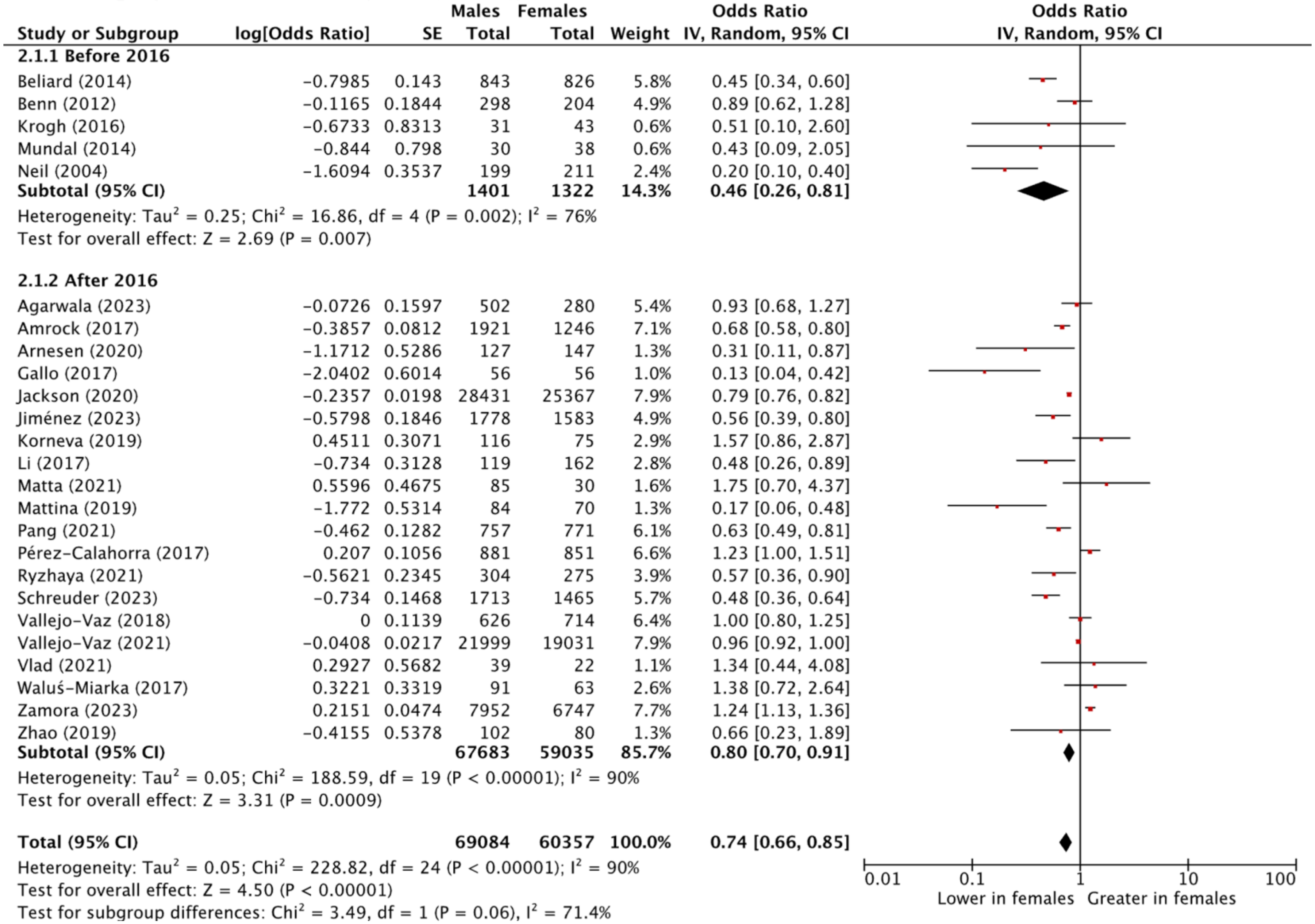

Supplementary Figure 7. Subgroup analysis of sex-differences in treatment with lipid lowering therapies by study sample size, from observational studies included in the systematic review. Figure depicts odds ratios (squares) and 95% confidence intervals (CIs) (horizontal lines) for sex differences in treatment with lipid lowering therapies stratified by study sample size. Diamonds indicate pooled odds ratios and 95% CIs. Solid vertical line indicates null effect. Odds ratios were derived using a random-effects model. P-values for the test of differences between the subgroup are considered significant at <0.05.

Study or Subgroup	log[Odds Ratio]	SE	Weight	Odds Ratio IV, Random, 95% CI
**2.2.1 Fewer than 1000**				
Agarwala (2023)	−0.0726	0.1597	5.4%	0.93 [0.68, 1.27]
Arnesen (2020)	−1.1712	0.5286	1.3%	0.31 [0.11, 0.87]
Benn (2012)	−0.1165	0.1844	4.9%	0.89 [0.62, 1.28]
Gallo (2017)	−2.0402	0.6014	1.0%	0.13 [0.04, 0.42]
Korneva (2019)	0.4511	0.3071	2.9%	1.57 [0.86, 2.87]
Krogh (2016)	−0.6733	0.8313	0.6%	0.51 [0.10, 2.60]
Li (2017)	−0.734	0.3128	2.8%	0.48 [0.26, 0.89]
Matta (2021)	0.5596	0.4675	1.6%	1.75 [0.70, 4.37]
Mattina (2019)	−1.772	0.5314	1.3%	0.17 [0.06, 0.48]
Mundal (2014)	−0.844	0.798	0.6%	0.43 [0.09, 2.05]
Neil (2004)	−1.6094	0.3537	2.4%	0.20 [0.10, 0.40]
Ryzhaya (2021)	−0.5621	0.2345	3.9%	0.57 [0.36, 0.90]
Vlad (2021)	0.2927	0.5682	1.1%	1.34 [0.44, 4.08]
Waluś-Miarka (2017)	0.3221	0.3319	2.6%	1.38 [0.72, 2.64]
Zhao (2019)	−0.4155	0.5378	1.3%	0.66 [0.23, 1.89]
**Subtotal (95% CI)**			**33.8%**	**0.62 [0.43, 0.89]**
Heterogeneity: $Tau^2 = 0.31$; $Chi^2 = 53.33$, df = 14 ($P < 0.00001$); $I^2 = 74\%$				
Test for overall effect: $Z = 2.63$ ($P = 0.009$)				
**2.2.2 More than 1000**				
Amrock (2017)	−0.3857	0.0812	7.1%	0.68 [0.58, 0.80]
Beliard (2014)	−0.7985	0.143	5.8%	0.45 [0.34, 0.60]
Jackson (2020)	−0.2357	0.0198	7.9%	0.79 [0.76, 0.82]
Jiménez (2023)	−0.5798	0.1846	4.9%	0.56 [0.39, 0.80]
Pang (2021)	−0.462	0.1282	6.1%	0.63 [0.49, 0.81]
Pérez-Calahorra (2017)	0.207	0.1056	6.6%	1.23 [1.00, 1.51]
Schreuder (2023)	−0.734	0.1468	5.7%	0.48 [0.36, 0.64]
Vallejo-Vaz (2018)	0	0.1139	6.4%	1.00 [0.80, 1.25]
Vallejo-Vaz (2021)	−0.0408	0.0217	7.9%	0.96 [0.92, 1.00]
Zamora (2023)	0.2151	0.0474	7.7%	1.24 [1.13, 1.36]
**Subtotal (95% CI)**			**66.2%**	**0.78 [0.68, 0.91]**
Heterogeneity: $Tau^2 = 0.04$; $Chi^2 = 171.17$, df = 9 ($P < 0.00001$); $I^2 = 95\%$				
Test for overall effect: $Z = 3.25$ ($P = 0.001$)				
**Total (95% CI)**			**100.0%**	**0.74 [0.66, 0.85]**
Heterogeneity: $Tau^2 = 0.05$; $Chi^2 = 228.82$, df = 24 ($P < 0.00001$); $I^2 = 90\%$				
Test for overall effect: $Z = 4.50$ ($P < 0.00001$)				
Test for subgroup differences: $Chi^2 = 1.43$, df = 1 ($P = 0.23$), $I^2 = 30.1\%$				

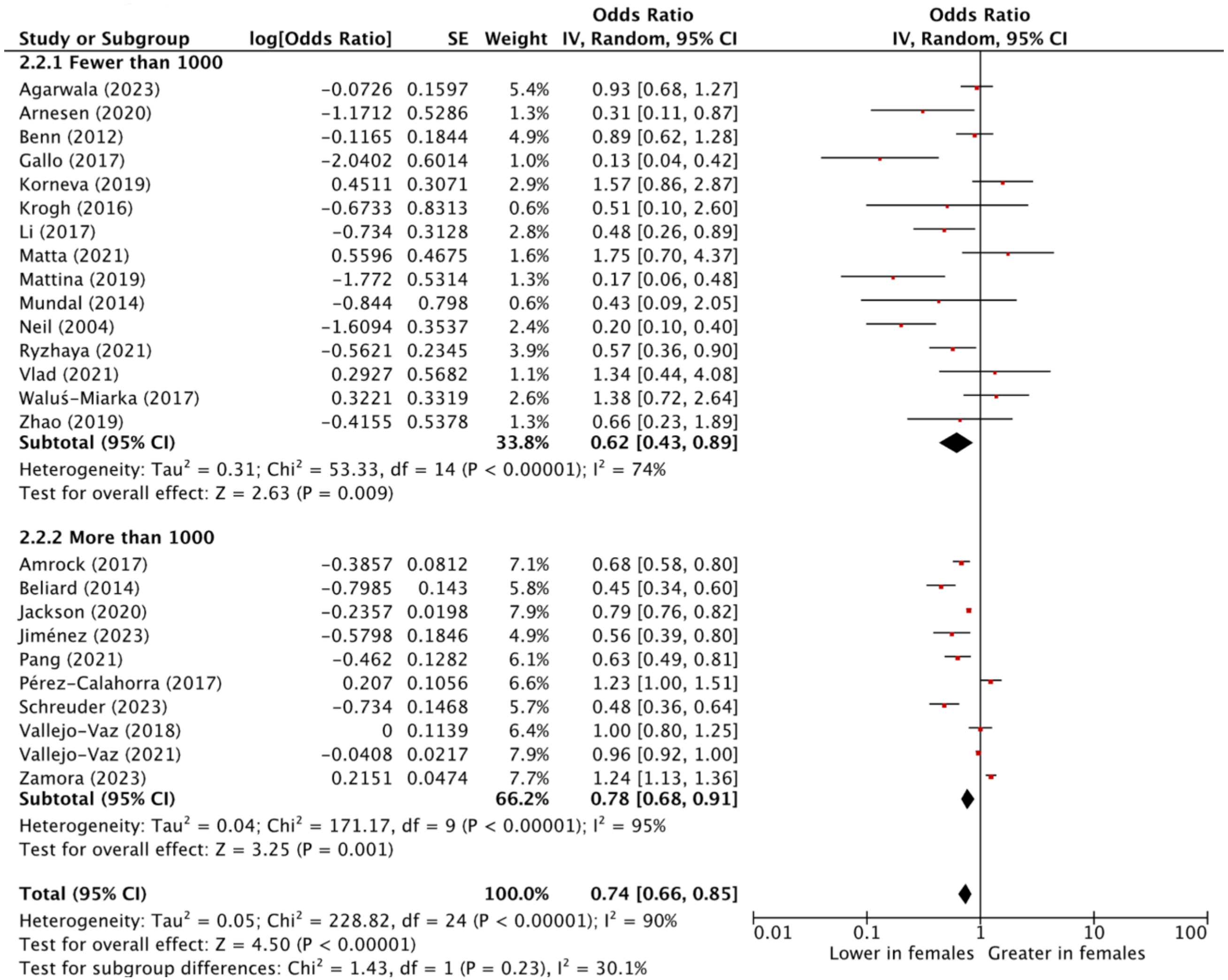

Supplementary Figure 8. Meta-analysis of sex differences in treatment with statins from observational studies included in the systematic review. Figure depicts study-level odds ratios (squares) and 95% confidence intervals (CIs) (horizontal lines). Area of the square is proportional to the inverse variance of the estimate. Diamond indicates the pooled odds ratio and 95% CI derived under the random-effects model. Solid vertical line indicates null effect.

	Females		Males			Odds Ratio
**Study or Subgroup**	**Events**	**Total**	**Events**	**Total**	**Weight**	**IV, Random, 95% CI**
Agarwala (2023)	344	502	196	280	7.4%	0.93 [0.68, 1.28]
Amrock (2017)	1281	1826	930	1158	9.8%	0.58 [0.48, 0.69]
Gallo (2017)	38	56	52	56	1.3%	0.16 [0.05, 0.52]
Jackson (2020)	16954	28431	16695	25367	11.5%	0.77 [0.74, 0.79]
Johansen (2023)	183	207	198	231	4.1%	1.27 [0.72, 2.23]
Korneva (2019)	80	116	44	75	3.8%	1.57 [0.86, 2.87]
Krogh (2016)	27	31	40	43	0.8%	0.51 [0.10, 2.44]
Matta (2021)	33	85	8	30	2.0%	1.75 [0.70, 4.38]
Mattina (2019)	56	84	64	70	1.9%	0.19 [0.07, 0.49]
Mundal (2014)	25	30	35	38	0.8%	0.43 [0.09, 1.96]
Neil (2004)	159	199	211	211	0.3%	0.01 [0.00, 0.15]
Pang (2021)	526	757	586	771	9.0%	0.72 [0.57, 0.90]
Pérez-Calahorra (2017)	281	881	234	851	9.3%	1.23 [1.00, 1.52]
Ryzhaya (2021)	220	249	207	231	4.0%	0.88 [0.50, 1.56]
Schreuder (2023)	1562	1713	1400	1465	7.7%	0.48 [0.36, 0.65]
Vallejo-Vaz (2021)	17797	21999	15453	19031	11.4%	0.98 [0.93, 1.03]
Vlad (2021)	15	39	7	22	1.5%	1.34 [0.44, 4.04]
Waluś-Miarka (2017)	59	91	36	63	3.4%	1.38 [0.72, 2.67]
Zamora (2023)	308	7952	380	6747	10.2%	0.68 [0.58, 0.79]
**Total (95% CI)**		**65248**		**56740**	**100.0%**	**0.79 [0.69, 0.92]**
Total events	39948		36776			

Heterogeneity: $Tau^2 = 0.05$; $Chi^2 = 154.78$, df = 18 ($P < 0.00001$); $I^2 = 88\%$

Test for overall effect: $Z = 3.15$ ($P = 0.002$)

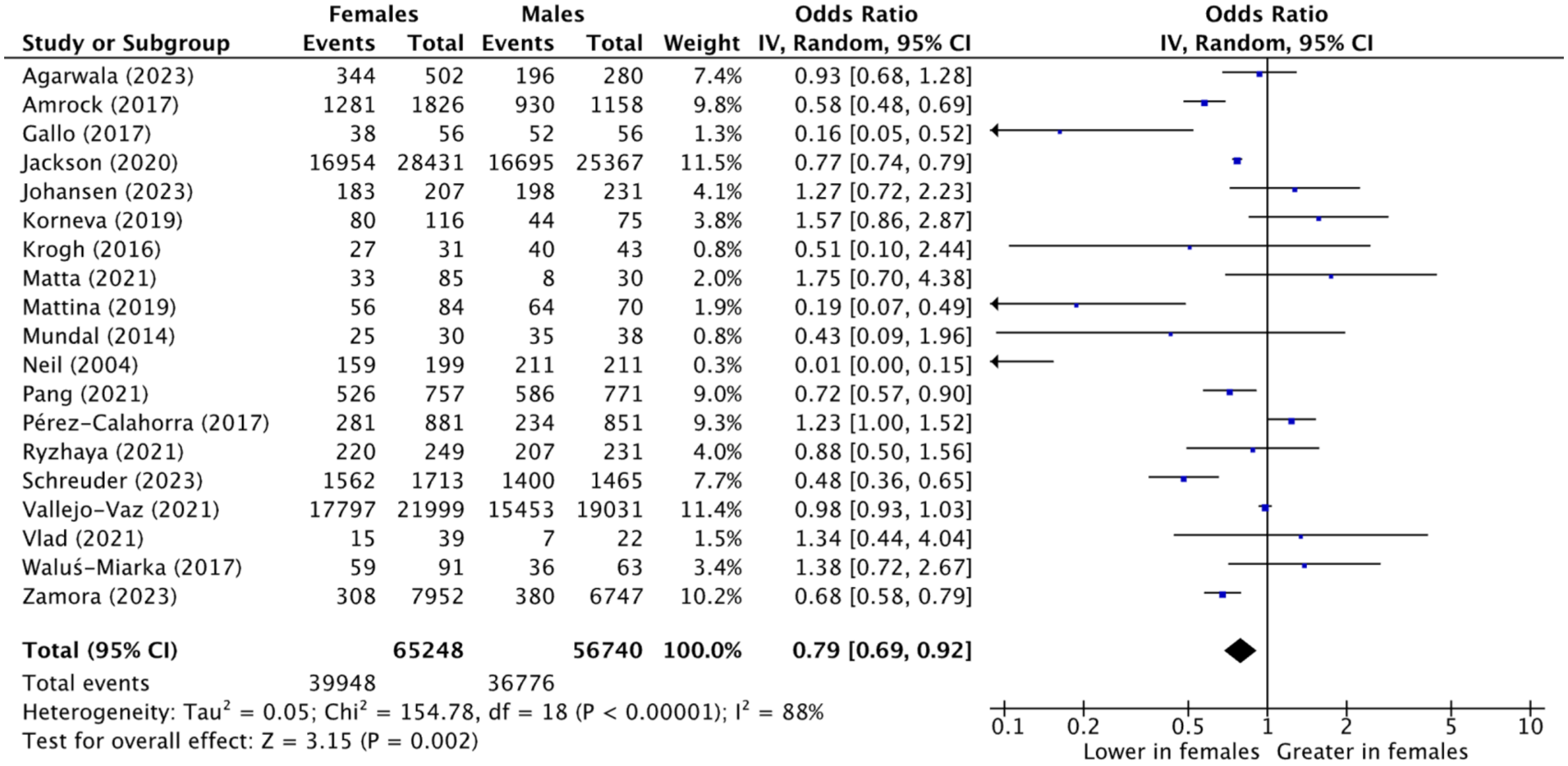

Supplementary Figure 9. Meta-analysis of sex difference in treatment with high-intensity statin doses from observational studies included in the systematic review. Figure depicts study-level odds ratios (squares) and 95% confidence intervals (CIs) (horizontal lines). Area of the square is proportional to the inverse variance of the estimate. Diamond indicates the pooled odds ratio and 95% CI derived under the random-effects model. Solid vertical line indicates null effect.

Study or Subgroup	log[Odds Ratio]	SE	Females Total	Males Total	Weight	Odds Ratio IV, Random, 95% CI
Agarwala (2023)	−0.4528	0.1512	502	280	9.7%	0.64 [0.47, 0.86]
Amrock (2017)	−0.3711	0.1746	1259	918	8.5%	0.69 [0.49, 0.97]
Arnesen (2020)	−1.3093	0.2999	127	147	4.4%	0.27 [0.15, 0.49]
Jiménez (2023)	−0.478	0.175	1778	1583	8.5%	0.62 [0.44, 0.87]
Krogh (2016)	−0.9676	0.5473	25	36	1.6%	0.38 [0.13, 1.11]
Perez de la Isla (2016)	−0.2614	0.1188	1183	987	11.6%	0.77 [0.61, 0.97]
Ryzhaya (2021)	−0.4005	0.1809	249	231	8.2%	0.67 [0.47, 0.96]
Schreuder (2023)	−0.1815	0.0739	1713	1465	14.3%	0.83 [0.72, 0.96]
Vallejo-Vaz (2021)	−0.2744	0.0276	21999	19031	16.3%	0.76 [0.72, 0.80]
Zamora (2017)	−0.5621	0.0371	7952	6747	16.0%	0.57 [0.53, 0.61]
Zhao (2019)	−0.5447	0.763	102	80	0.9%	0.58 [0.13, 2.59]
**Total (95% CI)**			**36889**	**31505**	**100.0%**	**0.66 [0.57, 0.76]**

Heterogeneity: $Tau^2 = 0.03$; $Chi^2 = 57.89$, $df = 10$ ($P < 0.00001$); $I^2 = 83\%$

Test for overall effect: $Z = 5.74$ ($P < 0.00001$)

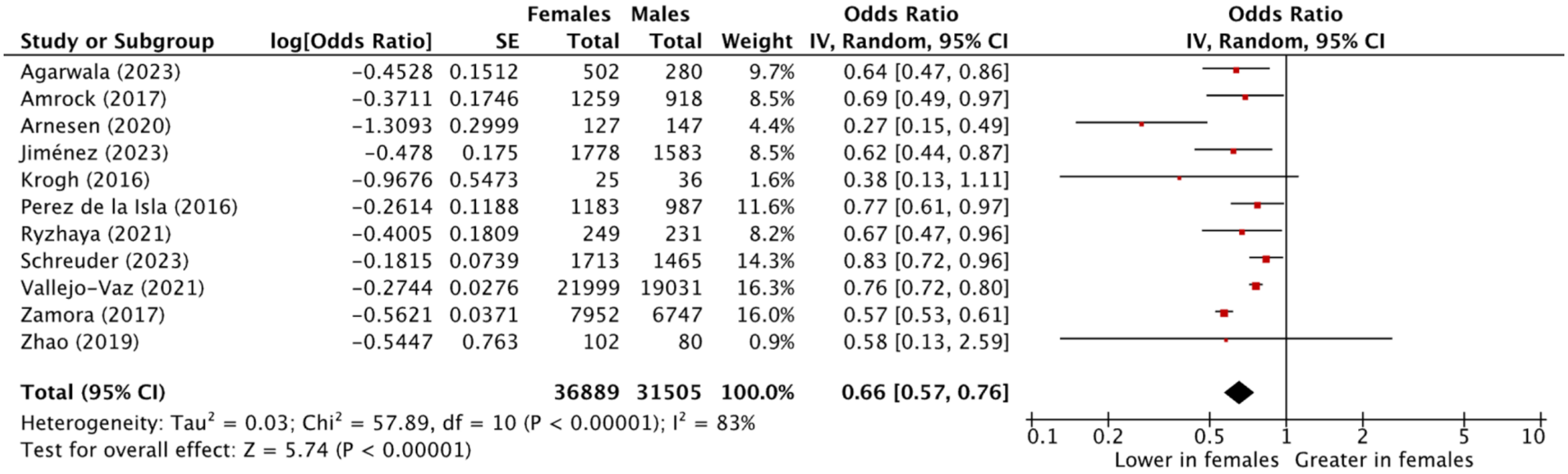

Supplementary Figure 10. Meta-analysis of sex differences in treatment with ezetimibe from observational studies included in the systematic review. Figure depicts study-level odds ratios (squares) and 95% confidence intervals (CIs) (horizontal lines). Area of the square is proportional to the inverse variance of the estimate. Diamond indicates the pooled odds ratio and 95% CI derived under the random-effects model. Solid vertical line indicates null effect.

	Females		Males			Odds Ratio
**Study or Subgroup**	**Events**	**Total**	**Events**	**Total**	**Weight**	**IV, Random, 95% CI**
Agarwala (2023)	163	502	112	280	10.1%	0.72 [0.53, 0.98]
Amrock (2017)	560	1921	466	1246	14.4%	0.69 [0.59, 0.80]
Gallo (2017)	38	56	52	56	1.6%	0.16 [0.05, 0.52]
Krogh (2016)	5	26	8	36	1.4%	0.83 [0.24, 2.92]
Mattina (2019)	20	84	32	70	3.9%	0.37 [0.19, 0.74]
Pang (2021)	315	757	381	771	13.0%	0.73 [0.60, 0.89]
Ryzhaya (2021)	102	249	119	231	8.7%	0.65 [0.46, 0.94]
Schreuder (2023)	908	1713	942	1465	14.6%	0.63 [0.54, 0.72]
Vallejo-Vaz (2021)	5148	21999	4929	19039	16.5%	0.87 [0.84, 0.91]
Vlad (2021)	7	39	11	22	1.6%	0.22 [0.07, 0.70]
Zamora (2023)	308	7952	380	6747	14.3%	0.68 [0.58, 0.79]
**Total (95% CI)**		**35298**		**29963**	**100.0%**	**0.67 [0.57, 0.78]**
Total events	7574		7432			

Heterogeneity: $Tau^2 = 0.04$; $Chi^2 = 53.47$, df = 10 (P < 0.00001); $I^2 = 81\%$

Test for overall effect: Z = 5.15 (P < 0.00001)

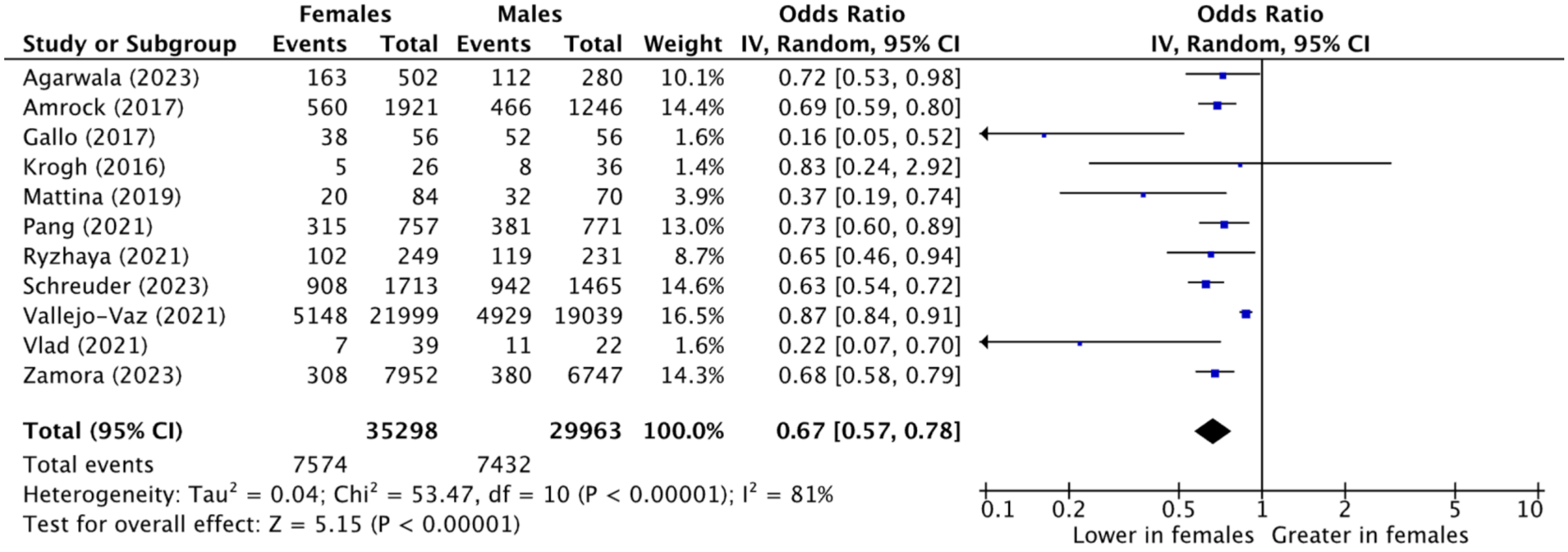

Supplementary Figure 11. Meta-analysis of sex differences in treatment with PCSK9 inhibitors from observational studies included in the systematic review. Figure depicts study-level odds ratios (squares) and 95% confidence intervals (CIs) (horizontal lines). Area of the square is proportional to the inverse variance of the estimate. Diamond indicates pooled odds ratio and 95% CI derived using a random-effects model. Solid vertical line indicates null effect.

Study or Subgroup	log[Odds Ratio]	SE	Females Total	Males Total	Weight	Odds Ratio IV, Random, 95% CI
Agarwala (2023)	−0.6539	0.232	502	280	10.6%	0.52 [0.33, 0.82]
Amrock (2017)	−0.1985	0.1855	1921	1246	12.0%	0.82 [0.57, 1.18]
Jackson (2020)	0.174	0.059	28431	25367	15.3%	1.19 [1.06, 1.34]
Jiménez (2023)	−0.6733	0.1501	1778	1583	13.1%	0.51 [0.38, 0.68]
Pang (2021)	−0.5978	0.2306	757	771	10.6%	0.55 [0.35, 0.86]
Ryzhaya (2021)	−0.3857	0.2581	247	231	9.8%	0.68 [0.41, 1.13]
Schreuder (2023)	−0.3147	0.1445	2713	1165	13.3%	0.73 [0.55, 0.97]
Vallejo-Vaz (2021)	−0.3425	0.061	21999	19031	15.3%	0.71 [0.63, 0.80]
**Total (95% CI)**			**58348**	**49674**	**100.0%**	**0.70 [0.54, 0.91]**

Heterogeneity: $Tau^2 = 0.11$; $Chi^2 = 62.75$, df = 7 ($P < 0.00001$); $I^2 = 89\%$
Test for overall effect: Z = 2.66 (P = 0.008)

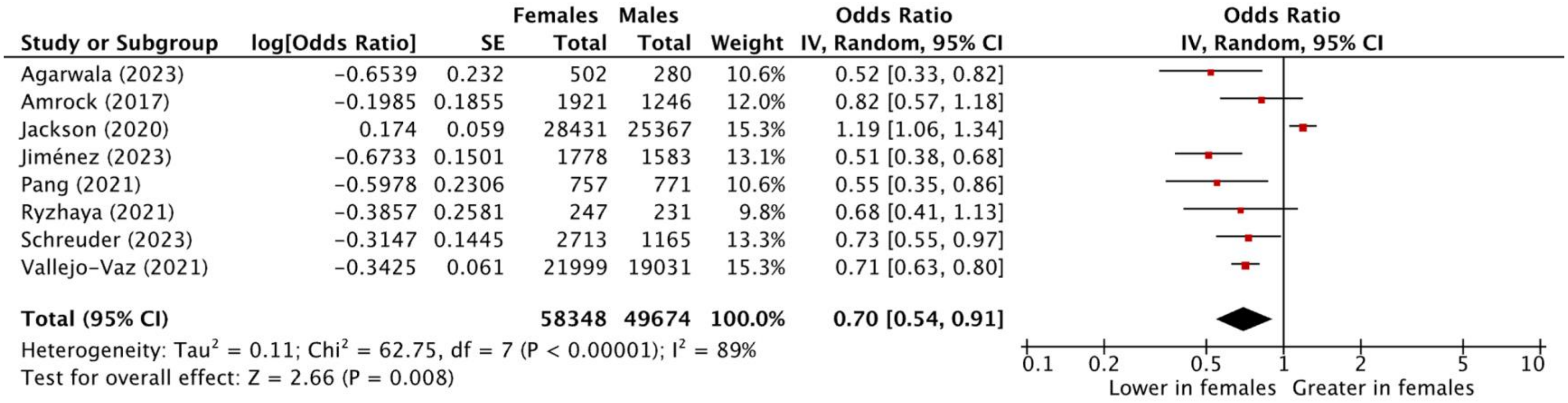

Supplementary Figure 12. Meta-regression plot of effect sizes in meta-analysis of sex differences in treatment with any lipid lowering therapy by year of publication of from observational studies included in the systematic review. Vertical axis depicts odds ratios, while horizontal axis shows year of publication in included studies. Bubbles (filled circles) represent study-level odds ratios with the size representing variance associated with study-level estimates. We also display the fitted regression line. Year of publication explained approximately 22.79% (R^2) of the variation between studies ($p = 0.01$). The meta-regression was conducted using a mixed-effects model with restricted maximum likelihood estimator of between-study variance (tau^2).

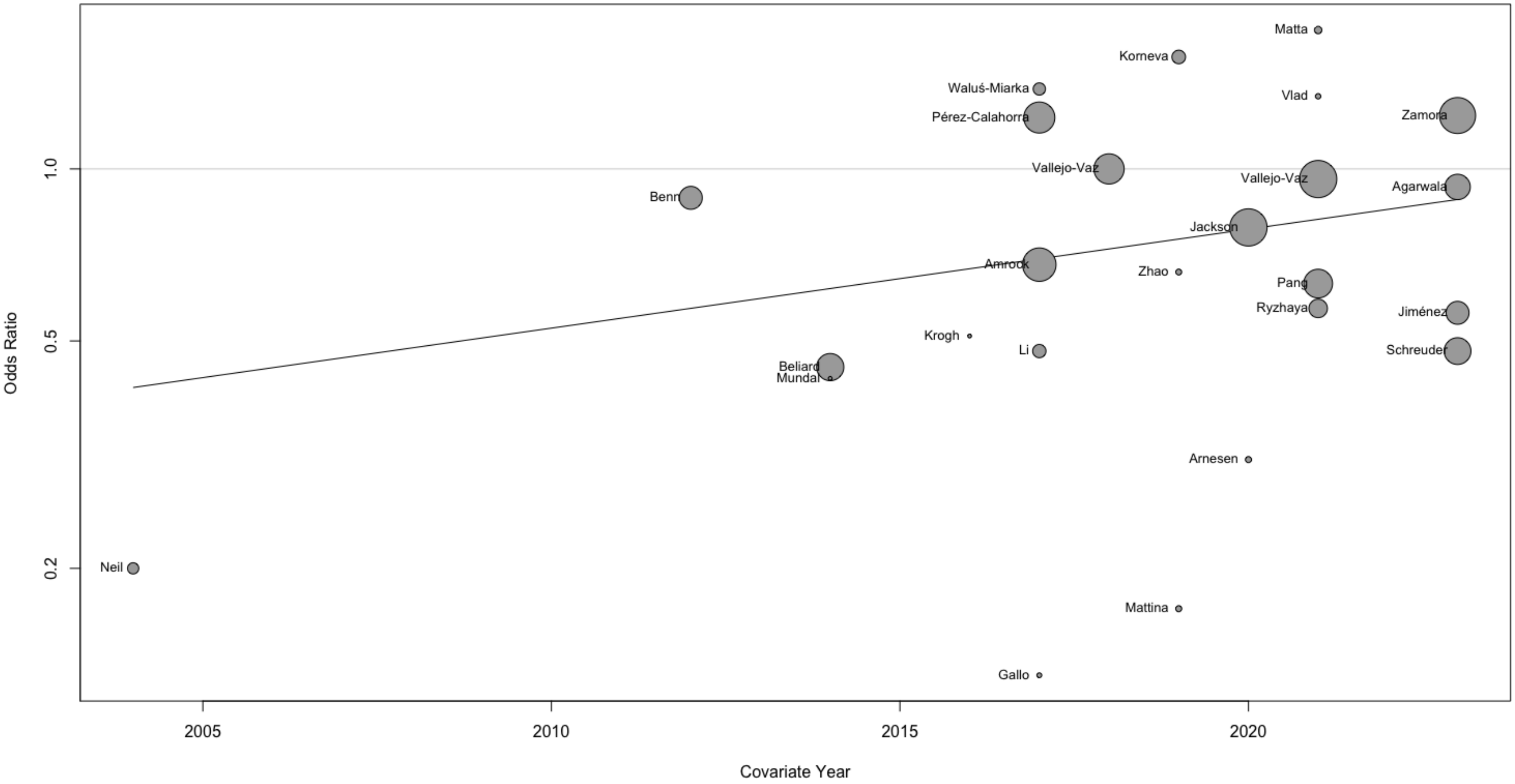

Supplementary Figure 13. Funnel plot of meta-analysis of sex differences in treatment with any lipid lowering therapy with test of asymmetry, from observational studies included in the systematic review. Figure depicts funnel plot for meta-analysis of sex differences in treatment with any lipid lowering therapy. Study-level odds ratios (OR) are plotted on the horizontal axis while the vertical axis shows standard errors. Dotted vertical line depicts the pooled OR derived under the random-effects model. Dashed vertical and horizontal lines indicate OR and expected 95% confidence intervals, assuming no heterogeneity between studies. We display results of egger's test; p-values <0.10 suggest the presence of publication bias.

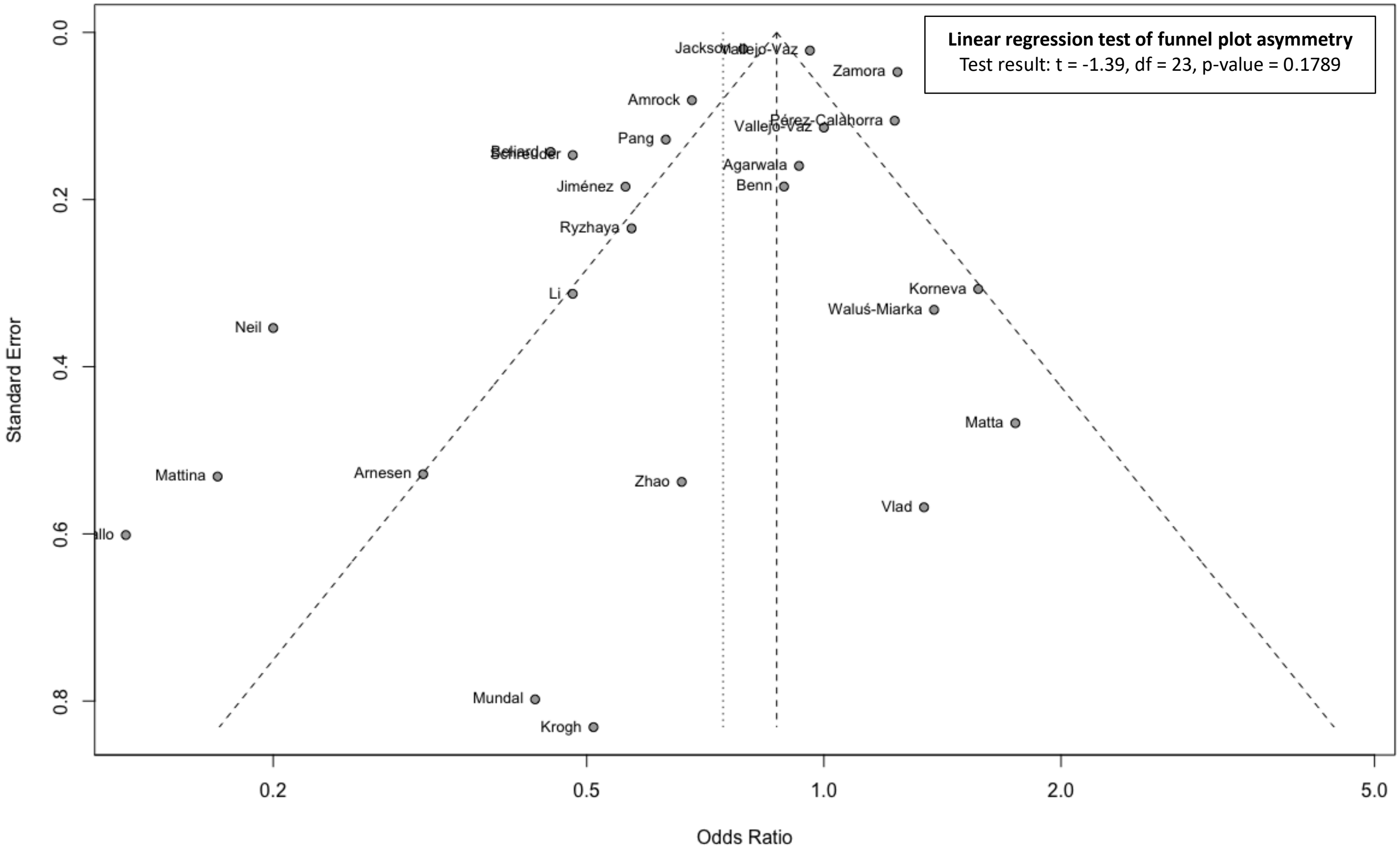

Appendix 3: Quality assessment of studies included in systematic review.

Author	Year	Selection bias	Design	Confounders	Blinding	Data collection methods	Withdrawals and dropouts	Overall
**Observational studies: All qualitative studies on TRT (studies meta-analyzed in grey (n=25))**								
**Agarwala**	2023	Strong	Moderate	Strong	Weak	Strong	Strong	Moderate
**Alonso**	2021	Strong	Moderate	Strong	Strong	Strong	Strong	Strong
**Amrock**	2017	Strong	Moderate	Strong	Moderate	Moderate	Strong	Strong
**Arnesen**	2020	Strong	Moderate	Strong	Moderate	Strong	Strong	Strong
**Beliard**	2014	Strong	Moderate	Moderate	Moderate	Moderate	Strong	Moderate
**Benn**	2012	Strong	Moderate	Strong	Strong	Strong	Strong	Strong
**deGoma**	2016	Strong	Moderate	Strong	Strong	Strong	Strong	Moderate
**Galema-Boers**	2014	Strong	Moderate	Moderate	Moderate	Strong	Strong	Moderate
**Gallo**	2017	Strong	Strong	Strong	Moderate	Strong	Strong	Strong
**Iyen**	2021	Strong	Moderate	Moderate	Strong	Strong	Strong	Strong
**Jackson**	2021	Strong	Moderate	Moderate	Strong	Moderate	Strong	Moderate
**Jiménez**	2023	Strong	Moderate	Moderate	Moderate	Strong	Strong	Moderate
**Kłosiewicz-Latoszek**	2018	Strong	Strong	Moderate	Strong	Strong	Strong	Strong
**Korneva**	2019	Moderate	Moderate	Strong	Strong	Weak	Weak	Weak
**Krogh**	2016	Strong	Moderate	Strong	Strong	Strong	Strong	Strong
**Leduc**	2016	Strong	Weak	Moderate	Moderate	Moderate	Moderate	Weak
**Li**	2017	Moderate	Moderate	Strong	Weak	Strong	Strong	Moderate
**Matta**	2021	Moderate	Weak	Strong	Weak	Moderate	Moderate	Moderate
**Mattina**	2019	Strong	Moderate	Moderate	Moderate	Strong	Moderate	Moderate
**Mundal**	2014	Strong	Moderate	Strong	Strong	Strong	Strong	Strong
**Neil**	2004	Strong	Moderate	Moderate	Strong	Strong	Strong	Strong
**Pang**	2021	Strong	Strong	Moderate	Strong	Strong	Moderate	Strong
**Perez de Isla**	2016	Strong	Moderate	Strong	Weak	Strong	Strong	Moderate
**Pérez-Calahorra**	2017	Strong	Moderate	Moderate	Moderate	Strong	Strong	Strong

**Piljman**	2010	Strong	Strong	Strong	Moderate	Strong	Moderate	Strong
**Razek**	2018	Strong	Strong	Moderate	Strong	Strong	Moderate	Strong
**Roy**	2022	Strong	Strong	Moderate	Strong	Strong	Moderate	Strong
**Ryzhaya**	2021	Strong	Moderate	Moderate	Strong	Strong	Strong	Strong
**Schreuder**	2023	Strong	Strong	Strong	Strong	Strong	Strong	Strong
**Vallejo-Vaz**	2018	Moderate	Moderate	Strong	Strong	Strong	Strong	Strong
**Vallejo-Vaz**	2021	Strong	Moderate	Strong	Strong	Strong	Strong	Strong
**Vlad**	2021	Strong	Moderate	Strong	Weak	Strong	Strong	Moderate
**Waluś-Miarka**	2017	Moderate	Strong	Strong	Moderate	Moderate	Moderate	Moderate
**Zamora**	2017	Weak	Strong	Strong	Strong	Weak	Moderate	Moderate
**Zamora**	2023	Weak	Strong	Strong	Strong	Weak	Moderate	Moderate
**Zhao**	2019	Moderate	Moderate	Strong	Weak	Moderate	Moderate	Moderate
**All qualitative studies on CVD (studies meta-analyzed in grey (n=57))**								
**Agarwala**	2021	Strong	Moderate	Strong	Weak	Strong	Strong	Moderate
**Agarwala**	2023	Strong	Moderate	Strong	Weak	Strong	Strong	Moderate
**Ahmad**	2016	Moderate	Moderate	Weak	Weak	Strong	Strong	Weak
**Allard**	2014	Strong	Moderate	Strong	Weak	Strong	Strong	Moderate
**Alonso**	2008	Strong	Moderate	Strong	Strong	Strong	Strong	Strong
**Alonso**	2014	Strong	Moderate	Strong	Strong	Strong	Strong	Strong
**Beaumont**	1976	Moderate	Moderate	Weak	Weak	Moderate	Strong	Weak
**Beheshti**	2018	Strong	Moderate	Strong	Strong	Strong	Strong	Strong
**Beliard**	2018	Strong	Moderate	Moderate	Weak	Strong	Strong	Moderate
**Benn**	2012	Strong	Moderate	Strong	Strong	Strong	Strong	Strong
**Berard**	2019	Strong	Moderate	Strong	Strong	Strong	Strong	Moderate
**Bertolini**	2000	Moderate	Moderate	Moderate	Weak	Strong	Strong	Moderate
**Bertolini**	2013	Moderate	Moderate	Moderate	Weak	Strong	Strong	Moderate
**Besseling**	2014	Strong	Moderate	Strong	Strong	Strong	Strong	Strong

**Bhatnagar**	2000	Moderate	Moderate	Moderate	Weak	Strong	Moderate	Moderate
**Bogsrud**	2019	Strong	Moderate	Moderate	Weak	Strong	Strong	Moderate
**Bowden**	1994	Moderate	Moderate	Moderate	Weak	Weak	Strong	Weak
**Carmena**	1996	Moderate	Moderate	Weak	Weak	Moderate	Moderate	Weak
**Chan**	2015	Strong	Moderate	Strong	Strong	Strong	Strong	Strong
**Coutinho**	2021	Strong	Moderate	Strong	Strong	Strong	Strong	Strong
**deGoma**	2016	Strong	Moderate	Strong	Strong	Strong	Strong	Moderate
**De Sauvage Nolting**	2003	Moderate	Strong	Strong	Strong	Strong	Strong	Strong
**Doi**	2021	Moderate	Moderate	Strong	Weak	Strong	Strong	Moderate
**Duell**	2019	Strong	Moderate	Strong	Strong	Strong	Strong	Moderate
**Ershova**	2017	Strong	Moderate	Strong	Strong	Strong	Strong	Strong
**Ferrieres**	1995	Moderate	Moderate	Strong	Weak	Strong	Strong	Moderate
**Firth**	2008	Moderate	Moderate	Moderate	Weak	Strong	Strong	Moderate
**Hill**	1991	Moderate	Moderate	Weak	Weak	Moderate	Weak	Weak
**Hirobe**	1982	Moderate	Moderate	Weak	Weak	Moderate	Moderate	Weak
**Holmes**	2005	Strong	Moderate	Strong	Weak	Moderate	Moderate	Moderate
**Hoogerbrugge**	1999	Moderate	Strong	Weak	Weak	Moderate	Moderate	Weak
**Hopkins**	2001	Strong	Moderate	Strong	Strong	Strong	Strong	Strong
**Hovland**	2018	Strong	Moderate	Strong	Strong	Strong	Strong	Strong
**Hu**	2013	Strong	Moderate	Moderate	Weak	Strong	Strong	Moderate
**Humphries**	2018	Strong	Moderate	Strong	Strong	Strong	Strong	Strong
**Iyen**	2019	Strong	Moderate	Strong	Strong	Strong	Strong	Strong
**Iyen**	2020	Strong	Moderate	Strong	Strong	Strong	Strong	Strong
**Jansen**	2004	Strong	Moderate	Strong	Strong	Strong	Strong	Strong
**Jung**	2018	Strong	Moderate	Strong	Strong	Strong	Strong	Strong
**Khoury**	2021	Strong	Moderate	Strong	Moderate	Strong	Strong	Strong
**Koeijvoets**	2006	Strong	Moderate	Strong	Strong	Strong	Strong	Strong
**Koeijvoets**	2008	Strong	Moderate	Strong	Strong	Strong	Strong	Strong

**Korneva**	2019	Moderate	Moderate	Strong	Strong	Weak	Weak	Weak
**Krogh**	2016	Strong	Moderate	Strong	Strong	Strong	Strong	Strong
**Latkovskis**	2018	Strong	Moderate	Weak	Moderate	Moderate	Moderate	Moderate
**Li**	2017	Moderate	Moderate	Strong	Weak	Strong	Strong	Moderate
**Mabuchi**	1977	Moderate	Moderate	Moderate	Weak	Strong	Strong	Moderate
**Macedo**	2008	Moderate	Moderate	Moderate	Weak	Strong	Strong	Moderate
**Masana**	2019	Strong	Moderate	Weak	Strong	Moderate	Strong	Moderate
**Mata**	2002	Moderate	Moderate	Moderate	Weak	Moderate	Strong	Moderate
**Michikura**	2017	Strong	Moderate	Strong	Weak	Strong	Strong	Moderate
**Miettinen**	1988	Moderate	Moderate	Weak	Weak	Moderate	Moderate	Weak
**Miname**	2019	Strong	Moderate	Strong	Strong	Strong	Strong	Strong
**Mohrschladt**	2004	Strong	Moderate	Moderate	Weak	Strong	Strong	Moderate
**Mundal**	2014	Strong	Moderate	Strong	Strong	Strong	Strong	Strong
**Mundal**	2016	Strong	Moderate	Moderate	Strong	Strong	Strong	Strong
**Mundal**	2018	Strong	Moderate	Weak	Strong	Strong	Strong	Moderate
**Neil**	2003	Strong	Moderate	Moderate	Strong	Strong	Strong	Strong
**Neil**	2004	Strong	Moderate	Moderate	Strong	Strong	Strong	Strong
**Neil**	2008	Strong	Moderate	Moderate	Strong	Strong	Strong	Strong
**Nenseter**	2010	Weak	Moderate	Moderate	Strong	Moderate	Moderate	Moderate
**Nybo**	2007	Moderate	Moderate	Moderate	Weak	Moderate	Strong	Moderate
**Panagiotakos**	2003	Strong	Moderate	Strong	Weak	Strong	Strong	Moderate
**Pang**	2018	Strong	Moderate	Strong	Strong	Strong	Strong	Strong
**Paquette**	2017	Moderate	Moderate	Strong	Moderate	Strong	Strong	Strong
**Paquette**	2017	Moderate	Moderate	Strong	Moderate	Strong	Strong	Strong
**Paquette**	2021	Strong	Moderate	Strong	Strong	Strong	Strong	Strong
**Pasta**	2020	Strong	Moderate	Weak	Moderate	Moderate	Strong	Moderate
**Perak**	2016	Strong	Moderate	Strong	Strong	Strong	Strong	Strong
**Pereira**	2014	Moderate	Moderate	Moderate	Weak	Moderate	Strong	Moderate

**Pereira**	2015	Moderate	Moderate	Moderate	Weak	Moderate	Strong	Moderate
**Perez de Isla**	2016	Strong	Moderate	Strong	Weak	Strong	Strong	Moderate
**Perez de Isla**	2017	Strong	Moderate	Strong	Weak	Strong	Strong	Moderate
**Pérez García**	2018	Strong	Moderate	Moderate	Weak	Strong	Strong	Moderate
**Pérez-Calahorra**	2017	Strong	Moderate	Moderate	Moderate	Strong	Strong	Strong
**Pisciotta**	2005	Strong	Moderate	Weak	Weak	Moderate	Strong	Weak
**Pitsavos**	2004	Strong	Moderate	Strong	Strong	Strong	Strong	Strong
**Ramos**	2020	Strong	Moderate	Strong	Strong	Moderate	Strong	Strong
**Ryzhaya**	2021	Strong	Moderate	Moderate	Strong	Strong	Strong	Strong
**Sánchez-Ramos**	2021	Strong	Moderate	Weak	Strong	Strong	Strong	Moderate
**Simon Broome†**	1991	Strong	Moderate	Moderate	Weak	Moderate	Strong	Moderate
**Simon Broome†**	1999	Strong	Moderate	Moderate	Weak	Moderate	Strong	Moderate
**Seed**	1990	Moderate	Moderate	Moderate	Weak	Moderate	Moderate	Moderate
**Silva**	2016	Strong	Moderate	Strong	Moderate	Weak	Strong	Moderate
**Simonen**	1987	Moderate	Moderate	Weak	Weak	Strong	Strong	Weak
**Slack**	1969	Weak	Moderate	Moderate	Weak	Moderate	Moderate	Weak
**Svendsen**	2021	Strong	Moderate	Strong	Strong	Strong	Strong	Strong
**Tada**	2021	Strong	Moderate	Strong	Strong	Strong	Strong	Strong
**Tada**	2023	Strong	Moderate	Strong	Strong	Strong	Strong	Strong
**Vallejo-Vaz**	2021	Strong	Moderate	Strong	Strong	Strong	Strong	Strong
**Versmissen**	2008	Strong	Moderate	Strong	Strong	Strong	Strong	Strong
**Vlad**	2021	Strong	Moderate	Strong	Weak	Strong	Strong	Moderate
**Vuorio**	1997	Strong	Moderate	Strong	Moderate	Strong	Strong	Strong
**Wierzbicki**	2000	Strong	Moderate	Strong	Weak	Strong	Strong	Moderate
**Yaman**	2020	Strong	Moderate	Strong	Weak	Weak	Strong	Weak
**Zamora**	2017	Strong	Moderate	Weak	Strong	Moderate	Strong	Moderate
**Zhao**	2019	Moderate	Moderate	Strong	Weak	Moderate	Moderate	Moderate

**16 Clinical Trials**								
**Braamskamp**	2015	Moderate	Strong	Weak	Moderate	Strong	Moderate	Moderate
**Budinsky**	2001	Weak	Weak	Weak	Weak	Moderate	Moderate	Weak
**Chaves**	2001	Moderate	Moderate	Moderate	Weak	Moderate	Moderate	Moderate
**De Knijff**	1990	Moderate	Weak	Weak	Weak	Moderate	Moderate	Weak
**Hoogerbrugge**	1999	Moderate	Strong	Weak	Weak	Moderate	Moderate	Weak
**Jeenah**	1993	Moderate	Moderate	Weak	Weak	Moderate	Moderate	Moderate
**Jerling**	1997	Moderate	Moderate	Weak	Weak	Moderate	Moderate	Moderate
**Kane**	1990	Moderate	Strong	Weak	Weak	Moderate	Strong	Moderate
**Kastelein**	2015	Strong	Strong	Moderate	Strong	Strong	Strong	Strong
**Leitersdorf**	1994	Moderate	Moderate	Weak	Weak	Moderate	Moderate	Moderate
**Miltiadous**	2005	Moderate	Moderate	Strong	Weak	Moderate	Moderate	Moderate
**Morisaki**	1990	Moderate	Moderate	Weak	Weak	Moderate	Moderate	Weak
**Raal**	2015	Strong	Strong	Moderate	Strong	Strong	Strong	Strong
**Sinzinger**	1994	Moderate	Moderate	Weak	Weak	Moderate	Strong	Moderate
**Smilde**	2000	Moderate	Moderate	Moderate	Weak	Moderate	Moderate	Moderate
**Stein**	2012	Strong	Strong	Moderate	Strong	Strong	Strong	Strong

Appendix 4

Appendix 4: Reference list of the 133 studies included in the qualitative synthesis from the systematic review of sex differences in the treatment of familial hypercholesterolemia.

1. Agarwala A, Bekele N, Deych E, et al. Racial Disparities in Modifiable Risk Factors and Statin Usage in Black Patients With Familial Hypercholesterolemia. *J Am Heart Assoc* 2021; **10**: e020890.

2. Agarwala A, Deych E, Jones LK, et al. Sex-related differences in premature cardiovascular disease in familial hypercholesterolemia. *J Clin Lipidol* 2023; **17**: 150-6.

3. Ahmad Z, Li X, Wosik J, et al. Premature coronary heart disease and autosomal dominant hypercholesterolemia: Increased risk in women with LDLR mutations. *J Clin Lipidol* 2016; **10**: 101-8.e1-3.

4. Allard MD, Saeedi R, Yousefi M, Frohlich J. Risk stratification of patients with familial hypercholesterolemia in a multi-ethnic cohort. *Lipids health dis* 2014; **13**: 65.

5. Alonso R, Mata N, Castillo S, et al. Cardiovascular disease in familial hypercholesterolaemia: influence of low-density lipoprotein receptor mutation type and classic risk factors. *Atherosclerosis* 2008; **200**: 315-21.

6. Alonso R, Andres E, Mata N, et al. Lipoprotein(a) levels in familial hypercholesterolemia: an important predictor of cardiovascular disease independent of the type of LDL receptor mutation. *J Am Coll Cardiol* 2014; **63**: 1982-9.

7. Alonso R, Muniz-Grijalvo O, Diaz-Diaz JL, et al. Efficacy of PCSK9 inhibitors in the treatment of heterozygous familial hypercholesterolemia: A clinical practice experience. *J Clin Lipidol* 2021; **15**: 584-92.

8. Amrock SM, Duell PB, Knickelbine T, et al. Health disparities among adult patients with a phenotypic diagnosis of familial hypercholesterolemia in the CASCADE-FH TM patient registry. *Atherosclerosis* 2017; **267**: 19-26.

9. Arnesen KE, Phung AV, Randsborg K, et al. Risk of Recurrent Coronary Events in Patients With Familial Hypercholesterolemia; A 10-Years Prospective Study. *Front Pharmacol* 2020; **11**: 560958.

10. Beaumont V, Jacotot B, Beaumont JL. Ischaemic disease in men and women with familial hypercholesterolaemia and xanthomatosis. A comparative study of genetic and environmental factors in 274 heterozygous cases. *Atherosclerosis* 1976; **24**: 441-50.

11. Beheshti S, Madsen CM, Varbo A, Benn M, Nordestgaard BG. Relationship of Familial Hypercholesterolemia and High Low-Density Lipoprotein Cholesterol to Ischemic Stroke: Copenhagen General Population Study. *Circulation* 2018; **138**: 578-89.

12. Beliard S, Carreau V, Carrie A, et al. Improvement in LDL-cholesterol levels of patients with familial hypercholesterolemia: can we do better? Analysis of results obtained during the past two decades in 1669 French subjects. *Atherosclerosis* 2014; **234**: 136-41.

13. Beliard S, Boccara F, Cariou B, et al. High burden of recurrent cardiovascular events in heterozygous familial hypercholesterolemia: The French Familial Hypercholesterolemia Registry. *Atherosclerosis* 2018; **277**: 334-40.

14. Benn M, Watts GF, Tybjaerg-Hansen A, Nordestgaard BG. Familial hypercholesterolemia in the danish general population: prevalence, coronary artery disease, and cholesterol-lowering medication. *J Clin Endocrinol Metab* 2012; **97**: 3956-64.

15. Berard E, Bongard V, Haas B, et al. Prevalence and Treatment of Familial Hypercholesterolemia in France. *Can J Cardiol* 2019; **35**: 744-52.

16. Bertolini S, Cantafora A, Averna M, et al. Clinical expression of familial hypercholesterolemia in clusters of mutations of the LDL receptor gene that cause a receptor-defective or receptor-negative phenotype. *Arterioscler Thromb Vasc Biol* 2000; **20**: E41-52.

17. Bertolini S, Pisciotta L, Rabacchi C, et al. Spectrum of mutations and phenotypic expression in patients with autosomal dominant hypercholesterolemia identified in Italy. *Atherosclerosis* 2013; **227**: 342-8.

18. Besseling J, Kindt I, Hof M, Kastelein JJ, Hutten BA, Hovingh GK. Severe heterozygous familial hypercholesterolemia and risk for cardiovascular disease: a study of a cohort of 14,000 mutation carriers. *Atherosclerosis* 2014; **233**: 219-23.

19. Bhatnagar D, Morgan J, Siddiq S, Mackness MI, Miller JP, Durrington PN. Outcome of case finding among relatives of patients with known heterozygous familial hypercholesterolaemia. *BMJ* 2000; **321**: 1497-500.

20. Bogsrud MP, Graesdal A, Johansen D, et al. LDL-cholesterol goal achievement, cardiovascular disease, and attributed risk of Lp(a) in a large cohort of predominantly genetically verified familial hypercholesterolemia. *J Clin Lipidol* 2019; **13**: 279-86.

21. Bowden JF, Pritchard PH, Hill JS, Frohlich JJ. Lp(a) concentration and apo(a) isoform size. Relation to the presence of coronary artery disease in familial hypercholesterolemia. *Arteriosclerosis & Thrombosis* 1994; **14**: 1561-8.

22. Braamskamp MJ, Kusters DM, Avis HJ, et al. Long-term statin treatment in children with familial hypercholesterolemia: more insight into tolerability and adherence. *Paediatr Drugs* 2015; **17**: 159-66.

23. Budinsky A, Wolfram R, Oguogho A, Efthimiou Y, Stamatopoulos Y, Sinzinger H. Regular ingestion of opuntia robusta lowers oxidation injury. *Prostaglandins Leukotrienes Essent Fatty Acids* 2001; **65**: 45-50.

24. Carmena R, Lussier-Cacan S, Roy M, et al. Lp(a) levels and atherosclerotic vascular disease in a sample of patients with familial hypercholesterolemia sharing the same gene defect. *Arterioscler Thromb Vasc Biol* 1996; **16**: 129-36.

25. Chan DC, Pang J, Hooper AJ, et al. Elevated lipoprotein(a), hypertension and renal insufficiency as predictors of coronary artery disease in patients with genetically confirmed heterozygous familial hypercholesterolemia. *Int J Cardiol* 2015; **201**: 633-8.

26. Chaves FJ, Real JT, Garcia-Garcia AB, et al. Genetic diagnosis of familial hypercholesterolemia in a South European outbreed population: influence of low-density lipoprotein (LDL) receptor gene mutations on treatment response to simvastatin in total, LDL, and high-density lipoprotein cholesterol. *J Clin Endocrinol Metab* 2001; **86**: 4926-32.

27. Coutinho ER, Miname MH, Rocha VZ, et al. Familial hypercholesterolemia and cardiovascular disease in older individuals. *Atherosclerosis* 2021; **318**: 32-7.

28. deGoma EM, Ahmad ZS, O'Brien EC, et al. Treatment Gaps in Adults With Heterozygous Familial Hypercholesterolemia in the United States: Data From the CASCADE-FH Registry. *Circ Cardiovasc Genet* 2016; **9**: 240-9.

29. De Knijff P, Stalenhoef AF, Mol MJ, et al. Influence of apo E polymorphism on the response to simvastatin treatment in patients with heterozygous familial hypercholesterolemia. *Atherosclerosis* 1990; **83**: 89-97.

30. de Sauvage Nolting PR, Defesche JC, Buirma RJ, Hutten BA, Lansberg PJ, Kastelein JJ. Prevalence and significance of cardiovascular risk factors in a large cohort of patients with familial hypercholesterolaemia. *J Intern Med* 2003; **253**: 161-8.

31. Doi T, Hori M, Harada-Shiba M, et al. Patients With LDLR and PCSK9 Gene Variants Experienced Higher Incidence of Cardiovascular Outcomes in Heterozygous Familial Hypercholesterolemia. *J Am Heart Assoc* 2021; **10**: e018263.

32. Duell PB, Gidding SS, Andersen RL, et al. Longitudinal low density lipoprotein cholesterol goal achievement and cardiovascular outcomes among adult patients with familial hypercholesterolemia: The CASCADE FH registry. *Atherosclerosis* 2019; **289**: 85-93.

33. Ershova AI, Meshkov AN, Bazhan SS, et al. The prevalence of familial hypercholesterolemia in the West Siberian region of the Russian Federation: A substudy of the ESSE-RF. *PLoS ONE* 2017; **12**: e0181148.

34. Ferrieres J, Lambert J, Lussier-Cacan S, Davignon J. Coronary artery disease in heterozygous familial hypercholesterolemia patients with the same LDL receptor gene mutation. *Circulation* 1995; **92**: 290-5.

35. Firth JC, Marais AD. Familial hypercholesterolaemia: the Cape Town experience. *S Afr Med J* 2008; **98**: 99-104.

36. Galema-Boers JM, Lenzen MJ, van Domburg RT, et al. Predicting non-adherence in patients with familial hypercholesterolemia. *Eur J Clin Pharmacol* 2014; **70**: 391-7.

37. Gallo A, Giral P, Carrie A, et al. Early coronary calcifications are related to cholesterol burden in heterozygous familial hypercholesterolemia. *J Clin Lipidol* 2017; **11**: 704-11 e2.

38. Hill JS, Hayden MR, Frohlich J, Pritchard PH. Genetic and environmental factors affecting the incidence of coronary artery disease in heterozygous familial hypercholesterolemia. *Arterioscler Thromb* 1991; **11**: 290-7.

39. Hirobe K, Matsuzawa Y, Ishikawa K, et al. Coronary artery disease in heterozygous familial hypercholesterolemia. *Atherosclerosis* 1982; **44**: 201-10.

40. Holmes DT, Schick BA, Humphries KH, Frohlich J. Lipoprotein(a) is an independent risk factor for cardiovascular disease in heterozygous familial hypercholesterolemia. *Clin Chem* 2005; **51**: 2067-73.

41. Hoogerbrugge N, Jansen H. Atorvastatin increases low-density lipoprotein size and enhances high-density lipoprotein cholesterol concentration in male, but not in female patients with familial hypercholesterolemia. *Atherosclerosis* 1999; **146**: 167-74.

42. Hopkins PN, Stephenson S, Wu LL, Riley WA, Xin Y, Hunt SC. Evaluation of coronary risk factors in patients with heterozygous familial hypercholesterolemia. *Am J Cardiol* 2001; **87**: 547-53.

43. Hovland A, Mundal LJ, Igland J, et al. Risk of Ischemic Stroke and Total Cerebrovascular Disease in Familial Hypercholesterolemia. *Stroke* 2019; **50**: 172-4.

44. Hu M, Lan W, Lam CWK, Mak YT, Pang CP, Tomlinson B. Heterozygous familial hypercholesterolemia in Hong Kong Chinese. Study of 252 cases. *International Journal of Cardiology* 2013; **167**: 762-7.

45. Humphries SE, Cooper JA, Seed M, et al. Coronary heart disease mortality in treated familial hypercholesterolaemia: Update of the UK Simon Broome FH register. *Atherosclerosis* 2018; **274**: 41-6.

46. Iyen B, Qureshi N, Kai J, et al. Risk of cardiovascular disease outcomes in primary care subjects with familial hypercholesterolaemia: A cohort study. *Atherosclerosis* 2019; **287**: 8-15.

47. Iyen B, Qureshi N, Weng S, et al. Sex differences in cardiovascular morbidity associated with familial hypercholesterolaemia: A retrospective cohort study of the UK Simon Broome register linked to national hospital records. *Atherosclerosis* 2020; **315**: 131-7.

48. Iyen B, Akyea RK, Weng S, Kai J, Qureshi N. Statin treatment and LDL-cholesterol treatment goal attainment among individuals with familial hypercholesterolaemia in primary care. *Open Heart* 2021; **8**.

49. Jackson CL, Deng Y, Yao X, Van Houten H, Shah ND, Kopecky S. Proprotein convertase subtilisin/kexin type 9 inhibitor utilization and low-density lipoprotein-cholesterol control in familial hypercholesterolemia. *J Clin Lipidol* 2021; **15**: 339-46.

50. Jansen AC, van Aalst-Cohen ES, Tanck MW, et al. The contribution of classical risk factors to cardiovascular disease in familial hypercholesterolaemia: data in 2400 patients. *J Intern Med* 2004; **256**: 482-90.

51. Jeenah M, September W, Graadt van Roggen F, de Villiers W, Seftel H, Marais D. Influence of specific mutations at the LDL-receptor gene locus on the response to simvastatin therapy in Afrikaner patients with heterozygous familial hypercholesterolaemia. *Atherosclerosis* 1993; **98**: 51-8.

52. Jerling JC, Vorster HH, Oosthuizen W, Vermaak WJH. Effect of simvastatin, a 3-hydroxy-3-methylglutaryl coenzyme A reductase inhibitor, on the haemostatic balance of familial hypercholesterolaemic subjects. *Fibrinolysis and Proteolysis* 1997; **11**: 91-6.

53. Jimenez A, Vinals C, Marco-Benedi V, et al. Sex Disparities in Familial Hypercholesterolemia. *J Am Coll Cardiol* 2023; **81**: 203-5.

54. Jung KJ, Koh H, Choi Y, Lee SJ, Ji E, Jee SH. Familial hypercholesterolemia and atherosclerotic cardiovascular mortality among Korean adults with low levels of serum cholesterol. *Atherosclerosis* 2018; **278**: 103-9.

55. Kane JP, Malloy MJ, Ports TA, Phillips NR, Diehl JC, Havel RJ. Regression of coronary atherosclerosis during treatment of familial hypercholesterolemia with combined drug regimens. *JAMA* 1990; **264**: 3007-12.

56. Kastelein JJ, Ginsberg HN, Langslet G, et al. ODYSSEY FH I and FH II: 78 week results with alirocumab treatment in 735 patients with heterozygous familial hypercholesterolaemia. *Eur Heart J* 2015; **36**: 2996-3003.

57. Khoury E, Brisson D, Roy N, Tremblay G, Gaudet D. Identifying Markers of Cardiovascular Event-Free Survival in Familial Hypercholesterolemia. *J Clin Med* 2021; **10**: 64.

58. Kłosiewicz-Latoszek L, Cybulska B, Białobrzeska-Paluszkiewicz J, et al. Clinical management of heterozygous familial hypercholesterolemia in a Polish outpatient metabolic clinic: A retrospective observational study. *Archives of Medical Science* 2018; **14**: 962-70.

59. Koeijvoets KC, van Rossum EF, Dallinga-Thie GM, et al. A functional polymorphism in the glucocorticoid receptor gene and its relation to cardiovascular disease risk in familial hypercholesterolemia. *J Clin Endocrinol Metab* 2006; **91**: 4131-6.

60. Koeijvoets KC, van der Net JB, van Rossum EF, et al. Two common haplotypes of the glucocorticoid receptor gene are associated with increased susceptibility to cardiovascular disease in men with familial hypercholesterolemia. *J Clin Endocrinol Metab* 2008; **93**: 4902-8.

61. Korneva V, Kuznetsova T, Julius U. Efficiency and problems of statin therapy in patients with heterozygous familial hypercholesterolemia. *Atheroscler Suppl* 2019; **40**: 79-87.

62. Krogh HW, Mundal L, Holven KB, Retterstol K. Patients with familial hypercholesterolaemia are characterized by presence of cardiovascular disease at the time of death. *Eur Heart J* 2016; **37**: 1398-405.

63. Latkovskis G, Saripo V, Gilis D, Nesterovics G, Upena-Roze A, Erglis A. Latvian registry of familial hypercholesterolemia: The first report of three-year results. *Atherosclerosis* 2018; **277**: 347-54.

64. Leduc V, Bourque L, Poirier J, Dufour R. Role of rs3846662 and HMGCR alternative splicing in statin efficacy and baseline lipid levels in familial hypercholesterolemia. *Pharmacogenet Genomics* 2016; **26**: 1-11.

65. Leitersdorf E. Gender-related response to fluvastatin in patients with heterozygous familial hypercholesterolaemia. *Drugs* 1994; **47 Suppl 2**: 54-8.

66. Li JJ, Li S, Zhu CG, et al. Familial Hypercholesterolemia Phenotype in Chinese Patients Undergoing Coronary Angiography. *Arterioscler Thromb Vasc Biol* 2017; **37**: 570-9.

67. Mabuchi H, Haba T, Ueda K, et al. Serum lipids and coronary heart disease in heterozygous familial hypercholesterolemia in the Hokuriku District of Japan. *Atherosclerosis* 1977; **28**: 417-23.

68. Macedo A, Sebastiao KI, Miname MH, Santos RD. Risk factors for coronary heart disease in Brazilian familial hypercholesterolemia subjects. *International Journal of Atherosclerosis* 2008; **3**: 87-92.

69. Masana L, Zamora A, Plana N, et al. Incidence of Cardiovascular Disease in Patients with Familial Hypercholesterolemia Phenotype: Analysis of 5 Years Follow-Up of Real-World Data from More than 1.5 Million Patients. *J Clin Med* 2019; **8**.

70. Mata P, Alonso R, Castillo S, Pocovi M, Spanish Group of Familial H. MEDPED and the Spanish Familial Hypercholesterolemia Foundation. *Atheroscler Suppl* 2002; **2**: 9-11.

71. Matta MG, Saenz B, Schreier L, Corral A, Sarobe A, Corral P. Use and persistence of lipid-lowering therapy in patients with severe hypercholesterolemia: A prospective study. *Clinica Investigacion Arteriosclerosis* 2021; **33(6)**: 308-13.

72. Mattina A, Giammanco A, Giral P, et al. Polyvascular subclinical atherosclerosis in familial hypercholesterolemia: The role of cholesterol burden and gender. *Nutr Metab Cardiovasc Dis* 2019; **29**: 1068-76.

73. Michikura M, Ogura M, Yamamoto M, et al. Achilles Tendon Ultrasonography for Diagnosis of Familial Hypercholesterolemia Among Japanese Subjects. *Circ J* 2017; **81**: 1879-85.

74. Miettinen TA, Gylling H. Mortality and cholesterol metabolism in familial hypercholesterolemia. Long-term follow-up of 96 patients. *Arteriosclerosis* 1988; **8**: 163-7.

75. Miltiadous G, Xenophontos S, Bairaktari E, Ganotakis M, Cariolou M, Elisaf M. Genetic and environmental factors affecting the response to statin therapy in patients with molecularly defined familial hypercholesterolaemia. *Pharmacogenet Genomics* 2005; **15**: 219-25.

76. Miname MH, Bittencourt MS, Moraes SR, et al. Coronary Artery Calcium and Cardiovascular Events in Patients With Familial Hypercholesterolemia Receiving Standard Lipid-Lowering Therapy. *JACC Cardiovasc Imaging* 2019; **12**: 1797-804.

77. Mohrschladt MF, Westendorp RG, Gevers Leuven JA, Smelt AH. Cardiovascular disease and mortality in statin-treated patients with familial hypercholesterolemia. *Atherosclerosis* 2004; **172**: 329-35.

78. Morisaki N, Kobayashi J, Ishikawa Y, et al. Effects of Long-Term Treatment with Probucol on Serum Lipoproteins in Cases of Familial Hypercholesterolemia in the Elderly. *Journal of the American Geriatrics Society* 1990; **38**: 15-8.

79. Mundal L, Sarancic M, Ose L, et al. Mortality among patients with familial hypercholesterolemia: a registry-based study in Norway, 1992-2010. *J Am Heart Assoc* 2014; **3**: e001236.

80. Mundal L, Veierod MB, Halvorsen T, et al. Cardiovascular disease in patients with genotyped familial hypercholesterolemia in Norway during 1994-2009, a registry study. *Eur J Prev Cardiol* 2016; **23**: 1962-9.

81. Mundal LJ, Igland J, Veierod MB, et al. Impact of age on excess risk of coronary heart disease in patients with familial hypercholesterolaemia. *Heart* 2018; **104**: 1600-7.

82. Neil HA, Huxley RR, Hawkins MM, et al. Comparison of the risk of fatal coronary heart disease in treated xanthomatous and non-xanthomatous heterozygous familial hypercholesterolaemia: a prospective registry study. *Atherosclerosis* 2003; **170**: 73-8.

83. Neil HA, Seagroatt V, Betteridge DJ, et al. Established and emerging coronary risk factors in patients with heterozygous familial hypercholesterolaemia. *Heart* 2004; **90**: 1431-7.

84. Neil A, Cooper J, Betteridge J, et al. Reductions in all-cause, cancer, and coronary mortality in statin-treated patients with heterozygous familial hypercholesterolaemia: A prospective registry study. *Eur Heart J* 2008; **29**: 2625-33.

85. Nenseter MS, Lindvig HW, Ueland T, et al. Lipoprotein(a) levels in coronary heart disease-susceptible and -resistant patients with familial hypercholesterolemia. *Atherosclerosis* 2011; **216**: 426-32.

86. Nybo M, Brusgaard K, Hansen AB. No certain predictors for mutation status in a Danish cohort with familial hypercholesterolemia: a descriptive study. *Clinical Biochemistry* 2007; **40**: 1347-52.

87. Panagiotakos DB, Pitsavos C, Skoumas J, et al. Importance of LDL/HDL cholesterol ratio as a predictor for coronary heart disease events in patients with heterozygous familial hypercholesterolaemia: a 15-year follow-up (1987-2002). *Curr Med Res Opin* 2003; **19**: 89-94.

88. Pang J, David Marais A, Blom DJ, et al. Heterozygous familial hypercholesterolaemia in specialist centres in South Africa, Australia and Brazil: Importance of early detection and lifestyle advice. *Atherosclerosis* 2018; **277**: 470-6.

89. Pang J, Sullivan DR, Hare DL, et al. Gaps in the Care of Familial Hypercholesterolaemia in Australia: First Report From the National Registry. *Heart Lung Circ* 2021; **30(3)**: 372-9.

90. Paquette M, Dufour R, Baass A. The Montreal-FH-SCORE: A new score to predict cardiovascular events in familial hypercholesterolemia. *J Clin Lipidol* 2017; **11**: 80-6.

91. Paquette M, Brisson D, Dufour R, Khoury E, Gaudet D, Baass A. Cardiovascular disease in familial hypercholesterolemia: Validation and refinement of the Montreal-FH-SCORE. *J Clin Lipidol* 2017; **11**: 1161-7 e3.

92. Paquette M, Bernard S, Cariou B, et al. Familial Hypercholesterolemia-Risk-Score: A New Score Predicting Cardiovascular Events and Cardiovascular Mortality in Familial Hypercholesterolemia. *Arterioscler Thromb Vasc Biol* 2021; **41**: 2632-40.

93. Pasta A, Cremonini AL, Formisano E, Fresa R, Bertolini S, Pisciotta L. Long term follow-up of genetically confirmed patients with familial hypercholesterolemia treated with first and second-generation statins and then with PCSK9 monoclonal antibodies. *Atherosclerosis* 2020; **308**: 6-14.

94. Perak AM, Ning H, de Ferranti SD, Gooding HC, Wilkins JT, Lloyd-Jones DM. Long-Term Risk of Atherosclerotic Cardiovascular Disease in US Adults With the Familial Hypercholesterolemia Phenotype. *Circulation* 2016; **134**: 9-19.

95. Pereira C, Miname M, Makdisse M, Kalil Filho R, Santos RD. Association of peripheral arterial and cardiovascular diseases in familial hypercholesterolemia. *Arq Bras Cardiol* 2014; **103**: 118-23.

96. Pereira C, Miname MH, Makdisse MRP, et al. Peripheral arterial disease in heterozygous familial hypercholesterolemia. *Atherosclerosis* 2015; **242**: 174-8.

97. Perez-Calahorra S, Sanchez-Hernandez RM, Plana N, et al. Value of the Definition of Severe Familial Hypercholesterolemia for Stratification of Heterozygous Patients. *Am J Cardiol* 2017; **119**: 742-8.

98. Perez de Isla L, Alonso R, Watts GF, et al. Attainment of LDL-Cholesterol Treatment Goals in Patients With Familial Hypercholesterolemia: 5-Year SAFEHEART Registry Follow-Up. *J Am Coll Cardiol* 2016; **67**: 1278-85.

99. Perez de Isla L, Alonso R, Mata N, et al. Predicting Cardiovascular Events in Familial Hypercholesterolemia: The SAFEHEART Registry (Spanish Familial Hypercholesterolemia Cohort Study). *Circulation* 2017; **135**: 2133-44.

100. Perez Garcia L. Familial hypercholesterolemia: Experience in the Lipid Clinic of Alava. *Clinica Investigacion Arteriosclerosis* 2018; **30**: 224-9.

101. Pijlman AH, Huijgen R, Verhagen SN, et al. Evaluation of cholesterol lowering treatment of patients with familial hypercholesterolemia: a large cross-sectional study in The Netherlands. *Atherosclerosis* 2010; **209**: 189-94.

102. Pisciotta L, Cortese C, Gnasso A, et al. Serum homocysteine, methylenetetrahydrofolate reductase gene polymorphism and cardiovascular disease in heterozygous familial hypercholesterolemia. *Atherosclerosis* 2005; **179**: 333-8.

103. Pitsavos CH, Chrysohoou C, Panagiotakos DB, et al. Exercise capacity and heart rate recovery as predictors of coronary heart disease events, in patients with heterozygous Familial Hypercholesterolemia. *Atherosclerosis* 2004; **173**: 347-52.

104. Raal FJ, Stein EA, Dufour R, et al. PCSK9 inhibition with evolocumab (AMG 145) in heterozygous familial hypercholesterolaemia (RUTHERFORD-2): a randomised, double-blind, placebo-controlled trial. *Lancet* 2015; **385**: 331-40.

105. Ramos R, Masana L, Comas-Cufi M, et al. Derivation and validation of SIDIAP-FHP score: A new risk model predicting cardiovascular disease in familial hypercholesterolemia phenotype. *Atherosclerosis* 2020; **292**: 42-51.

106. Razek O, Cermakova L, Armani H, et al. Attainment of Recommended Lipid Targets in Patients With Familial Hypercholesterolemia: Real-World Experience With PCSK9 Inhibitors. *Canadian Journal of Cardiology* 2018; **34**: 1004-9.

107. Roy G, Couture P, Genest J, et al. Influence of the LDL-Receptor Genotype on Statin Response in Heterozygous Familial Hypercholesterolemia: Insights From the Canadian FH Registry. *Canadian Journal of Cardiology* 2022; **38**: 311-9.

108. Ryzhaya N, Cermakova L, Trinder M, et al. Sex Differences in the Presentation, Treatment, and Outcome of Patients With Familial Hypercholesterolemia. *J Am Heart Assoc* 2021; **10**: e019286.

109. Sanchez-Ramos A, Fernandez-Labandera C, Vallejo-Vaz AJ, et al. Prevalence of familial hypercholesterolemia phenotype and ten-year risk of cardiovascular events in a working population in primary prevention: The ICARIA study. *Atherosclerosis* 2021; **338**: 39-45.

110. Schreuder MM, Hamkour S, Siegers KE, et al. LDL cholesterol targets rarely achieved in familial hypercholesterolemia patients: A sex and gender-specific analysis. *Atherosclerosis* 2023.

111. Seed M, Hoppichler F, Reaveley D, et al. Relation of serum lipoprotein(a) concentration and apolipoprotein(a) phenotype to coronary heart disease in patients with familial hypercholesterolemia. *N Engl J Med* 1990; **322**: 1494-9.

112. Silva PR, Jannes CE, Marsiglia JD, Krieger JE, Santos RD, Pereira AC. Predictors of cardiovascular events after one year of molecular screening for Familial hypercholesterolemia. *Atherosclerosis* 2016; **250**: 144-50.

113. Simon Broome Register. Risk of fatal coronary heart disease in familial hypercholesterolaemia. Scientific Steering Committee on behalf of the Simon Broome Register Group. *BMJ* 1991; **303**: 893-6.

114. Simon Broome Register. Mortality in treated heterozygous familial hypercholesterolaemia: implications for clinical management. Scientific Steering Committee on behalf of the Simon Broome Register Group. *Atherosclerosis* 1999; **142**: 105-12.

115. Simonen H, Miettinen TA. Coronary artery disease and bile acid synthesis in familial hypercholesterolemia. *Atherosclerosis* 1987; **63**: 159-66.

116. Sinzinger H, Pirich C. The RED-LIP study - pravastatin in primary isolated hypercholesterolemia - an open, prospective, multicenter trial. *Wiener Klinische Wochenschrift* 1994; **106**: 721-7.

117. Slack J. Risks of ischaemic heart-disease in familial hyperlipoproteinaemic states. *Lancet* 1969; **2**: 1380-2.

118. Smilde TJ, van den Berkmortel FW, Wollersheim H, van Langen H, Kastelein JJ, Stalenhoef AF. The effect of cholesterol lowering on carotid and femoral artery wall stiffness and thickness in patients with familial hypercholesterolaemia. *European Journal of Clinical Investigation* 2000; **30**: 473-80.

119. Stein EA, Dufour R, Gagne C, et al. Apolipoprotein B synthesis inhibition with mipomersen in heterozygous familial hypercholesterolemia: results of a randomized, double-blind, placebo-controlled trial to assess efficacy and safety as add-on therapy in patients with coronary artery disease. *Circulation* 2012; **126**: 2283-92.

120. Svendsen K, Krogh HW, Igland J, et al. 2.5-fold increased risk of recurrent acute myocardial infarction with familial hypercholesterolemia. *Atherosclerosis* 2021; **319**: 28-34.

121. Tada H, Okada H, Nomura A, et al. Prognostic impact of cascade screening for familial hypercholesterolemia on cardiovascular events. *J Clin Lipidol* 2021; **15**: 358-65.

122. Tada H, Nomura A, Nohara A, et al. Attainment of the low-density lipoprotein cholesterol treatment target and prognosis of heterozygous familial hypercholesterolemia. *Atherosclerosis* 2023; **371**: 61-6.

123. Vallejo-Vaz AJ, Ginsberg HN, Davidson MH, et al. Lower On-Treatment Low-Density Lipoprotein Cholesterol and Major Adverse Cardiovascular Events in Women and Men: Pooled Analysis of 10 ODYSSEY Phase 3 Alirocumab Trials. *J Am Heart Assoc* 2018; **7**: e009221.

124. Vallejo-Vaz AJ, Stevens CAT, Lyons ARM, et al. Global perspective of familial hypercholesterolaemia: a cross-sectional study from the EAS Familial Hypercholesterolaemia Studies Collaboration (FHSC). *Lancet* 2021; **398**: 1713-25.

125. Versmissen J, Oosterveer DM, Yazdanpanah M, et al. Efficacy of statins in familial hypercholesterolaemia: a long term cohort study. *BMJ* 2008; **337**: a2423.

126. Vlad CE, Foia L, Florea L, et al. Evaluation of cardiovascular risk factors in patients with familial hypercholesterolemia from the North-Eastern area of Romania. *Lipids health dis* 2021; **20**: 4.

127. Vuorio AF, Turtola H, Piilahti KM, Repo P, Kanninen T, Kontula K. Familial hypercholesterolemia in the Finnish north Karelia. A molecular, clinical, and genealogical study. *Arterioscler Thromb Vasc Biol* 1997; **17**: 3127-38.

128. Walus-Miarka M, Czarnecka D, Kloch-Badelek M, Wojciechowska W, Kapusta M, Malecki MT. Carotid artery plaques - Are risk factors the same in men and women with familial hypercholesterolemia? *Int J Cardiol* 2017; **244**: 290-5.

129. Wierzbicki AS, Lambert-Hammill M, Lumb PJ, Crook MA. Renin-angiotensin system polymorphisms and coronary events in familial hypercholesterolemia. *Hypertension* 2000; **36**: 808-12.

130. Yaman S, Ozdemir D, Akman BT, Cakir B, Ersoy O. Awareness, treatment rates, and compliance to treatment in patients with serum LDL cholesterol higher than 250 mg/dL, and possible, probable, or definite familial hypercholesterolemia. *Postgrad Med* 2021; **133**: 146-53.

131. Zamora A, Masana L, Comas-Cufi M, et al. Familial hypercholesterolemia in a European Mediterranean population-Prevalence and clinical data from 2.5 million primary care patients. *J Clin Lipidol* 2017; **11**: 1013-22.

132. Zamora A, Ramos R, Comas-Cufi M, et al. Women with familial hypercholesterolemia phenotype are undertreated and poorly controlled compared to men. *Sci Rep* 2023; **13**: 1492.

133. Zhao PJ, Ban MR, Iacocca MA, McIntyre AD, Wang J, Hegele RA. Genetic Determinants of Myocardial Infarction Risk in Familial Hypercholesterolemia. *CJC Open* 2019; **1**: 225-30.
